# The European guideline on management of major bleeding and coagulopathy following trauma: sixth edition

**DOI:** 10.1186/s13054-023-04327-7

**Published:** 2023-03-01

**Authors:** Rolf Rossaint, Arash Afshari, Bertil Bouillon, Vladimir Cerny, Diana Cimpoesu, Nicola Curry, Jacques Duranteau, Daniela Filipescu, Oliver Grottke, Lars Grønlykke, Anatole Harrois, Beverley J. Hunt, Alexander Kaserer, Radko Komadina, Mikkel Herold Madsen, Marc Maegele, Lidia Mora, Louis Riddez, Carolina S. Romero, Charles-Marc Samama, Jean-Louis Vincent, Sebastian Wiberg, Donat R. Spahn

**Affiliations:** 1 Department of Anaesthesiology, University Hospital Aachen, RWTH, Aachen University, Pauwelsstrasse 30, D-52074 Aachen, Germany; 2 Department of Paediatric and Obstetric Anaesthesia, Rigshospitalet, University of Copenhagen, Blegdamsvej 9, DK-2100 Copenhagen, Denmark; 3 Department of Trauma and Orthopaedic Surgery, Cologne-Merheim Medical Centre (CMMC), University of Witten/Herdecke, Ostmerheimer Strasse 200, D-51109 Cologne, Germany; 4 Department of Anaesthesiology, Perioperative Medicine and Intensive Care, Masaryk Hospital, J.E. Purkinje University, Socialni pece 3316/12A, CZ-40113 Usti nad Labem, Czech Republic; 5 Department of Anaesthesiology and Intensive Care Medicine, Charles University Faculty of Medicine, Simkova 870, CZ-50003 Hradec Králové, Czech Republic; 6 Department of Emergency Medicine, Emergency County Hospital “Sf. Spiridon” Iasi, University of Medicine and Pharmacy ”Grigore T. Popa” Iasi, Blvd. Independentei 1, RO-700111 Iasi, Romania; 7 Oxford Haemophilia and Thrombosis Centre, Nuffield Orthopaedic Centre, Oxford University Hospitals NHS Trust, Windmill Road, Oxford, OX3 7HE UK; 8 Radcliffe Department of Medicine, Oxford University, Oxford, UK; 9 Department of Anesthesiology, Intensive Care and Perioperative Medicine, Assistance Publique Hôpitaux de Paris, Paris Saclay University, 78 rue du Général Leclerc, F-94275 Le Kremlin-Bicêtre Cedex, France; 10 Department of Cardiac Anaesthesia and Intensive Care, “Prof. Dr. C. C. Iliescu” Emergency Institute of Cardiovascular Diseases, Carol Davila University of Medicine and Pharmacy, Sos Fundeni 256-258, RO-022328 Bucharest, Romania; 11 Department of Thoracic Anaesthesiology, The Heart Centre, Rigshospitalet, University of Copenhagen, Blegdamsvej 9, DK-2100 Copenhagen, Denmark; 12 Thrombosis and Haemophilia Centre, Guy’s and St Thomas’ NHS Foundation Trust, Westminster Bridge Road, London, SE1 7EH UK; 13 Institute of Anaesthesiology, University Hospital Zurich, Raemistrasse 100, CH-8091 Zurich, Switzerland; 14 Department of Traumatology, General and Teaching Hospital Celje, Medical Faculty, Ljubljana University, Oblakova ulica 5, SI-3000 Celje, Slovenia; 15 Department of Trauma and Orthopaedic Surgery, Cologne-Merheim Medical Centre (CMMC), Institute for Research in Operative Medicine (IFOM), University of Witten/Herdecke, Ostmerheimer Strasse 200, D-51109 Cologne, Germany; 16 Department of Anaesthesiology, Intensive Care and Pain Clinic, Vall d’Hebron Trauma, Rehabilitation and Burns Hospital, Autonomous University of Barcelona, Passeig de la Vall d’Hebron 119-129, ES-08035 Barcelona, Spain; 17 Department of Surgery and Trauma, Karolinska University Hospital, S-171 76 Solna, Sweden; 18 Department of Anaesthesia, Intensive Care and Pain Therapy, Consorcio Hospital General Universitario de Valencia, Universidad Europea of Valencia Methodology Research Department, Avenida Tres Cruces 2, ES-46014 Valencia, Spain; 19 Department of Anaesthesia, Intensive Care and Perioperative Medicine, GHU AP-HP Centre - Université Paris Cité - Cochin Hospital, 27 rue du Faubourg St. Jacques, F-75014 Paris, France; 20 Department of Intensive Care, Erasme University Hospital, Université Libre de Bruxelles, Route de Lennik 808, B-1070 Brussels, Belgium

**Keywords:** Emergency medicine, Trauma, Traumatic coagulopathy, Major bleeding, Haemostasis, Practice guideline, Diagnostics, Management

## Abstract

**Background:**

Severe trauma represents a major global public health burden and the management of post-traumatic bleeding continues to challenge healthcare systems around the world. Post-traumatic bleeding and associated traumatic coagulopathy remain leading causes of potentially preventable multiorgan failure and death if not diagnosed and managed in an appropriate and timely manner. This sixth edition of the European guideline on the management of major bleeding and coagulopathy following traumatic injury aims to advise clinicians who care for the bleeding trauma patient during the initial diagnostic and therapeutic phases of patient management.

**Methods:**

The pan-European, multidisciplinary Task Force for Advanced Bleeding Care in Trauma included representatives from six European professional societies and convened to assess and update the previous version of this guideline using a structured, evidence-based consensus approach. Structured literature searches covered the period since the last edition of the guideline, but considered evidence cited previously. The format of this edition has been adjusted to reflect the trend towards concise guideline documents that cite only the highest-quality studies and most relevant literature rather than attempting to provide a comprehensive literature review to accompany each recommendation.

**Results:**

This guideline comprises 39 clinical practice recommendations that follow an approximate temporal path for management of the bleeding trauma patient, with recommendations grouped behind key decision points. While approximately one-third of patients who have experienced severe trauma arrive in hospital in a coagulopathic state, a systematic diagnostic and therapeutic approach has been shown to reduce the number of preventable deaths attributable to traumatic injury.

**Conclusion:**

A multidisciplinary approach and adherence to evidence-based guidelines are pillars of best practice in the management of severely injured trauma patients. Further improvement in outcomes will be achieved by optimising and standardising trauma care in line with the available evidence across Europe and beyond.

**Supplementary Information:**

The online version contains supplementary material available at 10.1186/s13054-023-04327-7.

## Key messages

- Immediate detection and management of traumatic coagulopathy improves outcomes of severely injured patients.
- This guideline follows management of the severe trauma patient in chronological order, with a focus on prevention of possible exsanguination.
- These structured recommendations support measures that prioritise the optimisation of resources for the benefit of bleeding control based on scientific evidence.
- Empirical management should not be implemented unless no method of monitoring bleeding and coagulation is available.
- Optimal organisation of the resuscitation team for the bleeding trauma patient includes implementation of these guidelines.

**Supplementary Information:**

The online version contains supplementary material available at 10.1186/s13054-023-04327-7.

## Background

Severe trauma represents a major global public health burden. The Global Burden of Diseases, Injuries, and Risk Factors Study (GBD 2017), estimated that trauma accounted for 8% of total deaths annually [1]. Moreover, among adolescents aged 10–24 years, road injuries, self-harm and interpersonal violence are top causes of disability-adjusted life-years; in the 25–49 year age group road injuries are ranked first [2]. Post-traumatic bleeding and associated traumatic coagulopathy remain leading causes of potentially preventable multiorgan failure and death if not diagnosed and managed in an appropriate and timely manner [3].

Approximately one-third of patients who have experienced severe trauma arrive in hospital in a coagulopathic state, and a systematic diagnostic and therapeutic approach has been shown to reduce the number of preventable deaths attributable to traumatic injury [3–5]. This guideline aims to provide an evidence-based set of recommendations to advise clinicians who care for the bleeding trauma patient during the initial diagnostic and therapeutic phases of patient management.

## Methods

A panel of experts comprising the pan-European, multidisciplinary Task Force for Advanced Bleeding Care in Trauma convened to assess and update the previous version of this guideline [6] in light of the latest available published evidence on the management of bleeding and coagulopathy following traumatic injury. The author group comprises experts in the fields of emergency medicine, surgery, anaesthesiology, haematology and intensive care medicine, including representatives from six European professional societies: European Society of Anaesthesiology and Intensive Care (ESAIC), European Society of Intensive Care Medicine (ESICM), European Shock Society (ESS), European Society for Trauma and Emergency Surgery (ESTES), European Society for Emergency Medicine (EuSEM) and the Network for Advancement of Patient Blood Management, Haemostasis and Thrombosis (NATA).

Following a web conference in May 2021, scientific queries of interest were defined by the authors and formulated in PICO (Population/Intervention/Comparison/Outcome) format by one author (CSR) in consultation with the methodologist (AA) (Additional file 1). Literature search bundles and corresponding structured search strategies were developed and applied to Medline (OvidSP), Cochrane Central Register of Controlled Trials (CENTRAL) and Epistemonikos databases by a Cochrane trial search specialist. Landmark publications were used to refine literature search bundles and database searches were restricted to 01 Jan 2018 to 02 Dec 2021 for existing recommendations and from 01 January 2001 to 02 December 2021 for new topics. Identified de-duplicated abstracts were pre-screened by a subset of authors (LG, MHM, SW) and pre-selected abstracts from relevant search bundles were screened by each author (Additional file 2). Relevant full-text articles were retrieved and assessed in detail. Literature cited within identified articles and the previous edition of the guideline as well as relevant subsequent publications were also considered.

The authors aimed to include a restricted number of supporting references to support each recommendation as part of a brief accompanying rationale, with studies of the best available quality from any publication date given priority. Recommendations were formulated and graded according the Grading of Recommendations Assessment, Development and Evaluation (GRADE) system (Table 1) [7]. Recommendations, grading and rationales were initially drafted and critically reviewed by at least two authors and then distributed to the entire author group for review prior to the live consensus process. The quality of the literature cited to support each recommendation was reviewed separately by the methodologist (AA), who applied Cochrane risk-of-bias assessment criteria [8] and/or the SIGN checklist (https://www.sign.ac.uk/what-we-do/methodology/checklists/) for observational studies to the evaluation.

**Table 1:** Grading of recommendations after [7]

Grade of recommendation	Clarity of risk/benefit	Quality of supporting evidence	Implications
*1A*			
Strong recommendation, high-quality evidence	Benefits clearly outweigh risk and burdens, or vice versa	RCTs without important limitations or overwhelming evidence from observational studies	Strong recommendation, can apply to most patients in most circumstances without reservation
*1B*			
Strong recommendation, moderate-quality evidence	Benefits clearly outweigh risk and burdens, or vice versa	RCTs with important limitations (inconsistent results, methodological flaws, indirect or imprecise) or exceptionally strong evidence from observational studies	Strong recommendation, can apply to most patients in most circumstances without reservation
*1C*			
Strong recommendation, low-quality or very low-quality evidence	Benefits clearly outweigh risk and burdens, or vice versa	Observational studies or case series	Strong recommendation but may change when higher-quality evidence becomes available
*2A*			
Weak recommendation, high-quality evidence	Benefits closely balanced with risks and burden	RCTs without important limitations or overwhelming evidence from observational studies	Weak recommendation, best action may differ depending on circumstances or patients’ or societal values
*2B*			
Weak recommendation, moderate-quality evidence	Benefits closely balanced with risks and burden	RCTs with important limitations (inconsistent results, methodological flaws, indirect or imprecise) or exceptionally strong evidence from observational studies	Weak recommendation, best action may differ depending on circumstances or patients’ or societal values
*2C*			
Weak recommendation, Low-quality or very low-quality evidence	Uncertainty in the estimates of benefits, risks and burden; benefits, risk and burden may be closely balanced	Observational studies or case series	Very weak recommendation; other alternatives may be equally reasonable

*RCT* Randomised controlled trial

Authors participated in a series of virtual consensus conferences in May and June 2022, during which the wording and grading of each recommendation were finalised and confirmed by voting members of the expert panel (RR, AA, BB, VC, DC, NC, JD, DF, OG, AH, BJH, AK, RK, MM, LM, LR, CMS, JLV, DRS). Grading was confirmed and disagreements resolved in consultation with the methodologist (AA). Following final revisions, manuscript collation and approval by the author group, the manuscript was peer-reviewed and approved by the endorsing professional societies between September and November 2022.

## Results

### I. Initial resuscitation and prevention of further bleeding

#### Minimal elapsed time

##### Recommendation 1

We recommend that severely injured patients be transported directly to an appropriate trauma facility (Grade 1B).

We recommend that the time elapsed between injury and bleeding control be minimised (Grade 1B).

#### Rationale

Regionalisation of trauma management, with designated trauma centres that offer different levels of care and that interact with both each other and pre-hospital emergency medical services have improved trauma care in many countries. Several have also implemented trauma quality improvement programmes and continuously evaluate their results using trauma registries. In a recent systematic review and meta-analysis of 52 studies that included 1,106,431 trauma patients, the effectiveness of such trauma care systems was evaluated [9]. The study showed a significant reduction in mortality after implementation of trauma systems and demonstrated that survival improved when systems were further developed. In a retrospective multicentre cohort study of the Quebec trauma registry the impact of trauma centre designation level on outcomes following haemorrhagic shock (systolic blood pressure < 90 mmHg) was studied [10]. Level I trauma centres showed significantly lower standardised mortality rates among bleeding trauma patients compared with level III and IV trauma centres. This study supports the idea that “systemised” trauma care that matches each patient to the most appropriate treatment facility in a timely manner is advantageous, whereby the definition of “appropriate” depends on patient vital status, the nature of the injuries and the hospital facilities available.

There is a consensus that trauma patients in need of emergency intervention for ongoing haemorrhage have increased chances of survival if the elapsed time between injury and start of the intervention is minimised. In a recent study of early haemorrhagic trauma deaths, 34.5% were classified as potentially preventable by stopping bleeding early [11]. Time to intervention can be lost in the pre-hospital and early in-hospital settings. A retrospective analysis of a National Emergency Medical services Information System (NEMSIS) with 2,018,141 patients revealed that increased scene time was associated with greater mortality for blunt and penetrating trauma [12]. A study in penetrating trauma showed that every additional minute in pre-hospital response time correlated with a 2% increase in mortality, and every additional minute in pre-hospital scene time correlated with a 1% increase in mortality [13]. In a systematic review of the influence of pre-hospital time on outcome of trauma patients, the authors found that a rapid transportation of haemodynamically unstable patients with penetrating injuries was beneficial [14]. In haemodynamically stable patients, mortality did not correlate with increased pre-hospital times. Another study demonstrated that longer pre-hospital times in trauma patients did not increase 30-day mortality but were associated with an increased risk of poor functional outcome [15]. Several authors outline that it is important to minimise the time from injury to intervention in trauma patients with ongoing bleeding, be it surgery or embolisation [16, 17]. This means that not only is swift pre-hospital care of the essence, but timely in-hospital trauma management (door-to-needle time) as well.

#### Local bleeding management

##### Recommendation 2

We recommend local compression of open wounds to limit life-threatening bleeding (Grade 1B).

We recommend adjunct tourniquet use to stop life-threatening bleeding from open extremity injuries in the pre-surgical setting (Grade 1B).

#### Rationale

Most life-threatening bleeding from open injuries to extremities observed in the civilian setting can be controlled by local compression, either by manual compression or pressure bandages applied to the wounds. Additional compression to the source of bleeding can also be achieved for some penetrating injuries by Foley catheter insertion directly into the wound, initially described in bleeding penetrating neck injuries [18, 19]. Compression bandages impregnated or combined with topical haemostatics enhance bleeding control in the pre-hospital setting [20] (see also recommendation R22).

In mangled extremity injuries, penetrating or blast injuries, traumatic amputations and sometimes in more limited extremity injuries, the application of a tourniquet is necessary to achieve complete bleeding control [20–23]. The use of tourniquets has become the standard of care for severe external haemorrhage in military medicine and several publications report the effectiveness of tourniquets in this specific context in adults [22] and children [23]. In the civilian setting, several small studies and systematic reviews suggest reduced mortality with the use of pre-hospital tourniquets and a low risk of complications, although there is a lack of high-quality randomised controlled trials (RCTs) to support this practice [24, 25]. Tourniquets should be left in place until surgical control of bleeding is achieved; however, time to removal should be shortened as much as possible [20]. Improper or prolonged placement of a tourniquet can lead to complications such as nerve paralysis and limb ischaemia, but these effects are rare [25–27].

#### Ventilation

##### Recommendation 3

We recommend that endotracheal intubation or alternative airway management be performed without delay in the presence of airway obstruction, altered consciousness [Glasgow Coma Scale (GCS) ≤ 8], hypoventilation or hypoxaemia (Grade 1B).

We recommend the avoidance of hypoxaemia (Grade 1A).

We suggest the avoidance of hyperoxaemia, except in the presence of imminent exsanguination (Grade 2B).

We recommend normoventilation of trauma patients (Grade 1B).

We suggest hyperventilation as a life-saving measure in the presence of signs of cerebral herniation (Grade 2C).

#### Rationale

The fundamental objective of intubation is to ensure patency of the airways and facilitate adequate ventilation and oxygenation. There are well-defined situations in which intubation is mandatory, including in the presence of airway obstruction, altered consciousness (GCS ≤ 8), haemorrhagic shock, hypoventilation or hypoxaemia. To intubate the trachea, rapid sequence induction appears to be the best method [28]. Tracheal intubation of severely injured patients is a delicate procedure that involves risks and requires skill and proper training of the operator. A rather old study even reported increased mortality associated with pre-hospital intubation in patients with severe brain injury [29]. Hence, alternative methods for advanced airway management may find a place in patient management. However, a recent trial found that supraglottic placement of a supraglottic airway device was not superior to endotracheal intubation after cardiac arrest [30]. Fluid administration is usually required concurrently, as the introduction of positive intrathoracic pressure can induce a severe hypotension in hypovolaemic patients. Other questions remain, including which drugs can be recommended. Therefore, controversy remains about the appropriate use of tracheal intubation in patients following traumatic injury [31].

The negative effects of hypoxaemia are well known, particularly in patients with traumatic brain injury (TBI) [32], therefore, high oxygen concentrations are generally targeted during the initial management of these patients to ensure adequate oxygen delivery to ischaemic areas. Some studies and meta-analyses based on high-quality evidence [33] have suggested that prolonged hyperoxia (PaO_2_ well above the normal range) is associated with increased mortality [34, 35]. Extreme hyperoxia [PaO_2_ > 487 mmHg (> 65 kPa)] should therefore be avoided in patients with TBI [36]. The negative effects of hyperoxia are likely related to altered microcirculation associated with high PaO_2_ [37] and increased production of oxygen free radicals [38] and patients with severe brain injury may be at particular risk [36]. Therefore, although hyperoxia may increase the oxygen content and delivery in an extremely anaemic trauma patient and be associated with a benefit in this specific situation, hyperoxia should be returned to normoxia as soon as the haemoglobin (Hb) level returns to more acceptable levels [36].

Adequate ventilation is desirable, but there is a tendency for rescue personnel to hyperventilate patients during initial resuscitation. The effect of hyperventilation on bleeding and outcome in patients with severe trauma without TBI is not known. There are several potential mechanisms by which the adverse effects of hyperventilation and hypocapnia could be mediated, including increased vasoconstriction with decreased cerebral blood flow and impaired tissue perfusion. In the setting of absolute or relative hypovolaemia, an excessive rate of positive pressure ventilation may further compromise venous return and produce hypotension and even cardiovascular collapse [38, 39]. A target PaCO_2_ should be 5.0–5.5 kPa (35–40 mmHg).

The only situation in which hyperventilation-induced hypocapnia may be desirable is in the context of imminent cerebral herniation, wherein the decrease in cerebral blood flow produced by acute hypocapnia causes a decrease in intracranial pressure. This should be considered only for short periods of time until other measures are effective and in selected cases of imminent brain herniation. The presence of signs such as unilateral or bilateral pupillary dilation or decerebrate posturing are indicators for an extreme risk of imminent death or irreversible brain damage. Given the extreme risk of death if no measures are undertaken, the risk–benefit balance seems favourable; however, it is important to normalise PaCO_2_ as soon as feasible.

Ventilation with low tidal volume (around 6 mL/kg) is now recommended in all patients treated with mechanical ventilation, including during surgery.

#### Pre-hospital blood product use

##### Recommendation 4

No clear recommendation or suggestion in favour or against the use of pre-hospital blood products can be provided at this time.

The pre-hospital use of blood products is technically feasible; however, logistical hurdles and the scarcity of universal blood group donors, along with health economic challenges and financial burdens, remain subjects of ongoing investigation and debate. The best evidence available to date for pre-hospital plasma administration is derived from two pragmatic RCTs, PAMPer (Pre-hospital Air Medical Plasma; [40]) and COMBAT (Control of Major Bleeding after Trauma; [41]), which have yielded conflicting results. Secondary post hoc analyses have suggested greater benefits for patients who are coagulopathic, with blunt injury [42], a computed tomography (CT)-positive TBI [43] or with pre-hospital rescue times > 20 min [44]. A meta-analysis of both trials including 626 patients showed reduced 24 h mortality with pre-hospital plasma but no effect on 1-month mortality [45]. For pre-hospital packed red blood cell (pRBC) concentrates, single-centre observational or retrospective studies have suggested improvements in both haemodynamics and survival, but are restricted to the pre-hospital phase of care with overall reduced blood product consumption [46]. In a meta-analysis of matched trauma patients, the individual use of pRBC showed no difference in long-term mortality or 24 h mortality [47] and consistent evidence for beneficial effects with pre-hospital pRBC is still lacking [48].

The combined use of pre-hospital pRBC and plasma was assessed in a secondary analysis of PAMPer among 407 hypotensive trauma patients divided into four pre-hospital resuscitation groups: crystalloid only; pRBC; plasma; pRBC + plasma, with the greatest survival benefit in the latter group at 30 days [49]. Mortality was statistically lower per unit of pRBC and plasma. A meta-analysis on matched trauma patients that had received pre-hospital pRBC and plasma simultaneously showed a significant reduction in long-term mortality but no difference in 24 h mortality [47]. Pre-hospital use of freeze-dried plasma may have logistic benefits over thawed/frozen plasma and retrospective evidence has demonstrated feasibility, positive effects on coagulation [50], and when administered as bolus followed by pre-hospital pRBC, a capacity to reduce pRBC requirements [51]. The multicentre phase 3 RCT RePHILL (Resuscitation with pre-hospital blood products) trial compared pre-hospital two units of pRBC and LyoPlas each (*n* = 209) or up to 1 L 0.9% sodium chloride (*n* = 223) in adult trauma patients with haemorrhagic shock and hypotension and did not show a difference for the composite endpoint mortality and/or lactate clearance [52]. The trial was stopped at 432/490 patients due to the SARS-CoV-2 pandemic.

Due to conflicting data and the financial burden involved in the design and implementation of pre-hospital pRBC and plasma transfusion programmes that may or may not provide definitive evidence, no clear recommendation or suggestion in favour or against the use of pre-hospital blood products can be provided at this time. The decision to commit to routine pre-hospital use of blood products requires careful consideration by all stakeholders and must be adapted to local circumstances and settings [52].

### II. Diagnosis and monitoring of bleeding

#### Initial assessment

##### Recommendation 5

We recommend that the physician clinically assess the extent of traumatic haemorrhage using a combination of patient physiology, anatomical injury pattern, mechanism of injury and the patient response to initial resuscitation (Grade 1C).

We recommend that the shock index (SI) and/or pulse pressure (PP) be used to assess the degree of hypovolaemic shock and transfusion requirements (Grade 1C).

#### Rationale

The mnemonic Advanced Trauma Life Support (ATLS) ABCDE has been replaced by <C> ABCDE, with <C> referring to critical/catastrophic bleeding requiring rapid bleeding control and resuscitation with blood products, including massive transfusion (MT). The traditional ATLS classification system of hypovolaemic shock now includes physiological base excess and may serve as a rough estimate for blood loss and transfusion requirements, but is not without limitations (Table 2; [53, 54]). Numerous predictors of traumatic haemorrhage and scores/models have been introduced, but with an overall low quality/variable performance [area under the receiver operating characteristics (AUROC) 0.73–0.95] and a consistent lack of prospective validation; none are in widespread clinical use [55]. While some models aim to predict coagulopathy, others aim to evaluate the risk of MT. In a meta-analysis including 84 studies describing any predictor-outcome association, 47 included multivariate models and 26 were specifically designed for prediction [55]. A total of 35 distinct predictors were identified, of which systolic blood pressure, age, heart rate and mechanism of injury were most frequently investigated. Only 21 multivariate models met the recommended sample size threshold of 10 events per predictor, and seven predictors were examined in at least two models: mechanism of injury, systolic blood pressure, heart rate, haemoglobin, lactate and focused assessment with sonography in trauma (FAST) [55]. Information on the mechanism of injury is useful and a threshold of 6 m (20 ft) as a “critical falling height” has been associated with major injuries, including haemorrhage [56]. Trapped patients were more likely to have time-critical injuries with significant blood loss requiring intervention [57]. Further critical mechanisms include high-energy deceleration impact as well as low-velocity versus high-velocity gunshot injuries. The individual response to fluid challenge may be viewed critically in the context of low-volume resuscitation and “permissive hypotension”.

**Table 2:** American College of Surgeons Advanced Trauma Life Support (ATLS) classification of blood loss based on initial patient presentation. Signs and symptoms of haemorrhage by class

Parameter	Class I	Class II (mild)	Class III (moderate)	Class IV (severe)
Approximate blood loss	< 15%	15–30%	31–40%	> 40%
Heart rate	↔	↔/↑	↑	↑/↑↑
Blood pressure	↔	↔	↔/↓	↓
Pulse pressure	↔	↓	↓	↓
Respiratory rate	↔	↔	↔/↑	↑
Urine output	↔	↔	↓	↓↓
Glasgow coma scale score	↔	↔	↓	↓
Base deficit*	0 to − 2 mEq/L	− 2 to − 6 mEq/L	− 6 to − 10 mEq/L	− 10 mEq/L or less
Need for blood products	Monitor	Possible	Yes	Massive transfusion protocol

Table reprinted with permission from the American College of Surgeons [53]

Original data from Mutschler et al. [54]

*Base excess is the quantity of base (HCO_3_^−^, in mEq/L) that is above or below the normal range in the body. A negative number is called a base deficit and indicates metabolic acidosis

The SI is the ratio of heart rate to systolic blood pressure, typically ranging between 0.5 and 0.7 in healthy adults. A SI ≥ 0.9–1.0 was retrospectively associated with increased MT (25%), interventional radiology (6.2%) and operative intervention (14.7%) in bleeding trauma patients [58]. Further retrospective studies have used different cut-offs for MT; however, each study had thresholds between SI ≥ 0.8– ≥ 1.0 with AUROCs between 0.73 and 0.89 [59–62]. In a prospective data collection of 1402 trauma patients, SI ≥ 0.8 was more sensitive than SI ≥ 0.9 [59]. At cut-off 0.81, SI predicted MT with a sensitivity of 85%, specificity 64%, positive prediction 16% and negative prediction 98%, with correlation to other physiological and anatomical variables [63]; at cut-off 0.91 SI predicted MT with a sensitivity of 81% and specificity of 0.87 [62]. After adjusting for age/sex, injury severity score (ISS), GCS, SI was an independent predictor for mortality and blood transfusion (OR 3.57; 3.012–4.239; [63]). A SI ≥ 1 retrospectively outperformed the ABC score for MT [60] and clinical hypotension for significant injury and emergent operation [64]. Narrow PP (< 40/ < 30 mmHg) is an ATLS class II haemorrhage signal and was independently associated with transfusion, resuscitative thoracotomy and emergent surgery in a series of retrospective studies [65–67]. Multivariate analysis of observational data from 957 patients confirmed a narrowed PP (< 30 mmHg) to be significantly associated with MT (OR 3.74, 95% CI 1.8–7.7) and emergent surgery [68].

#### Immediate intervention

##### Recommendation 6

We recommend that patients with an obvious bleeding source and those presenting with haemorrhagic shock in extremis and a suspected source of bleeding undergo an immediate bleeding control procedure (Grade 1B).

#### Rationale

The patient who presents in severe haemorrhagic shock has already lost a large volume of blood. If bleeding continues in so-called agonal patients, death is an imminent risk if the source of bleeding is not rapidly controlled. In a study of 271 patients undergoing immediate laparotomy for gunshot wounds, data indicate that these wounds combined with signs of severe hypovolaemic shock require early surgical bleeding control [69]. The selection of patients with severe shock for direct operative room trauma intervention was also beneficial for outcomes when compared with an expected TRISS score [70]. Similar findings have been observed in a paediatric population [71]. Johnson et al. studied 16,113 trauma admissions, among which 628 patients sent for direct to operation room resuscitation could be retrieved. The best predictors for the need of rapid surgical intervention were penetrating truncal mechanism, significant anatomy or examination findings such as amputations and major physiological derangements, including pre-hospital CPR and profound shock with a systolic blood pressure of less than 90 mmHg [72]. The relationship between time and bleeding control or “door-to-embolisation time” has also been observed for complex pelvic fractures [73].

#### Further investigation

##### Recommendation 7

We recommend that patients with an unidentified source of bleeding, but without a need for immediate bleeding control, undergo immediate further investigation to determine the bleeding source (Grade 1C).

#### Rationale

Haemodynamically stable patients, or those who can be stabilised during initial resuscitation, with an unidentified bleeding source, but not in need of immediate bleeding control, should undergo further investigation to determine the source of bleeding. During the primary survey, aside from monitoring vital signs, imaging studies (ultrasonography and CT) and laboratory blood tests (blood gas and coagulation status) are recommended [53, 74].

In recent years, the accessibility of CT scanners has increased dramatically, replacing the need for conventional radiographic imaging [75]. The diagnostic accuracy, safety and effectiveness of these immediate measures are dependent on pre-hospital treatment by trained and experienced emergency personnel and short transportation times [76, 77]. The proximity of the CT scanner to the resuscitation room in the emergency department has been shown to have a significant positive effect on the probability of survival for the severely injured patient [78]. The trauma workflow, comprising immediate CT diagnosis and rapid bleeding control without patient transfer, as realised in the hybrid emergency room, may improve survival in severe trauma [74]. If a CT scanner is not available in the emergency department, the clinician must evaluate the potential risks and benefits of patient transfer to a CT room, assuring continuous monitoring and resuscitation. In a well-structured environment and by a well-organised trauma team, CT seems to be safe and justified even in severely injured haemodynamically unstable patients [79].

In a retrospective study between 2016 and 2019, 2694 consecutive patients were admitted to a level I trauma centre and a strict emergency room algorithm followed. Injuries were missed in seven patients (0.26%; one epidural bleeding and six abdominal hollow organ injuries; two died), which highlights the need for continuous clinical and instrument-based examinations after completion of the tertiary survey [80].

Catheter angiography should be considered in patients with blunt pelvic trauma found to have active arterial extravasation, regardless of bleed size or patient clinical or laboratory values [81], while contrast extravasation on CT, high volume pRBC transfusions and ISS ≥ 16 can assist in identifying pelvic fracture patients for angiography with more precision [82].

A retrospective analysis and systematic review of epidemiology, radiologic examinations, patterns of injuries, therapeutic measures, clinical courses and outcomes showed that visceral perfusion should be monitored clinically and radiologically and follow-up via magnetic resonance imaging or computed tomography angiography performed in order to rule out vascular complications after traumatic dissection of celiac artery [83].

#### Imaging

##### Recommendation 8

We suggest the use of pre-hospital ultrasonography (PHUS) for the detection of haemo-/pneumothorax, haemopericardium and/or free abdominal fluid in patients with thoracoabdominal injuries, if feasible without delaying transport (Grade 2B).

We recommend the use of point-of-care ultrasonography (POCUS), including FAST, in patients with thoracoabdominal injuries (Grade 1C).

We recommend early imaging using contrast-enhanced whole-body CT (WBCT) for the detection and identification of the type of injury and the potential source of bleeding (Grade 1B).

#### Rationale

The accuracy of PHUS was adequate with high sensitivity and specificity for pneumothorax, free intraabdominal fluid and haemoperitoneum in a systematic review of three retrospective and six prospective observational studies including 2889 trauma patients [84]. Five studies reported at least one change in management. A more recent systematic review of 16 studies including 3317 trauma patients confirmed the feasibility/potential of PHUS, with seven studies evaluating treatment and transport impact, but with large inconsistencies in protocols, variables and outcomes, precluding a meta-analysis of the data [85].

In-hospital, POCUS, with its best-known application FAST, remains central to the primary ATLS survey for the detection of haemorrhage in pleural, pericardial and peritoneal cavities, with high specificity but overall variable to low sensitivity. Summary estimates of sensitivity and specificity for detecting/excluding free fluids, organ/vascular or other injuries compared with reference imaging and/or operative/autopsy findings were 0.74 and 0.96 in a review and meta-analysis of 34 studies with 8635 patients and any type of blunt civilian injury [86]. There was substantial heterogeneity across studies and the reported accuracy of POCUS varied greatly, depending on study populations and body areas affected. In general, a negative POCUS in the context of abdominal trauma cannot exclude injury and must be verified in any case against a standard reference, for example, CT. In a secondary analysis of prospective data from 317 hypotensive patients (< 90 mmHg systolic blood pressure) of the Prospective, Observational, Multicentre, Major Trauma Transfusion (PROMMTT) RCT, 22 FAST-negative patients required laparotomy within 6 h of admission; thus, in hypotensive patients with haemorrhage, significant intraabdominal haemorrhage must be suspected [87]. From a series of small studies in mixed populations, it was concluded that POCUS may have a higher sensitivity in the context of chest and cardiac injuries [86, 88, 89]. The classic FAST protocol can be augmented with an additional transverse scan of the pubic symphysis (FAST-PLUS protocol), with a high CT correlation with unstable pelvic injury [90]. Rolling patients to the right may increase FAST sensitivity by converting false-negatives into true positive examinations [91].

Observational/retrospective studies/reviews have confirmed the benefits of WBCT for time savings, diagnostic accuracy, localisation of bleeding sources/prioritisation of injuries for further diagnostics/interventions and, in part, also for survival in bleeding trauma patients [75, 92]. In a multicentre study, CT identified retroperitoneal haematoma in 100% of cases [93]; in 425 patients with abrasion/ecchymosis seat belt sign, CT was 100% sensitive for intraabdominal injury [94]. To date, the REACT-2 trial remains the only prospective RCT to compare immediate WBCT versus conventional imaging/selective CT in severe injuries with compromised vital parameters and found no survival benefit associated with WBCT, neither between groups, nor for polytrauma or TBI [95]. A secondary analysis (*n* = 172) assessed mortality in patients requiring emergency bleeding control interventions and found an absolute risk reduction of 11.2% (95% CI 0.3–22.7%) with immediate WBCT as the primary diagnostic modality [96]. WBCT markedly reduces time spent in the emergency department [97] and a median 19 min from admission to CT was significantly associated with decreased mortality from exsanguination in a single-centre experience [98]. A revised set of 10 clinical criteria for immediate WBCT with a high-positive predictive value for severe injury based upon secondary analysis of REACT-2 data is shown in Table 3 [99]. Given the post hoc analysis in a subset of patients on which these data are based, these criteria may not apply to all patients, and a targeted approach may be warranted. As with POCUS, haemodynamic factors may affect the sensitivity of contrast-enhanced CT [100].

**Table 3:** Revised criteria for immediate whole-body computed tomography in trauma patients

Trauma patients with one of the following parameters at hospital arrival:
Systolic blood pressure < 100 mmHg
Estimated exterior blood loss ≥ 500 mL
Glasgow coma scale score ≤ 13 or abnormal pupillary reaction
AND/OR
Patients with a clinical suspicion of one of the following diagnoses:
Fractures of at least two long bones
Flail chest, open chest, or multiple rib fractures
Severe abdominal injury
Pelvic fracture
Unstable vertebral fractures/spinal cord compression
AND/OR
Patients with one of the following injury mechanisms:
Fall from a height (> 4 m/> 13 ft)
Wedged or trapped chest/abdomen

Criteria reprinted with permission [99]

#### Haemoglobin

##### Recommendation 9

We recommend the use of repeated Hb and/or Hct measurements as a laboratory marker for bleeding, as an initial value in the normal range may mask early-phase bleeding (Grade 1B).

#### Rationale

Hb or haematocrit (Hct) assays are key parts of the basic diagnostic work-up for bleeding trauma patients. Both parameters are used interchangeably in clinical practice and here we refer to both parameters according to the parameter described by the literature cited. Recently, non-invasive Hb monitoring has also been tested and showed high precision compared with laboratory measurements [101].

The diagnostic value of the Hb or Hct for quantifying and/or detecting blood loss in trauma patients with severe injury and for occult bleeding sources has been a topic of debate. A major limitation of the diagnostic value is the confounding influence of resuscitation fluids and physiological shift of interstitial fluid into the vascular compartment [102]. Low initial Hct or Hb levels in trauma patients closely correlate with haemorrhagic shock [103, 104]. In a retrospective analysis of 1492 consecutive trauma patients, Thorson et al. found that the initial Hct was associated more closely with the need for transfusion than other parameters such as heart rate, blood pressure or acidaemia [105]. Serial measurements increase the sensitivity of these parameters to detect blood loss in patients with severe injury [106, 107]. Holstein and co-workers showed that a Hb level below 80 g/L in patients with pelvic trauma was associated with non-survival [108] and there was also a close correlation between Hb and fibrinogen levels [109]. Because initial Hb values close to the normal range may mask early-phase serious bleeding [110], repeated measurement is prudent.

In summary, initial Hct and Hb value changes over time represent simple and reliable bedside parameters with which to detect blood loss, despite several limitations.

#### Blood lactate and base deficit

##### Recommendation 10

We recommend blood lactate as a sensitive test to estimate and monitor the extent of bleeding and tissue hypoperfusion; in the absence of lactate measurements, base deficit may represent a suitable alternative (Grade 1B).

#### Rationale

In hypovolemic shock, the amount of lactate is primarily produced by anaerobic glycolysis and is therefore an indirect marker of cellular hypoxia. Altered liver perfusion can also prolong the lactate clearance. Blood lactate has been used as a diagnostic and prognostic marker of haemorrhagic shock since the 1960s [111] and is considered to reflect the severity of haemorrhagic shock. Studies have shown the value of serial lactate measurements for predicting survival in shock [112] and also provide an early and objective evaluation of patient response to therapy [113]. The determination of lactate may be particularly important in penetrating trauma, where vital signs, such as blood pressure, heart rate and respiratory rate, do not reliably reflect the severity of injury [112]. The reliability of lactate determination may be lower when traumatic injury is associated with alcohol consumption [114].

The initial base deficit, obtained either from arterial or peripheral venous blood is also a potent independent predictor of mortality in patients with traumatic haemorrhagic shock [115], both in adult and paediatric patients [116]. Base deficit values derived from arterial blood gas analysis provide an indirect estimation of global tissue acidosis due to impaired perfusion, provided there is no other cause of metabolic acidosis such as renal failure or hyperchloremia. Although both blood lactate levels and base deficits are well correlated with shock and resuscitation, these two variables do not strictly correlate with each other in severely injured patients and lactate levels more specifically reflect the degree of tissue hypoperfusion [117, 118].

#### Coagulation monitoring

##### Recommendation 11

We recommend the early and repeated monitoring of haemostasis, using either a traditional laboratory determination such as prothrombin time (PT)/international normalised ratio (INR), Clauss fibrinogen level and platelet count and/or point-of-care (POC) PT/INR and/or a viscoelastic method (Grade 1C).

#### Rationale

It is generally accepted that traumatic coagulopathy is defined using the prothrombin time ratio (PTr); where a PTr > 1.2 is the threshold for detecting traumatic coagulopathy and PTr > 1.5 is indicative of severe coagulopathy. Thresholds for other conventional clotting tests (CCTs) are less well-established and no consensus values defining traumatic coagulopathy have been agreed, despite low Clauss fibrinogen (< 1.3 g/L) and markers of fibrinolysis, such as elevated D-dimers, being commonly found in bleeding patients and associated with increased mortality [119, 120]. In contrast, platelet counts tend to fall late during trauma haemorrhage and poorly reflect the platelet dysfunction found after injury.

POC PT testing has obvious attractions as a diagnostic entity. Adding to the published data is a retrospective study of 522 patients comparing POC-PTr tests to laboratory PTr [121]. The authors reported good reliability and accuracy for POC-PTr when values were < 2.0, but, like previous studies [122, 123], precision fell as PTr rose. Despite these limitations, the authors reported that thresholds of POC-PTr of 1.2 and 1.4 could be used to detect moderate and severe traumatic coagulopathy, respectively [121].

Viscoelastic measures (VEM) are commonly used to detect traumatic coagulopathy. Up to now, VEM-guided transfusion algorithms have been developed at single centres. A recent study reported the development of three pragmatic algorithms detecting key coagulation defects (PTr > 1.2, fibrinogen < 2 g/L, platelets < 100 × 10^9^/L) and defining simple transfusion thresholds, using data prospectively collected from six European sites [*n* = 968 thromboelastography (TEG); 2019 rotational thromboelastometry (ROTEM); 2287 CCT] [124]. A multicentre RCT (iTACTIC; NCT02593877) subsequently tested these algorithms in 690 adults, comparing empiric transfusion therapy guided by either: CCT or VEM (ROTEM/TEG) [125]. No difference in the primary outcome (alive and free from MT at 24 h) was reported VEM 67%, CCT 64%, OR 1.15 (95% CI 0.76–1.73). Notably, in a pre-specified subgroup of 74 TBI patients, a significant difference was seen in 28-day mortality: VEM 44%, CCT 74%, OR 0.28 (95% CI 0.10–0.74). An important limitation to this study was the small number of included coagulopathic patients (29%)—the group deemed to have the most to gain by the intervention [125].

TBI is an area of increasing focus with VEM. In a retrospective study, a distinct r-TEG pattern was reported for TBI, characterised by prolonged activated clotting time (> 128 s), reduced *α*-angle (< 65°), low functional fibrinogen levels (< 365 mg/dL), normal maximum amplitude and no increased fibrinolysis (Ly30 1.2%) [126]. Added to this, a systematic review of 31 studies concluded that TEG can readily detect the coagulopathy of TBI and indeed a variety of coagulopathy subtypes can be described according to TBI severity [127]. Notably, increased % inhibition of the TEG-platelet mapping cartridges for arachidonic acid (AA) and adenosine diphosphate (ADP) was consistently reported in the TBI cohorts [127]. However, a second systematic review was more reserved in its findings, concluding that more data are required before it is possible to state that VEM assays are useful for the detection of TBI-related coagulopathy and its subsequent treatment [128].

One major concern around the use of VEM for detecting coagulopathy/guiding therapy is the inter-and intra-variability of results between hospitals and operators. Cartridge-based ROTEM and TEG devices have been brought to market in part to address this. A multicentre study across 12 US trauma centres was conducted comparing reliability of the TEG 6S with TEG 5000 [129]. The two devices gave results that were well correlated and importantly, a strong within-device reproducibility for the TEG 6S machine [129].

#### Platelet function monitoring

##### Recommendation 12

We recommend that the routine use of POC platelet function devices for platelet function monitoring in trauma patients on antiplatelet therapy or with suspected platelet dysfunction be avoided (Grade 1C).

#### Rationale

Current platelet function POC devices measure different parameters of platelet activation and have different levels of sensitivity, therefore, they are not interchangeable in the assessment of platelet reactivity. Moreover, results may be of limited value if platelet counts are low. Different POC platelet function tests (PFTs) were used in several observational studies to detect antiplatelet agents (APAs) and induced platelet inhibition in trauma patients, with mixed results [130–133]. In a small observational study that prospectively compared Multiplate^®^, TEG^®^-PM^®^ and VerifyNow^®^ in populations treated or not with APAs, the three devices detected APA use with an area under the curve (AUC) of 0.90, 0.77 and 0.90, respectively [134]. With Multiplate^®^ < 40 U as a reference, TEG^®^-PM^®^ and VerifyNow^®^ detected platelet dysfunction with an AUROC of 0.78 and 0.89, respectively.

The utility of POC-PFTs in the detection or exclusion of pre-injury APA treatment is limited, as several observational studies found that trauma patients, especially those with TBI, had therapeutic assay results or values below the reference interval, independent of APA intake history [135–138]. In a prospective observational study that included 824 adult trauma patients with suspected pre-injury APA treatment and who were tested using thromboelastography with platelet mapping (TEG-PM), AA inhibition accurately detected pre-injury APA and aspirin use (AUROC, 0.89 and 0.84, respectively); however, ADP inhibition performed poorly (AUROC, 0.58). Neither AA nor ADP inhibition was able to discern specific APA regimens or entirely rule out APA use [132].

As diagnostic cut-offs for pathologic platelet dysfunction after traumatic injury have not been established, distinguishing pharmacologic from trauma-induced platelet receptor hypofunction is not easy. Moreover, the in vivo platelet response to the individual agonists utilised in POC-PFTs to induce activation and aggregation may not be adequate for detecting traumatic platelet dysfunction.

Consequently, the role of POC-PFTs in predicting outcome or stratifying trauma patients at a higher risk of bleeding who may subsequently benefit from platelet transfusion has not been established. Several observational studies using different POC-PFTs found conflicting results regarding the severity of trauma and prognostic information that various tests may provide [134, 136–138]. In a prospective study including 221 patients with traumatic intracranial haemorrhage (TICH), patients with nonresponsive platelets had similar in-hospital mortality [3 (3.0%) vs. 6 (6.3%), *p* = 0.324], TICH progression [26 (27.1%) vs. 24 (26.1%), *p* = 0.877], intensive care unit admission rates [34 (34.3%) vs. 38 (40.0%), *p* = 0.415] and length of stay [3 (interquartile range, 2–8) vs. 3.2 (interquartile range, 2–7) days, *p* = 0.818] as those with responsive platelets [136]. In contrast, a systematic review including 16 studies on adult patients with TBI (isolated or polytrauma) indicates that TEG-PM assays are associated with mortality and bleeding complications, but points to the low quality of current evidence in this population [127].

The role of POC-PFTs in guiding haemostatic therapy is also uncertain. While there is some evidence that platelet transfusion can correct platelet dysfunction in TBI [131, 138] and limit the overall administration of blood products [139, 140], other studies have failed to confirm the improvement of platelet function [130, 137] or outcome [131]. In an observational study that retrospectively included 157 patients with TICH, a platelet reactivity test and guided platelet transfusion strategy were not associated with a difference in intracranial haemorrhage (ICH) worsening [133]. It also seems that platelet transfusion may enhance platelet function via AA receptor-mediated pathways but has little impact on ADP receptor-mediated pathways [135].

In summary, there is weak scientific evidence of a clinically meaningful influence of POC-PFTs in trauma patients and an urgent need for future studies to elucidate their potential clinical benefits.

### III. Tissue oxygenation, volume, fluids and temperature

#### Volume replacement and target blood pressure

##### Recommendation 13

In the initial phase following trauma, we recommend the use of a restricted volume replacement strategy with a target systolic blood pressure of 80–90 mmHg (mean arterial pressure 50–60 mmHg) until major bleeding has been stopped without clinical evidence of brain injury (Grade 1B).

In patients with severe TBI (GCS ≤ 8), we recommend that a mean arterial pressure ≥ 80 mmHg be maintained (Grade 1C).

#### Rationale

The initial treatment of trauma-induced hypotension uses the concept of a restricted volume replacement and permissive hypotension. This strategy was mainly triggered by a RCT published in the 1990s demonstrating increased survival in penetrating trauma [141]. In the meantime, this strategy is replacing conventional aggressive fluid resuscitation. A recent meta-analysis of RCTs analysed mortality in trauma patients without TBI receiving either traditional aggressive fluid resuscitation or following a restricted volume replacement and permissive hypotension concept found a decrease in mortality when the latter concept was used [142, 143].

This concept is supported by several meta-analyses of retrospective studies alone [144] as well as combined prospective and retrospective studies showing reduced mortality in comparison to traditional aggressive volume replacement targeting normotension [49, 145, 146]. Several retrospective studies demonstrated that aggressive resuscitation techniques, often initiated in the pre-hospital setting, not only increased mortality, but also more often resulted in damage control laparotomy, coagulopathy, multiorgan failure, nosocomial infections, a need for transfusions and prolonged intensive care unit (ICU) and length of hospital stays [147–149]. A recently published retrospective analysis of the effect of aggressive volume resuscitation confirmed the potential harm associated with this strategy in comparison to a restrictive volume strategy in a paediatric trauma population [150].

It should be noted that the concept of permissive hypotension and restrictive volume resuscitation is contraindicated in patients with TBI and spinal injuries. This is because an adequate perfusion pressure is crucial to ensure tissue oxygenation of the injured central nervous system. However, it remains unclear how to attain the best balance between volume resuscitation and vasopressor administration in order to achieve an adequate perfusion pressure. Therefore, rapid bleeding control is of particular importance in these patients. In addition, the concept of permissive hypotension should be carefully considered in elderly patient [151] and may be contraindicated if the patient suffers from chronic arterial hypertension.

In conclusion, a damage control resuscitation strategy using a concept of restricted fluid replacement that aims to achieve a reduced systolic blood pressure of 80–90 mmHg in patients without TBI and/or spinal injury is supported by the literature. However, the currently available data should be interpreted with caution; reported RCTs are limited by a low number of patients included and poor-to-moderate quality. The retrospective data suffer from inherent limitations as well as methodological weaknesses such as a high risk of selection bias and clinical heterogeneity. Therefore, further confirmation in adequately powered prospective RCTs is needed.

#### Vasopressors and inotropic agents

##### Recommendation 14

If a restricted volume replacement strategy does not achieve the target blood pressure, we recommend the administration of noradrenaline in addition to fluids to maintain target arterial pressure (Grade 1C).

We recommend infusion of dobutamine in the presence of myocardial dysfunction (Grade 1C).

#### Rationale

Several retrospective studies describe an increase in mortality [152–155] or no benefit [156] when noradrenaline vasopressors are used in the trauma setting. Another study found no independent association between mortality and vasopressor use in trauma patients, except for the use of epinephrine [157]. A systematic review of early vasopressor use in trauma published in 2017 was unable to conclude whether vasopressors cause more harm or benefit when administered to patients with severe hypotension [158]. However, all previous studies, including the recent systematic review, comprise studies of very low quality and a high risk of bias; most notably, patients receiving vasopressors were systematically more severely ill than those not receiving vasopressors. The threshold for hypotension in most studies was defined as a systolic blood pressure of < 85 or 90 mmHg, without stating a precise threshold at which vasopressors were used. However, a systolic blood pressure of 80–90 mmHg in most patients does not represent life-threatening hypotension. In these cases, the use of vasopressors raises concerns about altering organ perfusion by potentiating vasoconstriction and consequently causing a further reduction of organ perfusion, which may inflict harm to the patient.

Therefore, in the early stages of resuscitation the present evidence supports a strategy of restricted volume replacement and permissive hypotension until the bleeding is controlled without the use of a vasopressor if a target systolic blood pressure of 80–90 mmHg can be achieved. However, if these measures fail to achieve the target blood pressure and if severe haemorrhage-induced hypotension with a systolic blood pressure < 80 mmHg occurs, transient noradrenaline is recommended to maintain life and tissue perfusion.

Nevertheless, it is well known that the pathophysiology of acute blood loss consists of two phases, an initial vasoconstriction, a sympathoexcitatory and later a vasodilatory, sympathoinhibitory phase, which during haemorrhagic shock may cause a further reduction in vascular tone in the severely bleeding trauma patient [159]. Therefore, in order to achieve an appropriate balance between intravascular volume and vascular tone, it may be beneficial to counteract vasodilation in the presence of haemorrhage [159]. Following the hypothesis that severe haemorrhagic shock is associated with a state of arginine vasopressin deficiency, Sims et al. performed a RCT in 100 trauma patients with haemorrhagic shock to assess the effect of supplementation of this hormone [160]. This small but well-designed study showed that low-dose arginine vasopressin (bolus of 4 IU followed by 0.04 IU/min) decreases blood product requirements. These findings are in line with an earlier double-blind randomised trial that assessed the safety and efficacy of adding vasopressin to resuscitative fluid [161]. Patients were administered fluid alone or fluid plus vasopressin (bolus 4 IU) and i.v. infusion of vasopressin (0.04 IU/min) for 5 h. The fluid plus vasopressin group needed a significantly lower total resuscitation fluid volume over 5 days than the control group (*p* = 0.04). The rates of adverse events, organ dysfunction and 30-day mortality were similar. In summary, additional research is needed to determine whether including low-dose arginine vasopressin improves morbidity or mortality.

Cardiac dysfunction could be altered in the trauma patient following cardiac contusion, pericardial effusion or secondary to brain injury with intracranial hypertension. The presence of myocardial dysfunction requires treatment with an inotropic agent such as dobutamine or epinephrine. In the absence of an evaluation of cardiac function or cardiac output monitoring, as is often the case in the early phase of haemorrhagic shock management, cardiac dysfunction must be suspected if there is a poor response to fluid expansion and norepinephrine.

#### Type of fluid

##### Recommendation 15

We recommend that fluid therapy using a 0.9% sodium chloride or balanced crystalloid solution be initiated in the hypotensive bleeding trauma patient (Grade 1B).

We recommend that hypotonic solutions such as Ringer’s lactate be avoided in patients with severe head trauma (Grade 1B).

We recommend that the use of colloids be restricted due to the adverse effects on haemostasis (Grade 1C).

#### Rationale

Whereas the use of crystalloids is widely accepted as part of an initial restrictive fluid replacement strategy in the bleeding trauma patient, the type of crystalloid is still under discussion. In most trauma studies 0.9% sodium chloride was used as the crystalloid solution. However, there exist concerns that saline as the main i.v. fluid results in harm to patients, such as hyperchloraemic acidosis or increased incidence of kidney injury, which may reduce survival. In contrast to 0.9% sodium chloride, balanced electrolyte solutions comprise physiological or near-physiological concentrations of chloride and may therefore be advantageous [162]. Whereas a large RCT including 15,802 critically ill patients comparing balanced crystalloids versus 0.9% sodium chloride showed a lower rate of the composite outcome “death from any cause, new renal-replacement therapy or persistent renal dysfunction” when balanced crystalloids were used [163], one recently published RCT [164] and two meta-analyses comparing the effect of balanced crystalloids versus 0.9% saline for resuscitation of critically ill adults did not show a difference in mortality, acute kidney failure or length of hospital stay [165, 166]. Nevertheless, knowing that further studies in trauma patients are warranted to clarify which crystalloid solution is the best for initial trauma management, the authors of this guideline favour a balanced electrolyte solution as the initial crystalloid solution in trauma patients. However, if a 0.9% sodium chloride solution is used, it should be limited to a maximum of 1–1.5 L. Saline solutions should not be used in severe acidosis, especially when associated with hyperchloremia.

Hypotonic solutions, such as Ringer’s lactate or hypotonic albumin should be avoided in patients with TBI in order to minimise a fluid shift into the damaged cerebral tissue. A secondary analysis from the PROMMTT study revealed that Ringer’s lactate solutions were associated with higher adjusted mortality compared with normal saline [167]. Hypertonic solutions, on the other hand, did not influence survival or 6-month neurological outcome in patients with and without TBI [168–170].

Colloid solutions have been used more effectively to restore intravascular volume, as would be expected from basic physiologic concepts of fluid exchange across the vasculature. A review of RCTs indicated that colloid solutions can result in lower fluid requirements than crystalloids in all types of patients, including trauma victims, with a ratio of 1.5/1 [171]. However, it is still unclear whether colloids really have a beneficial effect on morbidity or mortality. Two recently published meta-analyses comparing colloids such as starch solutions, gelatine and albumin with crystalloids failed to demonstrate a benefit on survival of colloids in surgical patients [172, 173]. Neither meta-analysis revealed an increase in renal failure or replacement therapy when colloids were administered in surgical patients in need of hypovolemic resuscitation. However, the most recent meta-analysis by Chappell et al. demonstrated improved haemodynamic stability, a reduced need for vasopressors and reduced the length of hospital stay by 9 h in surgical patients treated with starches in addition to crystalloids [174]. The present data in trauma resuscitation do not allow a recommendation as to which of the different colloids is best for the initial management of the bleeding trauma patient. Moreover, neither the timepoint of fluid resuscitation nor the duration and dose of fluid resuscitation have been analysed to date.

In conclusion, for the initial phase of traumatic haemorrhagic shock, a restrictive volume strategy using crystalloid solutions is generally accepted. The main rationale for the primary use of crystalloids is that coagulation and platelet function are impaired by all hydroxyethyl starch and gelatine solutions. These negative effects on coagulation might be partially improved using fibrinogen concentrate, depending on the type of colloids and concentration of fibrinogen concentrate being used [175, 176]. However, if bleeding is excessive and if crystalloids in combination with vasopressors are unable to support maintenance of basic tissue perfusion, colloid infusions represent a further option to restore perfusion.

#### Erythrocytes

##### Recommendation 16

If erythrocyte transfusion is necessary, we recommend a target haemoglobin of 70–90 g/L (Grade 1C).

#### Rationale

pRBC transfusion improves volume status and restores arterial oxygen transport during haemorrhagic shock resuscitation. Although pRBC transfusion is extensively used in trauma patients to replace blood loss until bleeding is controlled, few studies have compared different haemoglobin levels relative to pRBC transfusion. In a recent Cochrane database analysis that assessed haemoglobin thresholds to guide pRBC transfusion, there was no evidence of harm associated with targeting a restrictive threshold between 70 and 80 g/L as compared to a threshold greater than 90–100 g/L in 48 trials involving 21,433 patients [177]. However, high-quality data were only available in cardiac, orthopaedic surgery and critical care patients and no study on acute bleeding in trauma patients was included in the analysis. In a small-sample-size before–after study (*n* = 131 patients) in which the transfusion threshold was decreased from 70 to 65 g/L in a trauma centre, no difference was reported in hospital length of stay or organ failure [178]. Above all, physicians should keep in mind that haemorrhagic shock may be a rapidly evolving situation in which anticipation of transfusion is essential to prevent an excessive decrease in arterial oxygen transport and the decision to transfuse should not be based on haemoglobin levels alone.

Brain-injured patients may be especially at risk of ischaemia during acute anaemia. For this reason, different transfusion thresholds may apply for these patients. In a recent meta-analysis compiling 4 studies (3 randomised controlled trials and one retrospective study) in TBI patients, a haemoglobin threshold of 70 g/L was associated with a better neurological outcome than a haemoglobin threshold of 100 g/L [179]. However, 55% of patients included in the meta-analysis were from a retrospective study exposing to a high risk of bias since the amount of packed pRBC administered may reflect patient severity rather than a threshold-guided transfusion. Neurologic outcome data were similar in the retrospective study [180] and the largest prospective randomised study [181], both favouring a restrictive transfusion trigger of ≤ 70 g/L. Interestingly, progressive haemorrhagic injury was also less frequent in patients randomised to a restrictive transfusion threshold (Hb < 70 g/L) [182]. In a feasibility study, Gobatto et al. randomised 44 moderate or severe TBI patients in 2 ICUs in Brazil to a restrictive (< 70 g/L) or a liberal (100 g/L) transfusion strategy [183]. The restrictive strategy led to a haemoglobin level of 84 ± 10 g/L versus 93 ± 13 g/L and hospital mortality was lower in the liberal transfusion group (7/23 vs. 1/21). Neurological outcome at 6 months tended to be better using the liberal strategy. This is in contrast to the much larger prospective randomised study by Robertson et al. with 200 patients showing similar mortality but a more favourable neurologic outcome in the restrictive haemoglobin transfusion (Hb < 70 g/L) group [181, 182]. In the meantime, following the haemorrhagic phase, an optimal transfusion threshold may be individualised according to brain multimodal monitoring in TBI patients.

#### Cell salvage

##### Recommendation 17

We suggest that cell salvage be considered in the presence of severe bleeding from an abdominal, pelvic or thoracic cavity (Grade 2B).

#### Rationale

The use of intraoperative cell salvage (ICS) during traumatic haemorrhage is not widespread and the evidence is limited [184]. Reinfusion of autologous blood can become a potential driver for worsening impaired coagulation, endothelial disbalance and immunomodulation. Bleeding may originate from multiple injuries and large processed volumes may exacerbate haemodilution. When haemostatic measures involve damage control by thoracotomy or laparotomy, a physical cavity, preferably uncontaminated, may be available from which to retrieve blood. Washing and optional double-suction can lead to removal of contaminants and microorganisms [185]. Salvaged blood can be passed through individual or double filters or with leukoreduction capacity. The immunological benefit of salvaged blood is not well defined; however, preliminary experimental studies suggest that the procedure may be advantageous [186].

To date, the only randomised controlled trial in trauma patients, who underwent ICS during 44 laparotomies for penetrating injuries, showed a decrease of 4.7 allogenic pRBC units within the first 24 h without increasing postoperative infection rates and no significant difference in survival [187]. Several retrospective studies also demonstrated efficacy in reducing allogeneic transfusion, but no difference in mortality. One cohort compared the use of ICS in 47 trauma patients undergoing emergency surgery (83% laparotomies) with 47 serving as the control group. The use of ICS accounted for 40–45% of transfusion requirements and halved the number of pRBC units and fresh frozen plasma (FFP) [188]. A feasibility study was conducted in 130 patients with combat-related injuries, with 27 receiving MT (defined as 10 pRBC in 12 h), among whom ICS was used in 17 cases. Autologous blood accounted for only 7.6% of blood products transfused. The best ratio of recovered to required pRBC mass was 39%, in patients undergoing laparotomy or thoracotomy after a gunshot wound [189]. Another review of 179 patients with penetrating and blunt abdominal trauma compared one group receiving only allogeneic blood (*n* = 108) to another receiving additional recovered blood (*n* = 71). Bleeding was significantly higher in the ICS group and the reinfused volume doubled. A logistic regression revealed that ISS > 25, systolic blood pressure < 90 mmHg and estimated blood loss > 2000 mL predicted mortality [190].

In acute unstable haemorrhagic pelvic trauma, ICS may be indicated when management involves an anterior approach and/or an open reduction internal fixation through an infraumbilical laparotomy or with preperitoneal pelvic packing (PPP) [191]. Observational studies in deferred osteosynthesis of complex fractures of the acetabulum concluded that greater fracture complexity is associated with more bleeding (anterior approach as a risk factor [192]) and the use of ICS more cost-effective [193], although in other evaluations no differences in allogeneic transfusion were found [194].

Blood from the thoracic cavity could be recovered and re-transfused from chest drains after haemothorax or after thoracotomy. In a multi-institutional retrospective study, 272 trauma patients were allocated to two groups based on transfusion of salvaged blood from the haemothorax. There was no significant difference in in-hospital complications, mortality or 24 h post-admission coagulation. Patients who had received autologous blood had lower requirements for allogeneic blood and platelet concentrates and the cost of transfusions was significantly lower [195].

There is no evidence that emergency autologous transfusion in trauma worsens clinical outcomes; however, the lack of quality randomised trials precludes a general recommendation. ICS could offer advantages for patients who refuse transfusion and in a resource-constrained environment, potentially including pre-hospital blood salvage, when transport and access to blood products are challenging [196].

#### Temperature management

##### Recommendation 18

We recommend early application of measures to reduce heat loss and warm the hypothermic patient to achieve and maintain normothermia (Grade 1C).

#### Rationale

Hypothermia in trauma patients has been consistently shown to increase mortality [197, 198] and blood product transfusions [198]. The effects of hypothermia include altered platelet function, impaired coagulation factor function (a 1 °C drop in temperature is associated with a 10% drop in function), enzyme inhibition and fibrinolysis. Often, the coagulation effects can only be detected using lab values when coagulation tests (PT and activated partial thromboplastin time [APTT]) are performed at the low temperatures present in patients with hypothermia, but not when assessed at 37 °C, as is routine practice for such laboratory tests. Hypothermia in severely affected trauma patients with a core body temperature < 35 °C is often associated with acidosis, hypotension and coagulopathy and is one of the key factors of so-called trauma-induced coagulopathy [199].

Hypothermia in trauma patients not only causes a higher morbidity and mortality, but also leads to higher blood loss and transfusion requirements [198]. This has been shown in a retrospective study of 604 trauma patients who required MT [200]. The authors performed a logistic regression analysis, which demonstrated that a temperature lower than 34 °C was associated with a greater independent risk of mortality greater than 80% after controlling for differences in shock, coagulopathy, injury severity and transfusion requirements. A further study performed a secondary analysis using 10 years of data from the Pennsylvania Trauma Outcome Study (PTOS), which showed in more than 11,033 patients with severe TBI that spontaneous hypothermia at hospital admission was associated with a significant increase in the risk of mortality [201]. In addition, induced hypothermia in TBI with temperatures between 32 and 35 °C for at least 48 h was associated with either no improved outcome [202–204] or higher mortality in a general population with TBI [205, 206]. Nevertheless, one meta-analysis showed that hypothermia in patients with increased intracranial pressure is beneficial, if used therapeutically rather than prophylactically [206].

In order to reduce the risk of hypothermia and of hypothermia-induced coagulopathy wet clothing should be removed, additional heat loss should be avoided and the ambient temperature should be increased. Forced air warming, warm fluid therapy and, in extreme cases, extracorporeal re-warming devices are further helpful measures. Another option might be the use of a hypothermia prevention and management kit, which is a low-cost, lightweight, low-volume commercial product that sustains 10 h of continuous dry heat. Although this kit was designed to prevent hypothermia during tactical casualty evacuation, its application is also feasible in the civilian sector for active re-warming of trauma patients [207].

Because coagulopathy in trauma increases mortality, normothermia with core temperatures between 36 and 37 °C should be targeted to create optimal pre-conditions for coagulation.

### IV. Rapid control of bleeding

#### Damage control surgery

##### Recommendation 19

We recommend damage control surgery in the severely injured patient presenting with haemorrhagic shock, signs of ongoing bleeding, coagulopathy and/or combined abdominal vascular and pancreatic injuries (Grade 1B).

Other factors that should trigger a damage control approach are hypothermia, acidosis, inaccessible major anatomic injury, a need for time-consuming procedures (Grade 1C).

We recommend primary definitive surgical management in the absence of any of the factors above (Grade 1C).

#### Rationale

The severely injured patient with continuing bleeding or deep haemorrhagic shock generally has a poor chance of survival. Without early control of bleeding and proper resuscitation, these patients exhaust their physiological reserves, with resulting profound acidosis, hypothermia and coagulopathy, also known as the “lethal triad”.

In 1983, Stone et al. described the techniques of abbreviated laparotomy [208] and ten years later Rotondo et al. defined the abbreviated laparotomy in three different stages (immediate laparotomy for control of bleeding and contamination, temporary closure of the abdomen for further resuscitation in the ICU before definitive repair) and coined it “damage control” (DC) [209]. The concept became widely accepted despite the lack of prospective randomised studies and DC techniques were described for other injuries outside the abdomen [210]. In addition, DC resuscitation became an entity and an essential adjunct to the surgical DC in achieving coagulation and reducing secondary complications [210, 211]. The situation for the severely traumatised patient in shock therefore changed and the indications for DC surgery required clarification, especially as DC surgery side effects became better known. In a systematic review of DC surgery in civilian trauma patients, several indications were identified, but few showed evidence of validity or were associated with better outcomes when DC was performed compared to definitive repair. The study concluded that DC surgery should be used only when definitive surgery cannot be performed [212]. The application of a DC surgical approach should therefore be limited to patients in which the “lethal triad” of physiological parameters is present and definitive surgery not feasible.

#### Pelvic ring closure and stabilisation

##### Recommendation 20

We recommend the adjunct use of a pelvic binder in the pre-hospital setting to limit life-threatening bleeding in the presence of a suspected pelvic fracture (Grade 1C).

We recommend that patients with pelvic ring disruption in haemorrhagic shock undergo pelvic ring closure and stabilisation as early as possible (Grade 1B).

#### Embolisation, packing, surgery and resuscitative endovascular balloon occlusion of the aorta (REBOA)

##### Recommendation 21

We recommend temporary extra-peritoneal packing when bleeding is ongoing and/or when angioembolisation cannot be achieved in a timely manner. Extra-peritoneal packing can be combined with open abdominal surgery when necessary (Grade 1C).

We suggest that REBOA be considered in patients with noncompressible life-threatening traumatic haemorrhage to bridge the gap between haemodynamic collapse and haemorrhage control (Grade 2C).

#### Rationale

External emergency stabilisation of unstable pelvic fractures reduces haemorrhage associated with bleeding pelvic fractures in most situations [213]. The use of a non-invasive pelvic binder, invasive external fixation or C-clamp depends on the individual injury pattern according to the principles of damage control orthopaedics [214–216].

Kim et al. reported that among 148 patients with pelvic fractures using the OTA/AO fracture classification 58.8% had type A, 34.5% type B and 6.7% type C fractures. Arterial bleeding seen on CT angiography was observed in 18.9%. Independent risk factors for bleeding included type B and C fractures, body temperature < 36 °C and blood lactate > 3.4 mmol/L [217].

Despite the use of a multidisciplinary treatment approach, the mortality rate associated with haemodynamic instability due to severe pelvic fracture remains 30% [218, 219]. Ongoing bleeding after external emergency stabilisation can be managed using either temporary retroperitoneal/PPP combined with laparotomy when needed and/or angioembolisation (AE) [220–222]. The critical decision to transfer a patient to the OR versus interventional radiology suite can be managed using intraoperative AE with C-arm digital subtraction angiography [221]. There was no significant difference in mortality observed between AE and PPP in patients with traumatic pelvic haemorrhage [223].

In selected patients, REBOA may serve as a bridge between haemodynamic collapse and definitive bleeding control. REBOA may also be used as an adjunct to PPP to stem temporary bleeding [224, 225]. The available data suggest that REBOA can temporarily improve haemodynamics [226, 227]. The evidence demonstrating that REBOA improves survival is conflicting [228–230] and REBOA is associated with potentially significant complications [224, 230]. A systematic review and meta-analysis concluded that no valid conclusions on the superiority of REBOA can be drawn compared with resuscitative thoracotomy or non-REBOA treatment in uncontrolled haemorrhagic shock [228]. Quality evidence to support the clinical use of REBOA is lacking [230]. Further studies should therefore be performed within specific training programmes and experimental settings.

#### Local haemostatic measures

##### Recommendation 22

We recommend the use of topical haemostatic agents in combination with other surgical measures or with packing for venous or moderate arterial bleeding associated with parenchymal injuries (Grade 1B).

#### Rationale

A wide range of local haemostatic agents is currently available for use as adjuncts to traditional surgical techniques to obtain haemorrhagic control. These topical agents can be particularly useful when access to the site of bleeding is challenging. The use of topical haemostatic agents should consider several factors, such as the type of bleeding, severity, coagulation status and each agent’s specific characteristics. Relatively extensive experience in humans is now available [231–240]. In a retrospective database review of the UK Joint Trauma Registry, it was concluded that the application of haemostatic dressings in severely war-injured patients increased survival [238].

The many different types of local haemostatic agents are based on collagen, sometimes combined with a procoagulant [233], gelatine alone or combined with a procoagulant [231, 234, 235, 237], absorbable cellulose [236] or oxidised cellulose impregnated with polyethylene glycol or salts to achieve more rapid haemostasis. Other products based on fibrin and synthetic glues or adhesives have sealant and haemostatic properties [232]. In addition, poly-N-acetyl-glucosamine derived from chitin, minerals such as kaolin and zeolite have demonstrated haemostatic effects [238–240].

### V. Initial management of bleeding and coagulopathy

#### Antifibrinolytic agents

##### Recommendation 23

We recommend that tranexamic acid (TXA) be administered to the trauma patient who is bleeding or at risk of significant bleeding as soon as possible, if feasible en route to the hospital, and within 3 h after injury at a loading dose of 1 g infused over 10 min, followed by an i.v. infusion of 1 g over 8 h (Grade 1A).

We recommend that the administration of TXA not await results from a viscoelastic assessment (Grade 1B).

#### Rationale

TXA has become one of the mainstays of therapy for the injured patient at risk of bleeding [241]. Since publication of the CRASH-2 trial, which showed a reduction in mortality of 1.5% and a reduction in bleeding deaths by one-third, there have been further trials evaluating TXA in TBI [242–245], different TXA dosing regimens [49, 243] and TXA in the pre-hospital setting [49, 243].

The largest TBI study, CRASH-3 (*n* = 12,737), compared 1 g bolus TXA followed by 1 g 8 h infusion i.v. TXA with matched placebo. In patients treated within 3 h of injury (*n* = 9202), the risk of head injury-related death was 18.5% (TXA) versus 19.8% [placebo; risk ratio (RR) 0.94, 95% CI 0.86–1.02]. Early treatment was shown to reduce death in mild and moderate head injury (RR 0.78, 95% CI 0.64–0.95) but not in severe head injury (RR 0.99, 95% CI 0.91–1.7) [246]. TXA was highly cost-effective for mild and moderate head injury and in patients in which both pupils reacted [246]. Participants with milder head injury may have benefited more from TXA because of a smaller baseline bleed volume [247]. Two RCTs published recently investigating patients with TBI were small (*n* = 100 [244]) and (*n* = 149 [245]), single-centre and found no difference between TXA and placebo for primary endpoints of intracranial haemorrhage [244] or intracerebral haematoma growth over 24 h [245], respectively.

Two RCTs examined pre-hospital TXA, using varying drug dosing [49, 243]. In a RCT investigating patients with TBI, 966 patients (GCS < 12) were treated with TXA or placebo using an out-of-hospital bolus and an in-hospital infusion regimen. Two different doses of TXA were used: 1 g bolus and 1 g infusion, or 2 g bolus and placebo infusion. No difference was reported in the primary endpoint, the Glasgow Outcome Scale-Extended score > 4 at 6 months, 65% combined TXA group vs. 62%, placebo [243]. In a general trauma RCT (*n* = 927), pre-hospital TXA (1 g over 10 min in 100 mL saline) was compared with matched placebo, given within 2 h of injury [49]. Mortality at 30 days was 9.9% with placebo versus 8.1% with TXA (no difference) [49]. Subsequent in-hospital dosing for the TXA arm followed three routes: no additional TXA, 1 g infusion, 1 g bolus followed by a 1 g infusion, with 30-day mortality rates of 9.3%, 7.8%, 7.3%, respectively (10% for placebo only) [49]. Participants administered TXA within 1 h of injury and a shock index < 0.9, had a 65% lower likelihood of 30-day mortality (HR 0.35, 95% CI 0.19–0.65), as well as a lower incidence of multiorgan failure and 24 h transfusion requirements compared with those with delayed (> 1 h from injury) TXA treatment [248].

There have been questions raised about whether TXA should be administered only to those patients with evidence of hyperfibrinolysis, following concerns that some patients are in a state of fibrinolytic shutdown (TEG LY30 < 0.9%). In the pre-hospital TBI RCT described above, 700 of the 966-strong cohort had TEG samples analysed at 0 and 6 h [249]. There was an equal spread of fibrinolytic TEG phenotypes, in particular shutdown across the 3 groups, with no increased incidence in those treated with TXA. VEM measures did not change over 6 h, despite changes to CCT measures of lysis. This finding led the authors to suggest that TEG may be poorly sensitive to fibrinolysis [249].

#### Coagulation support

##### Recommendation 24

We recommend that monitoring and measures to support coagulation be initiated immediately upon hospital admission (Grade 1B).

#### Rationale

While several general pathophysiological mechanisms have been described that result in traumatic coagulopathy, including low fibrinogen levels and hyperfibrinolysis [6, 124, 250], it is essential to quickly determine the type and degree of coagulopathy in the individual patient to identify the most prominent cause or causes, including the presence of anticoagulants, to be treated specifically and in a goal-directed manner [6]. Early and goal-directed therapeutic intervention improves coagulation [251, 252], which can reduce the need for transfusion of pRBC, FFP and platelets [5, 251–253], decrease post-traumatic multiorgan failure [251], length of hospital stay [252] and improve survival [4, 5, 253–255]. In contrast, no general survival benefit could be found in other studies [6, 125]. However, in most of the studies decisions on therapeutic interventions were primarily based on traditional laboratory values such as PT, APTT and platelet count, and treatment limited to FFP and platelet transfusions. In the study by Baksaas-Aasen et al., all patients received initial treatment according to empiric massive haemorrhage protocols (pRBC:plasma:platelets in a 1:1:1 ratio) and were then randomised into augmented viscoelastic or conventional coagulation testing-guided interventions. Despite a somewhat higher early fibrinogen administration in the viscoelastic testing group, no overall difference in outcome (24 h after injury alive or free of MT) was observed, with the exception of patients with TBI who showed a reduced 28-day mortality in the viscoelastic testing group, a predefined secondary outcome [125]. The overall outcome in this study is not surprising since the treatment algorithm defined very similar treatment in terms of blood products, cryoprecipitate and fibrinogen concentrate, irrespective of augmented viscoelastic or conventional coagulation testing [256].

#### Initial coagulation resuscitation

##### Recommendation 25

In the initial management of patients with expected massive haemorrhage, we recommend one of the two following strategies:

- Fibrinogen concentrate or cryoprecipitate and pRBC (Grade 1C)
- FFP or pathogen-inactivated FFP in a FFP/pRBC ratio of at least 1:2 as needed (Grade 1C)

In addition, we suggest a high platelet/pRBC ratio (Grade 2B).

#### Rationale

For initial resuscitation between hospital arrival and results from coagulation monitoring, early transfusion with FFP, platelets and pRBC in fixed ratios may improve survival and haemostasis, but data are equivocal. The PROPPR trial randomised 680 trauma patients to early FFP:platelets:pRBC administered 1:1:1 (platelets administered as part of first transfusion pack) or 1:1:2 (platelets with second pack). Mortality was comparable, but the 1:1:1 group showed improved haemostasis and reduced exsanguination deaths [257]. A recent literature review suggested that MT protocols in adult trauma patients should utilise ratios between 1:1:1 and 1:1:2 [258].

The pre-emptive administration of platelets as part of a fixed-ratio blood product strategy in massive bleeding is controversial. Further analysis of the PROPPR trial data suggests that transfusion of platelets in bleeding patients is associated with significantly decreased 24 h (5.8% vs. 16.9%; *p* < 0.5) and 30-day mortality (9.5% vs. 20.2%; *p* < 0.5), more patients achieving haemostasis (94.9% vs. 73.4%; *p* < 0.1) and fewer deaths as a result of exsanguination (1.5% vs. 12.9%; *p* < 0.1), without an increase in complications such as acute respiratory distress syndrome (ARDS), multiorgan failure and acute kidney injury [259]. Receiving higher ratios of platelets and plasma relative to pRBC hastens haemostasias in subjects who have yet to achieve haemostasis within 3 h after hospital admission [260]. In patients with MT, early platelet transfusion within 4 h was associated with lower rate of multiorgan failure and mortality within 30 days post-injury, although with a higher rate of ventilator-associated pneumonia and wound infections [261]. Similarly, in a large trauma database early platelet transfusion within 6 h was associated with decreased 24 h mortality both in patients with massive and non-massive transfusion [262].

Contrasting with empirical treatment using fixed FFP/platelet/pRBC ratios, and to avoid the adverse effects associated with FFP transfusion, several European centres strongly support the use of coagulation factor concentrates (CFCs) for first-line coagulation resuscitation in patients with significant bleeding and coagulopathy [263]. Critical fibrinogen levels (< 1.5 g/L) are reached in many massively injured patients at admission, and initial fibrinogen levels below normal predict in-hospital mortality in major trauma patients. FFP is impractical for increasing fibrinogen levels > 1.5 g/L; modelling shows that levels > 1.8 g/L are extremely difficult, if not impossible, to achieve, as the volume required increases exponentially as the targeted fibrinogen level approaches that in therapeutic plasma (≈2 g/L) [264].

Apart from dilutional effects and poor efficacy in increasing fibrinogen levels > 1.5–2.0 g/L in massively bleeding patients, transfusion of plasma cannot be initiated at the same time as pRBC transfusion and delays in achieving the targeted plasma/pRBC ratio may occur. For initial coagulation support, while awaiting viscoelastic or laboratory tests, the administration of 2 g fibrinogen based on clinical criteria at admission (systolic blood pressure < 100 mmHg, lactate ≥ 5 mmol/L, base excess ≤ − 6 or haemoglobin ≤ 9 g/dL) has been proposed, to mimic the 1:1 ratio corresponding to the first 4 units of pRBC and potentially correct hypofibrinogenemia [265].

A randomised comparison of fibrinogen concentrate and cryoprecipitate in hypofibrinogenaemic trauma patients (FEISTY) found that both treatments effectively increased plasma fibrinogen, with greater elevation in fibrin-based clot amplitude after the first administration with fibrinogen concentrate [FIBTEM A5 mean difference 2.6 mm (95% CI 1.1–4.1 mm), *p* = 0.001] [266]. Placebo-controlled trials have also demonstrated improved clot stability and fibrinogen levels in trauma patients receiving fibrinogen concentrate. In a prospective trauma registry, fibrinogen concentrate administration within the first 6 h of traumatic haemorrhagic shock did not significantly reduce 24 h all-cause mortality [267].

### VI. Further goal-directed coagulation management

#### Goal-directed therapy

##### Recommendation 26

We recommend that resuscitation measures be continued using a goal-directed strategy, guided by standard laboratory coagulation values and/or VEM (Grade 1B).

#### Rationale

Multiple sources of retrospective evidence have confirmed the benefits of goal-directed strategies guided by POC viscoelastic monitoring (VEM, e.g. TEG/ROTEM; [268, 269]) or conventional coagulation assays (CCAs; [270, 271]) to augment damage control resuscitation in bleeding trauma patients. VEM is highly specific for hyperfibrinolysis, the most lethal and resource-intensive phenotype of fibrinolysis in trauma, and is more sensitive in the detection of coagulopathy than CCAs [272]. POC VEM-based treatment algorithms, including thresholds to initiate goal-directed therapies with blood products, coagulation factors and haemostatic agents, have been introduced [273, 274] and their successful implementation demonstrated ([272]. The introduction of POC ROTEM has altered blood product transfusion practices for major trauma patients [275], with faster decision-making/initiation of therapies to correct coagulopathy [272], improvement in functional blood clotting parameters [276, 277] and safer transfusion strategies [269], including better survival [255] and cost savings [254, 269, 278].

Early goal-directed haemostatic resuscitation of traumatic coagulopathy guided by TEG^®^ was explored in a single-centre, pragmatic prospective RCT in 111 patients, and survival in the TEG^®^ group was significantly higher than in the CCA group with less use of plasma and platelets [253]. In the prospective RETIC study, an indirect benefit in favour of VEM was noted, as its use was precondition to demonstrate a survival benefit resulting from targeted coagulation factor supplementation [251]. The iTACTIC trial was a multi‐centre RCT that compared outcomes defined as alive and free of MT (≥ 10 pRBC) at 24 h after injury among 396 trauma patients treated according to empiric massive haemorrhage protocols, augmented by optimised VEM or CCA‐guided interventions [125]. While there was no difference between groups in the intention-to-treat analysis, there was a trend towards improved survival in the pre-specified subgroup that was coagulopathic (INR > 1.2), which became significant in the subgroup with TBI (OR 2.12, 95% CI 0.84–5.34). In a single-centre pre- and post-implementation study that included 201 patients with major haemorrhage mortality was significantly lower in the post-TEG group at 24 h (13% vs. 5%; *p* = 0.006) and 30 days (25% vs. 11%; *p* = 0.002), with significantly less blood product wastage [254]. In isolated TBI with VEM-identified coagulopathy and treatment requiring craniotomy, the rate of progressive haemorrhagic injury and for neurosurgical re-intervention was significantly lower [277]. Blood transfusion due to bleeding [279] or acutely bleeding trauma [280], TBI [127] or in patients undergoing surgical procedures [281] consistently demonstrated a survival benefit with VEM. The known transfusion-limiting effect with VEM was confirmed across four of these five studies [279–282]. TEG/ROTEM‐guided transfusions were also associated with fewer additional invasive haemostatic interventions (angioembolic, endoscopic or surgical) in surgical patients [280] and reduced the risk of acute kidney injury in mixed patient groups [280–282]. However, the Cochrane review [282], the three meta-analyses [279–281] and the one subgroup meta-analysis of two RCTs [127] were of overall low to moderate quality, including risk of bias on the use of TEG/ROTEM to monitor and guide haemostatic treatment/transfusion versus non-TEG/ROTEM- or standard-of-care with/without CCA-guided blood transfusion in patients with bleeding [282].

#### Fresh frozen plasma-based management

##### Recommendation 27

If a FFP-based coagulation resuscitation strategy is used, we recommend that further use of FFP be guided by standard laboratory coagulation screening parameters (PT and/or APTT > 1.5 times normal and/or viscoelastic evidence of a coagulation factor deficiency) (Grade 1C).

We recommend that the use of FFP be avoided for the correction of hypofibrinogenemia if fibrinogen concentrate and/or cryoprecipitate are available (Grade 1C).

#### Rationale

Plasma (thawed FFP or pathogen-inactivated plasma) is used in many countries to treat traumatic coagulopathy. However, although plasma contains all pro- and anticoagulant factors, FFP contains only ~ 70% the normal level of all clotting factors. The transfusion of plasma might have protective effects on haemorrhage-induced glycocalyx disruption [283], but is also associated with increased risk of several adverse events [284]. A retrospective study identified FFP transfusion as an independent risk factor for mortality after severe TBI [285].

When a FFP-based coagulation resuscitation strategy is used, retrospective analysis [286] and the randomised PROPPR study [257] have suggested that early transfusion of plasma in a balanced ratio of 1:1 with pRBC is associated with higher rates of haemostasis and lower rates of mortality and exsanguination in patients with critical haemorrhage when compared with a ratio of 1:2, although the optimal ratio has not yet been established. A subsequent analysis of the PROPPR study showed that earlier time to haemostasis was independently associated with decreases in 30-day mortality, acute kidney injury, ARDS, multiorgan failure and sepsis in bleeding trauma patients [287]. Despite limited scientific evidence, FFP administration should be guided by evidence of coagulation factor deficiency, as indicated by PT or APTT > 1.5 times the normal control or by prolongation of viscoelastic parameters such as clotting time or reaction time.

Different plasma preparations show wide variability; FFP contains a variable amount of fibrinogen and other coagulation factors [284]. A prospective cohort study found no consistent correction of clot function or increases in procoagulant factor concentrations following FFP transfusion during the acute phase of ongoing bleeding [288]. Ex vivo the use of CFCs for the reconstitution of blood achieves higher haematocrit and fibrinogen content compared with FFP [289]. The RETIC randomised trial showed that FFP was insufficient to correct hypofibrinogenemia or significantly improve clot strength vs. fibrinogen concentrate in adult trauma patients [251]. A high proportion of patients in the FFP group required crossover rescue therapy using CFCs, whereas rescue therapy was much less frequent in the CFC group [23 patients (52%) vs. 2 patients (4%), respectively; OR 25.34 (95% CI 5.47–240.03), *p* < 0.0001] [251]. In another randomised comparison, patients with traumatic coagulopathy received fibrinogen concentrate, FFP or no product; the need for pRBC, intravenous fluid in the first 24 h of hospitalisation and ICU admission, as well as the rates of sepsis and mortality, were all significantly lower in the fibrinogen concentrate group [290].

Pathogen-inactivated plasma has a more standardised fibrinogen content and minimises the risk of transfusion-related acute lung injury (TRALI) and infection compared with FFP. The use of readily transfusable liquid plasma has been shown to enable a higher plasma/pRBC ratio within the first hour of transfusion [291], thus potentially increasing the efficacy to prevent coagulopathy. A recent metanalysis and retrospective data found no difference in mortality when using liquid or thawed plasma in trauma patients [292, 293].

With a relative shortage of type AB plasma, to allow transfusion of plasma for resuscitation of patients whose blood type is unknown, the use of ABO-incompatible plasma in the form of group A plasma for trauma patients of unknown ABO group is increasingly being investigated. The majority of available studies were retrospective and showed no significant increases in morbidity or mortality. A secondary analysis from the PROPPR trial showed that MT of incompatible type A plasma to patients with blood group B or AB was not associated with significantly increased morbidity [294].

#### Coagulation factor concentrate-based management

##### Recommendation 28

If a CFC-based strategy is used, we recommend treatment with factor concentrates based on standard laboratory coagulation parameters and/or viscoelastic evidence of a functional coagulation factor deficiency (Grade 1C).

Provided that fibrinogen levels are normal, we suggest that prothrombin complex concentrate (PCC) is administered to the bleeding patient based on evidence of delayed coagulation initiation using VEM (Grade 2C).

We suggest that monitoring of FXIII be included in coagulation support algorithms and that FXIII be supplemented in bleeding patients with a functional FXIII deficiency (Grade 2C).

#### Rationale

Traumatic coagulopathy is characterised by a low fibrinogen concentration and often an increased fibrinolytic activity [6, 124, 250]. Besides early administration of TXA (see recommendation R23) early fibrinogen administration (see recommendation R29) is also of key importance, ideally guided by a fibrinogen concentration < 1.5 g/L or viscoelastic evidence of a functional fibrinogen deficiency [6]. Since the specific coagulation situation varies between patients and over time, the exact needs of each individual patient must be determined based on standard laboratory coagulation parameters and/or viscoelastic evidence of a functional coagulation factor deficiency [6].

The usefulness of PCC has been demonstrated, with evidence of reduced haematoma formation in patients with head injury [295], and is preferable to FFP for the rapid reversal of the effects of vitamin K antagonists (VKAs) [296] (see recommendation R33). In a recent meta-analysis comparing the treatment of patients with trauma-induced coagulopathy with FFP alone vs. FFP plus PCC, the addition of PCC was found to decrease the transfusion of pRBC and FFP and to decrease mortality significantly without increasing thromboembolic adverse events [297]. This meta-analysis, however, was limited to 3 retrospective studies in 840 patients. PCC may also be used in the treatment of trauma patients anticoagulated with Xa inhibitors (see recommendation R34).

VEM is useful to guide individualised goal-directed coagulation therapy in patients with traumatic coagulopathy [6]. In the initial phase, a low fibrinogen concentration is expected. However, thrombin generation is preserved or even increased [298]. Initial treatment should therefore comprise fibrinogen administration, which not only increases the maximum clot firmness in FIBTEM, but also shortens the clotting time in EXTEM [276]. Only if the EXTEM clotting time remains prolonged, despite a fibrinogen level > 1.5 g/L should PCC be administered to normalise the EXTEM clotting time [299].

It is important to avoid the overly liberal use of PCC in trauma patients, because PCC administration results in increased thrombin potential over days that is not reflected by standard laboratory tests and might expose the trauma patient to an increased risk of delayed thrombotic complications [300]. Therefore, the risk of thrombotic complications resulting from PCC treatment should be weighed against the need for rapid and effective correction of coagulopathy [296].

Coagulation factor XIII (FXIII), formerly known as a “fibrin stabilising factor” is circulating in tetrameric form consisting of two A and two B subunits. The A subunit of FXIII is activated to FXIIIa by thrombin and FXIIIa catalyses the cross-linking of fibrin. Strong cross-linking of fibrin prevents fibrinolysis and FXIII activity seems to be an important independent modulator of clot firmness [301].

Low levels of FXIII have been found in patients with major trauma and coagulopathy. If cryoprecipitate is not available, as in most European countries, and a CFC-based strategy is used, very little if any factor XIII is administered. Monitoring factor XIII levels and replacement below a certain threshold therefore is suggested as part of coagulation support algorithms. At present, however, the need for and a defined optimal level of FXIII replacement in major trauma patients has not been determined. The updated ESA guidelines for the management of severe perioperative bleeding suggest the administration of FXIII concentrate in the presence of bleeding and a FXIII level < 30% [302]. The use of FXIII concentrate at a FXIII level < 60% was part of multimodal algorithms in two recent studies in major trauma patients, resulting in major reductions in transfusion requirements and improvements in clinical outcomes, including a reduction in the duration of stay in the ICU, organ dysfunction and hospital mortality in one study [5, 251].

#### Fibrinogen supplementation

##### Recommendation 29

We recommend treatment with fibrinogen concentrate or cryoprecipitate if major bleeding is accompanied by hypofibrinogenemia (viscoelastic signs of a functional fibrinogen deficit or a plasma Clauss fibrinogen level ≤ 1.5 g/L) (Grade 1C)*

We suggest an initial fibrinogen supplementation of 3–4 g. This is equivalent to 15–20 single donor units of cryoprecipitate or 3–4 g fibrinogen concentrate. Repeat doses should be guided by VEM and laboratory assessment of fibrinogen levels (Grade 2C).

#### Rationale

Cryoprecipitate and fibrinogen concentrate (FC) have now been prescribed to trauma patients for more than 10 years without any evidence-based support. Up to now, no large double-blind RCT has confirmed the validity of this strategy. A randomised controlled feasibility trial showed that early fibrinogen supplementation with cryoprecipitate was feasible in trauma patients [303]. No difference in transfusion was observed; however, the study was not adequately powered. Only five very small recent RCTs are available [290, 304–307] for FC. For three studies, the primary outcome was feasibility in a restricted time frame, which was only reached for two of them [305, 306]. One study selected clot stability with FIBTEM as a primary outcome and confirmed the feasibility of early pre-hospital administration [307]. Of these four studies, none was powered to assess any difference in transfusion requirements, even if no difference was observed between the control groups and the FC groups. One study compared the effect of FC, FFP and no plasma-no FC (control) on the mortality of trauma patients [290]. The difference was highly in favour of FC and there was also a significant difference in transfusion amounts and other major outcomes, but this study was not blinded and had major biases, imprecision and inconsistency. Two retrospective registry studies with injury severity score-matched control groups and a propensity analysis showed no difference between FC-treated patients and control patients for all-cause mortality or transfusion [267, 308]. Finally, a systematic review and meta-analysis showed that there was no difference between FC and control for mortality, pRBC, FFP or platelet transfusion requirements or thromboembolic events, with a low quality of evidence [309].

*Based on the weak level of the evidence, the proposed grading of this recommendation was 2B. However, following in-depth discussion, some authors suggested that either 2B or 1C might be appropriate. The voting results were split: 50% objected to and 39% supported a grading of 2B, while 11%, representing the non-voting authors, abstained. The group therefore decided to revert the grading to 1C, as in the previous edition of the guideline, because several authors felt that downgrading to a suggestion might risk misinterpretation to the detriment of fibrinogen concentrate use as part of daily clinical practice.

#### Platelets

##### Recommendation 30

We suggest that platelets be administered to maintain a platelet count above 50 × 10^9^/L in trauma patients with ongoing bleeding and above 100 × 10^9^/L in patients with TBI (Grade 2C).

If administered, we suggest an initial dose of four to eight single platelet units or one aphaeresis pack (Grade 2B).

#### Rationale

While a low platelet count has been consistently associated with both morbidity and mortality in trauma patients, the threshold and timing of platelet transfusion remains controversial [6]. Although the platelet count at admission was found to be a biomarker for trauma severity and predictive of outcome, including bleeding intensity and transfusion requirements [262], platelet counts are frequently within the normal range at hospital admission, but may decrease sharply in the following 1–2 h of haemostatic resuscitation and decline continuously thereafter, suggesting an important role for the treatment administered [310].

No randomised trials have investigated specific platelet transfusion thresholds in trauma patients. Several observational studies have investigated associations between platelet transfusion and outcome; however, these are subject to an inherent risk of bias such as immortal time bias as well as bias from residual confounding. Accordingly, there is currently weak scientific evidence to support a specific platelet transfusion threshold in the bleeding trauma patient. In a sub-study of a RCT, patients randomised to receive first-line coagulation factors or FFP were also transfused with platelets to maintain platelet counts between 50 and 100 × 10^9^/L [310]. Platelet transfusion did not substantially improve platelet count and contributed to poor clinical outcome.

In patients with TBI, the benefit of platelet transfusion is also controversial [133, 136]. However, in patients with severe TBI, if ADP response improved after platelet administration, the need for neurosurgical intervention decreased, as well as mortality [138].

The therapeutic dose of platelets is four to six units of pooled platelets, equivalent to one aphaeresis platelet product, which contains approximately 3–4 × 10^11^ platelets [311]. This dose is usually sufficient to provide haemostasis in a thrombocytopenic bleeding patient and should increase the platelet count by > 30 × 10^9^/L. However, the recovery rate in peripheral blood may be lower under conditions associated with increased consumption and transfusion of one unit of platelets may be insufficient to improve haemostasis in trauma patients.

The effect of higher platelet doses as well as empiric platelet transfusion in trauma patients without thrombocytopenia as part of a balanced transfusion strategy with other blood products is controversial. Data from French [262] and US [312] trauma registries support platelet transfusion despite a normal platelet count [262] and maintenance of a platelet/pRBC ratio closer to1:1, respectively. Recent systematic reviews also found that higher platelet/pRBC ratios result in a significant decrease in short-term (24 h) and long-term (28–30 day) mortalities [313, 314], lower ICU length of stay (LOS) and higher ICU-free days [314], without influencing the occurrence of thromboembolic events or organ failure [313] when compared with lower platelet/pRBC ratios. These results should be interpreted with extreme caution, as many source studies are prone to various types of bias, including various definitions of high and low platelet/pRBC ratios, severity of bleeding and MT, as well as different platelet products with different storage times, administered at different time intervals. As such, a specific platelet/pRBC ratio for empiric transfusion cannot be recommended at present.

The optimal timing of platelet transfusion in association with traumatic bleeding also requires clarification. Further analysis of the PROPPR study showed that the impact of transfusion ratios on haemostasis is dynamic and the longer it takes to achieve haemostasis, the more likely high blood product transfusion ratios, including platelets, may be beneficial in terms of both haemostasis and survival [260]. Others observed increasing effects of platelet transfusion on platelet aggregation over time, identifying a potential early period of resistance to platelet transfusion that resolves by 72–96 h [315]. Late transfusions (after 48 h) resulted in a larger increase in platelet numbers.

#### Calcium

##### Recommendation 31

We recommend that ionised calcium levels be monitored and maintained within the normal range following major trauma and especially during massive transfusion (Grade 1C).

We recommend the administration of calcium chloride to correct hypocalcaemia (Grade 1C).

#### Rationale

The normal range of ionised calcium (Ca^2+^) is 1.1–1.3 mmol/L and is pH-dependent, with a 0.1 unit increase in pH decreasing ionised calcium concentration by approximately 0.05 mmol/L [316]. Ionised calcium is essential not only for the formation and stabilisation of fibrin polymerisation sites but also for many platelet-related functions, with a reduction in the concentration of calcium negatively impacting both processes [316]. In addition, cardiac contractility and systemic vascular resistance are impaired in the presence of reduced ionised calcium levels. Importantly, laboratory tests do not accurately reflect the detrimental effect of hypocalcaemia on the coagulation cascade, as blood samples are citrated and then subsequently recalcified prior to being analysed.

Acute hypocalcaemia is both a common finding in trauma patients and frequently complicates MT [317, 318]. Low calcium concentrations at admission are associated with platelet activation, aggregation, decreased clot strength, blood transfusions and increased mortality [319–321]. In patients receiving blood transfusions, hypocalcaemia results from the citrate-mediated chelation of serum Ca^2+^. Each unit of pRBC or FFP contains approximately 3 g of citrate used as a preservative and anticoagulant. This citrate is normally metabolised by mitochondria in the liver to bicarbonate in a matter of minutes. However, in the context of haemorrhagic shock requiring massive transfusion, liver function is often impaired due to hypoperfusion. The resulting hypocalcaemia is detrimental chiefly because of the pivotal role Ca^2+^ plays in the coagulation cascade. Ca^2+^ acts as a cofactor in the activation of factors II, VII, IX and X, as well as proteins C and S. It is also necessary for platelet adhesion at the site of vessel injury. Remarkably, hypocalcaemia within the first 24 h of critical bleeding can predict mortality and the need for multiple transfusions with greater accuracy than the lowest fibrinogen concentration, acidosis and the lowest platelet count [322]. Ionised calcium levels are also easily monitored, as they are included as a standard part of a blood gas analysis by the majority of blood gas analysers available on the market.

Transfusion-induced hypocalcaemia, with ionised Ca^2+^ levels below 0.9 mmol/L or serum total corrected calcium levels of 7.5 mg/dL or lower, should be corrected promptly, as ionised Ca^2+^ levels below 0.8 mmol/L are associated with cardiac dysrhythmias. Importantly, however, it should be noted that while an association between admission ionised hypocalcaemia and mortality, increased blood transfusion and coagulopathy has been identified, no data demonstrate that the prevention or treatment of ionised hypocalcaemia reduces mortality in patients with critical bleeding requiring MT.

The preferred agent to correct hypocalcaemia is calcium chloride, 10 mL as a 10% solution contains 270 mg of elemental calcium. In comparison, 10 mL of 10% calcium gluconate contains only 90 mg of elemental calcium [323]. Calcium chloride may also be preferable to calcium gluconate in the setting of abnormal liver function, where decreased citrate metabolism results in the slower release of ionised calcium.

#### Recombinant activated coagulation factor VII

##### Recommendation 32

We do not recommend the use of recombinant activated coagulation factor VII (rFVIIa) as first-line treatment (Grade 1B).

We suggest that the off-label use of rFVIIa be considered only if major bleeding and traumatic coagulopathy persist despite all other attempts to control bleeding, systemic homeostasis and best-practice use of conventional haemostatic measures (Grade 2C).

#### Rationale

rFVIIa acts on the endogenous coagulation system, but its effect depends on adequate numbers of platelets and fibrinogen, pH and body temperature near normal levels. Primary predictors of a poor response to rFVIIa treatment are pH < 7.2 (*p* < 0.0001) and platelet count < 100 × 10^9^/L (*p* = 0.046) [324].

Administration of rFVIIa as an adjunct to standard care in severely bleeding trauma patients did not affect mortality [325]. A Cochrane systematic review concluded that the efficacy of rFVIIa outside its current licensed indications is unproven and even associated with an increased incidence of arterial thromboses, therefore rFVIIa should only be used for licensed indications or in the context of a study [326]. In the context of bleeding major trauma patients, rFVIIa should be considered only if treatment with a combination of surgical approaches, best-practice use of blood products, antifibrinolytics and correction of severe metabolic acidosis, hypothermia and hypocalcaemia fail to control bleeding. Best-practice use of blood products includes pRBC, platelets, FFP and cryoprecipitate/fibrinogen targeting to Hct above 24%, platelets above 50 × 10^9^/L and fibrinogen above 1.5–2.0 g/L.

In patients with isolated head injury and TICH, the use of rFVIIa was shown to have no positive effect on patient outcomes, and even found to be harmful [327, 328]. Accordingly, a Cochrane systematic review found no support in favour of rFVIIa treatment for reducing mortality or disability in patients with TBI and related ICH [329].

The use of rFVIIa to treat traumatic coagulopathy represents an "off-label" indication and its administration was associated to increased risk of thromboembolic complications [330, 331]. However, the recent evidence did not find elevated thromboembolic complications in severe trauma patients receiving rFVIIa [325, 332].

### VII. Management of antithrombotic agents

#### Reversal of vitamin K-dependent oral anticoagulants

##### Recommendation 33

In the bleeding trauma patient, we recommend the emergency reversal of vitamin K-dependent oral anticoagulants with the early use of both PCC and 5–10 mg i.v. phytomenadione (vitamin K_1_) (Grade 1A).

#### Rationale

VKAs such as warfarin, are still prescribed, despite the increasing use of direct oral anticoagulants (DOACs) [333], for the prevention of thromboembolism in atrial fibrillation, previous venous or arterial thromboembolism and/or mechanical heart valves. There are three therapeutic options for the reversal of VKAs: vitamin K, PCC and FFP. Modern guidelines advise the rapid restoration of a normal INR, although evidence that this improves clinical outcome is limited to case series [295, 334–338], one suggesting more improvement if PCC was administered rapidly [337].

For the immediate reversal of VKAs, the missing coagulation factors, FII, FIX and FX, are replaced with PCC [339]. However, correction of INR is particularly dependent on FVII and there are low levels of FVII in three-factor PCC. Unfortunately, some countries only have access to three-factor PCC [340]; however, the use of three-factor PCC is not recommended if four-factor PCC is available. Because the half-life of administered FVII is only about six hours, it is important that vitamin K1 (phytomenadione) is co-administered with PCC to stimulate production of the vitamin K-dependent coagulation factors after the initial effect of PCC.

FFP contains the missing coagulation factors diluted among all the other constituents of plasma. However, large volumes of FFP are required to replace the missing factors, thus reversal is often not achieved and there are risks of transfusion-associated circulatory overload and TRALI [339]. Indeed a review of 19 studies that included 2878 patients showed that PCC provides more rapid and complete factor replacement for PCC versus FFP [341], and thromboembolic complications were less frequent in PCC recipients (2.5%) than FFP recipients (6.4%). However, similar poor clinical outcomes were seen in both groups [341].

Four-factor PCC is administered intravenously in a dose of 25–50 U/kg simultaneously with vitamin K and there are algorithms to calculate the most appropriate dose based on body weight and INR level [339]. A stepwise dosage is recommended, e.g. 25 U/kg if INR is 2–4.0, 35 U/kg if INR is 4–6.0 and 50 U/kg if INR is > 6.0 [342]. With difficult i.v. access, intraosseous infusion of PCC has been used with no apparent detrimental effects [343]. After reversal, INR should be monitored regularly over the next week, as a minority of patients take over a week to clear warfarin from their blood and require additional vitamin K [344]. A rare and unpredictable, but important, side effect of i.v. vitamin K is an anaphylactic reaction, which can result in cardiac arrest, with an incidence of 3 per 100,000 doses via a non-immunoglobulin E (IgE) mechanism, possibly due to the solubiliser in the vitamin K solution [345].

We recommend a 5–10 mg dose of vitamin K because less may not fully correct the INR and conversely more than 10 mg vitamin K_1_ can prevent re-warfarinisation for days and may create a prothrombotic state, which could lead to further thromboembolism [339].

The use of PCC is associated with an increased risk of venous and arterial thrombosis during the recovery period, due to pre-existing risk and possibly the use of PCC itself [339]. In addition, higher incidences of thromboembolic events have been reported in trauma patients with the use of three-factor PCC compared with four-factor PCC [346]. Therefore, in patients who have received PCC, thromboprophylaxis must be considered as early as possible after bleeding has been controlled.

#### Management of direct oral anticoagulants—factor Xa inhibitors

##### Recommendation 34

We suggest the measurement of plasma levels of oral direct antifactor Xa agents such as apixaban, edoxaban or rivaroxaban in patients treated or suspected of being treated with one of these agents (Grade 2C).

We suggest that measurement of anti-Xa activity be calibrated for the specific agent. If not possible or available, we suggest low molecular weight heparin (LMWH)-calibrated anti-Xa assays as a reliable alternative (Grade 2C).

If bleeding is life-threatening in the presence of an apixaban or rivaroxaban effect, especially in patients with TBI, we suggest reversal with andexanet alfa (Grade 2C).

If andexanet alfa is not available, or in patients receiving edoxaban, we suggest the administration of PCC (25–50 U/kg) (Grade 2C).

#### Management of direct oral anticoagulants—direct thrombin inhibitors

##### Recommendation 35

We suggest the measurement of dabigatran plasma levels using diluted thrombin time in patients treated or suspected of being treated with dabigatran (Grade 2C).

If measurement is not possible or available, we suggest measurement of the standard thrombin time to allow a qualitative estimation of the presence of dabigatran (Grade 2C).

If bleeding is life-threatening in those receiving dabigatran, we recommend treatment with idarucizumab (i.v. 5 g) (Grade 1C).

#### Rationale

The DOAC plasma concentration is the most important factor that determines whether an active reversal of medication is necessary. Increasing DOAC plasma levels progressively affect laboratory and viscoelastic coagulation tests. Early assessment of both laboratory coagulation tests and direct measurements of DOAC levels, therefore, is crucial in trauma patients receiving or suspected of having received a DOAC [347].

Measurement using three commonly available laboratory tests, PT, antifactor Xa and thrombin time, allows for the assessment of whether a patient is anticoagulated, and if so, by which agent, VKA, a FXa inhibitor or a thrombin inhibitor, respectively. If it is unknown with which DOAC the patient has been treated or the anti-Xa assay calibrated for the specific agent is not available, a universal, LMWH-calibrated, anti-Xa assay is a reliable alternative. This assay accurately determines rivaroxaban, apixaban and edoxaban concentrations and correctly predicts relevant drug concentrations [348]. An anti-Xa activity of 0.35 U/mL thereby corresponds to a DOAC cut-off value of 30 µg/L, 0.58 U/mL corresponds to 50 µg/L and 1.14 U/mL corresponds to 100 µg/L [348]. Viscoelastic coagulation tests may also be helpful, since most DOACs prolong the clotting time (ROTEM or ClotPro) progressively [349]. Apixaban has only a low impact on the clotting time even at high plasma concentrations; however, if the patient has a traumatic coagulopathy with a prolonged clotting time, it will not be possible to discriminate between this effect and the presence of a DOAC prior to treatment [350].

Andexanet alfa acts as a decoy target for the urgent reversal of rivaroxaban and apixaban [351]. The US Food and Drug Administration (FDA) approved andexanet alpha in May 2018, followed by the European Medicines Agency (EMA) in 2019. Andexanet alfa is administered as an intravenous bolus of 400 mg over 15 min followed by a continuous infusion of 480 mg over 2 h (low dose) or 800 mg over 30 min followed by a continuous infusion of 960 mg over 2 h (high dose) [351]. The dose and time since the last DOAC intake determine whether the low- or high-dosage protocol is applied. A propensity score-matched analysis showed that the adjusted 30-day mortality rates were lower for patients treated with andexanet alfa than in matched patients receiving PCC [352]. In patients suffering from an intracerebral haemorrhage andexanet alfa reduced anti-FXa activity with a high rate of haemostatic efficacy and a beneficial outcome [353]. Another comparison of andexanet alfa versus four-factor PCC reversal showed no significant difference regarding thrombotic events, but larger studies are required to confirm this finding [354]. In patients with acute major bleeding treated with edoxaban, initial evidence shows, that andexanet alfa significantly decreases anti-Xa activity with good haemostatic efficacy and may be considered as a reversal strategy [355]. Use in this context is, however, currently still off-label and further data are necessary to confirm this finding.

Plasma levels of antifactor Xa agents following the administration of andexanet alfa cannot be reliably measured with standard anti-Xa assays because the level of dilution causes dissociation of andexanet alfa from the anticoagulant, resulting in an overestimation of the anticoagulant concentration. Thus, modified anti-Xa assays with reduced dilution are available in some coagulation laboratories [356]. VEM tests such as ROTEM or ClotPro still can provide additional information about residual antifactor Xa activity [349, 350]; however, ROTEM tests are only minimally impacted by low DOAC levels [350].

Four-factor PCC antagonises the anticoagulant effect of factor X inhibitors. PCC increases prothrombin and factor X levels inducing a compensatory prohaemostatic effect with increased thrombin generation potential. Therefore, if antifactor Xa activity has been detected and andexanet alfa is not available or patients are on edoxaban, PCC (25–50 U/kg) treatment may be initiated. We suggest an initial dose of 25 U/kg, as this dose has been shown to provide effective haemostasis without increased rates of thromboembolic events [357]. Repeated PCC doses may be necessary in special cases, but caution should be used due to the possible thrombotic potential of PCC products.

In the presence of life-threatening bleeding and anti-FIIa activity due to dabigatran, treatment with idarucizumab (5 g i.v.) should be initiated [358, 359]. Repeated doses of idarucizumab may be necessary in patients with high dabigatran plasma levels [360]. Once idarucizumab has been administered coagulation tests should be repeated within 5–10 min (laboratory and viscoelastic tests). Only after dabigatran neutralisation, they are able to show the underlying coagulopathy usually present in patients following major trauma [361].

The co-administration of tranexamic acid (15 mg/kg or 1 g) is indicated in trauma patients independent of the present DOAC and reversal strategy (see recommendation R23).

#### Antiplatelet agents

##### Recommendation 36

We recommend that routine platelet transfusion be avoided in patients with ongoing bleeding who have been treated with APAs (Grade 1C).

#### Rationale

Conflicting data exist with respect to the effects of APAs on bleeding and outcome in trauma patients, with or without TBI [6]. One meta-analysis that included 24 observational studies and 5423 participants with early surgery for hip fracture found a higher risk of bleeding and higher blood transfusion requirements, but similar outcomes in patients taking APAs compared to those without antiplatelet therapy [362].

In mild TBI patients treated with APAs, two meta-analyses have demonstrated a small increased risk of immediate ICH, especially if concomitant with another risk factor for ICH such as GCS < 15 or age > 65 years [363] and a very low risk of delayed ICH [364], respectively. However, the subgroup of patients on dual antiplatelet therapy had an increased delayed bleeding risk, compared with single APA patients [364]. Another meta-analysis that included 20 observational studies comparing 2447 TBI patients on pre-injury APAs with 4814 controls revealed no statistically significant difference in early mortality, need for neurosurgery or LOS between the two groups, with similar results for subgroup analyses of aspirin and clopidogrel users [365].

The discrepant results on the risk of bleeding in patients taking APAs may be explained by the insufficient analysis of confounding factors that may influence the effects of different APAs in specific groups of patients. Evaluation of platelet function in bleeding trauma patients treated with suspected or confirmed pre-injury APAs may be useful to guide reversal therapies [139], but the role of current POC-PFTs is not yet established (see recommendation R12). Whether bleeding in the setting of APA use warrants platelet transfusion is also controversial.

Two meta-analyses of ten [366] and twelve [367] studies, respectively, mainly retrospective and including mostly aspirin-treated patients, showed a lack of evidence of survival benefit following platelet transfusion in TICH while on APAs. While there was no significant overall reduction in haemorrhage progression or need for neurosurgical intervention, sensitivity analysis demonstrated that among studies with larger sample sizes, platelet transfusion was associated with a reduced risk of haemorrhage progression but increased mortality [367]. The lack of survival benefit associated with platelet transfusion in patients with severe TBI on APAs was confirmed in a further prospective multicentric study [368]. However, in a single-institution study administration of two units of pooled platelets improved outcomes compared with one unit [369].

Another meta-analysis that included 16 clinical trials in both spontaneous and TICH patients on APAs found a significant difference for haematoma expansion in favour of platelet transfusion compared with standard of care, but no difference in mortality and severe disability, and a slight increase in the odds for adverse thromboembolic events following platelet transfusion [370].

Importantly, potential confounding factors in the studies on platelet transfusion in patients on APAs are the dose, the timing and the type of platelet product administered, as well as the type of APAs administered. However, few studies have specifically investigated the reversal of P2Y12 inhibitors after TICH. In a cohort of 243 patients with isolated TBI and ICH on pre-injury P2Y12 inhibitor, platelet transfusion was associated with a 32% decrease in the rate of progression of ICH, a 20% decrease in neurosurgical intervention, and a survival benefit on multivariate regression analysis [371].

Data on desmopressin as a potential alternative to platelet transfusion for reversal of APA effects in trauma patients are scarce, therefore, we have not made a recommendation on the use of desmopressin in this setting.

### VIII. Thromboprophylaxis

#### Thromboprophylaxis

##### Recommendation 37

We recommend early initiation of mechanical thromboprophylaxis with intermittent pneumatic compression (IPC) while the patient is immobile and has a bleeding risk (Grade 1C).

We recommend combined pharmacological and IPC thromboprophylaxis within 24 h after bleeding has been controlled and until the patient is mobile (Grade 1B).

We do not recommend the use of graduated compression stockings for thromboprophylaxis (Grade 1C).

We do not recommend the routine use of inferior vena cava filters as thromboprophylaxis (Grade 1C).

#### Rationale

The risk of hospital-acquired venous thromboembolism (VTE) after multiple trauma is high; a prospective study showed that without thromboprophylaxis 18% had proximal deep vein thrombosis (DVT) and 11% pulmonary embolism (PE) while PE is the third leading cause of death in those who survive beyond the third day [372].

There are few RCTs assessing thromboprophylaxis in trauma patients alone. Of particular note, there are none assessing the use of graduated compression stockings (GrCS) in trauma patients; indeed, there is no evidence to show that GrCS reduce the risk of death due to a PE in any hospitalised patients. Recently the GAPS study [373] failed to show any evidence of benefit with the use of GrCS in an RCT of over 2000 surgical patients with moderate VTE risk; while the CLOTS (Clots in Legs Or Stockings) studies in stroke patients showed that GrCS could cause harm [374].

In contrast to GrCS, there is good evidence to suggest that IPC is associated with a benefit in reducing hospital-associated VTE. A recent Cochrane review [375] on the use of combined IPC and pharmacological thromboprophylaxis compared with either alone, based mainly on surgical and trauma patients, concluded that combining IPC with pharmacological prophylaxis, compared with pharmacological prophylaxis alone, reduces the incidence of both PE (low-certainty evidence) and DVT (high-certainty evidence). In those with a bleeding risk, IPC alone is preferable until the bleeding risk recedes.

A systematic review and meta-analysis [376] showed that thromboprophylaxis with heparins decreases DVT and PE in critically ill medical and surgical patients, and LMWH compared with twice daily unfractionated heparin (UFH) decreases both the overall rate and symptomatic rate of PE. Furthermore, a large study in the elderly [377] showed LMWH was more efficacious than UFH and had a lower bleeding risk in a geriatric population of over 93,000. Weight-adjusted LMWH is widely used although good RCTs comparing standard versus weight adjusted are awaited. A 289-patient study of those who developed VTE during or after a critical care stay showed that thromboprophylaxis failure was more frequent with elevated body mass index, a personal or family history of VTE and those administered vasopressors [378]. However, there are inadequate data to suggest routine monitoring of LMWH with anti-Xa levels improves clinical outcome [378, 379].

Contraindications to pharmacological thromboprophylaxis include patients who are already receiving full-dose anticoagulation, those with significant thrombocytopenia (platelet count < 50 × 10^9^/L), an untreated inherited or acquired bleeding disorder, evidence of active bleeding, uncontrolled hypertension (blood pressure > 230/120), a lumbar puncture/spinal analgesia expected within the next 12 h or performed within the last 4 h (24 h if traumatic), procedures with a high bleeding risk or a new haemorrhagic stroke.

The optimal timing for the initiation of pharmacological thromboprophylaxis remains inadequately investigated, especially after TBI. Retrospective studies looking at TBI show that there are fewer VTE if thromboprophylaxis is started sooner (within 24–72 h of injury) rather than later, without increased bleeding risk [380], but how much earlier thromboprophylaxis can be used with efficacy and safety is the subject of future clinical trials. We suggest that pharmacological VTE prophylaxis be initiated with either LMWH, or low-dose UFH in patients with renal failure, as early as possible, only after a head CT confirms that ICH is stable and in the absence of persistent bleeding.

The use of prophylactic inferior vena cava (IVC) filters has been shown to be of no benefit in reducing a composite end point of symptomatic PE and death compared with no filter [381]. Moreover, a meta-analysis concluded that while IVC filters in this setting may reduce non-fatal PE they do not affect overall mortality [382]. Furthermore, there is also no evidence of added benefit when IVC filters are used in combination with pharmacological thromboprophylaxis. PE still occur despite the presence of a filter. Filters have short- and long-term complication rates and are associated with high cost and often provide a false sense of security, delaying the use of effective pharmacological thromboprophylaxis. Lastly, a further problem with IVC filters is that they require a second invasive procedure to remove.

### IX. Guideline implementation and quality control

#### Guideline implementation

##### Recommendation 38

We recommend the local implementation of evidence-based guidelines for management of the bleeding trauma patient (Grade 1B).

#### Assessment of bleeding control and outcome

##### Recommendation 39

We recommend that local clinical quality and safety management systems include parameters to assess key measures of bleeding control and outcome (Grade 1B).

#### Rationale

Implementation of treatment guidelines in complex areas of clinical care, such as the management of trauma patients, is challenging [6]. However, repetitive educational activities addressing all healthcare providers involved, and in particular training in the simulation centre, have been shown to be successful in increasing guideline adherence [383, 384]. The evaluation of healthcare provider perspectives on guideline quality also plays an important role in a successful implementation process. High guideline credibility, as well as a strong and well-communicated leadership commitment to the guidelines, can increase adherence [383]. Once guidelines are introduced, guideline adherence needs to be monitored with feedback [385] of the results to the involved health care providers.

In addition, clinical debriefing is very useful during the implementation of recommendations to favour clinical decision-making, situational awareness, communication, enhanced teamwork, team leadership and optimisation of resources (space, equipment and environment) [386, 387]. Oral debriefing allows healthcare professionals to anticipate or review medical interventions, emphasise psychological safety and thus provide quality assurance of facilitation strategies and skills with the prevention of cognitive biases and personality traits and their potential impact on decisions, medical errors, behaviour and individual-level patient outcomes [388].

Higher guideline adherence in turn results in improved survival in patients suffering from TBI [389]. This was confirmed in a recent study on the impact of guideline adherence on morbidity and mortality in 882 patients with TBI with a Glasgow Coma Scale score of 4–12 [390]. In this detailed analysis, it could be shown that the more often physiologic parameters were outside the corridors described for goal-directed treatment, the higher the percentage of poor neurologic outcome and mortality [390].

Additionally, in general trauma, adherence to these European guidelines on the management of bleeding trauma patients resulted in higher patient survival [4, 391]. These early and small studies were recently confirmed by an analysis of the treatment and outcome in 1169 severely injured patients treated at the Royal London Hospital Major Trauma Centre from 2008 to 2017 according to their major haemorrhage protocol. During this period, the MT rate dropped from 68 to 24%, median pRBC transfusion from 12 to 4 units and mortality from 45 to 27% [3].

Training in trauma care should emphasise the key role of coagulation in determining outcome. Increasing clinician knowledge and understanding in this area should be an integral part of the implementation of the algorithm. All trauma care centres should evaluate their own performance using a routine institutional quality management programme. An audit of adherence to best practice, including feedback and practice change where needed, should be included as part of the local implementation of these guidelines. To evaluate the quality of care provided to the patient who is bleeding after major trauma, we suggest that adherence to the following quality standards be assessed (Table 4).

**Table 4:** Adherence to the following quality standards may be assessed to evaluate the quality of care provided the bleeding trauma patient

Parameter
Time from injury to the initiation of intervention to stop bleeding (surgery or embolisation) in hypotensive patients who do not respond to initial resuscitation
Time from hospital arrival to availability of a full set of blood results [full blood count, prothrombin time, fibrinogen, calcium, viscoelastic testing (if available)]
Proportion of patients receiving the correct treatment according to the blood results
Proportion of patients receiving tranexamic acid within 3 h after injury
Damage control surgical techniques used in accordance with recommendation R19
Thromboprophylaxis commenced in accordance with recommendation R37

## Discussion

Severe traumatic injury continues to challenge healthcare systems around the world and post-traumatic bleeding remains a leading cause of potentially preventable death among injured patients. Therefore, it is important to provide guidance on the management of major bleeding and coagulopathy following traumatic injury. This sixth edition of the European guideline (summarised in Additional file 3) on the management of major bleeding and coagulopathy following traumatic injury represents an update of the editions published by the same core author group in 2007, 2010, 2013, 2016 and most recently in 2019 [6]. The format of this edition has been adjusted to reflect the trend towards concise guideline documents that cite only the highest-quality studies and most relevant literature rather than attempting to provide a comprehensive literature review to accompany each recommendation. Additional older literature citations and an extended discussion around some of the recommendations included here can therefore be found in previous editions of this guideline.

**Fig. 1:**
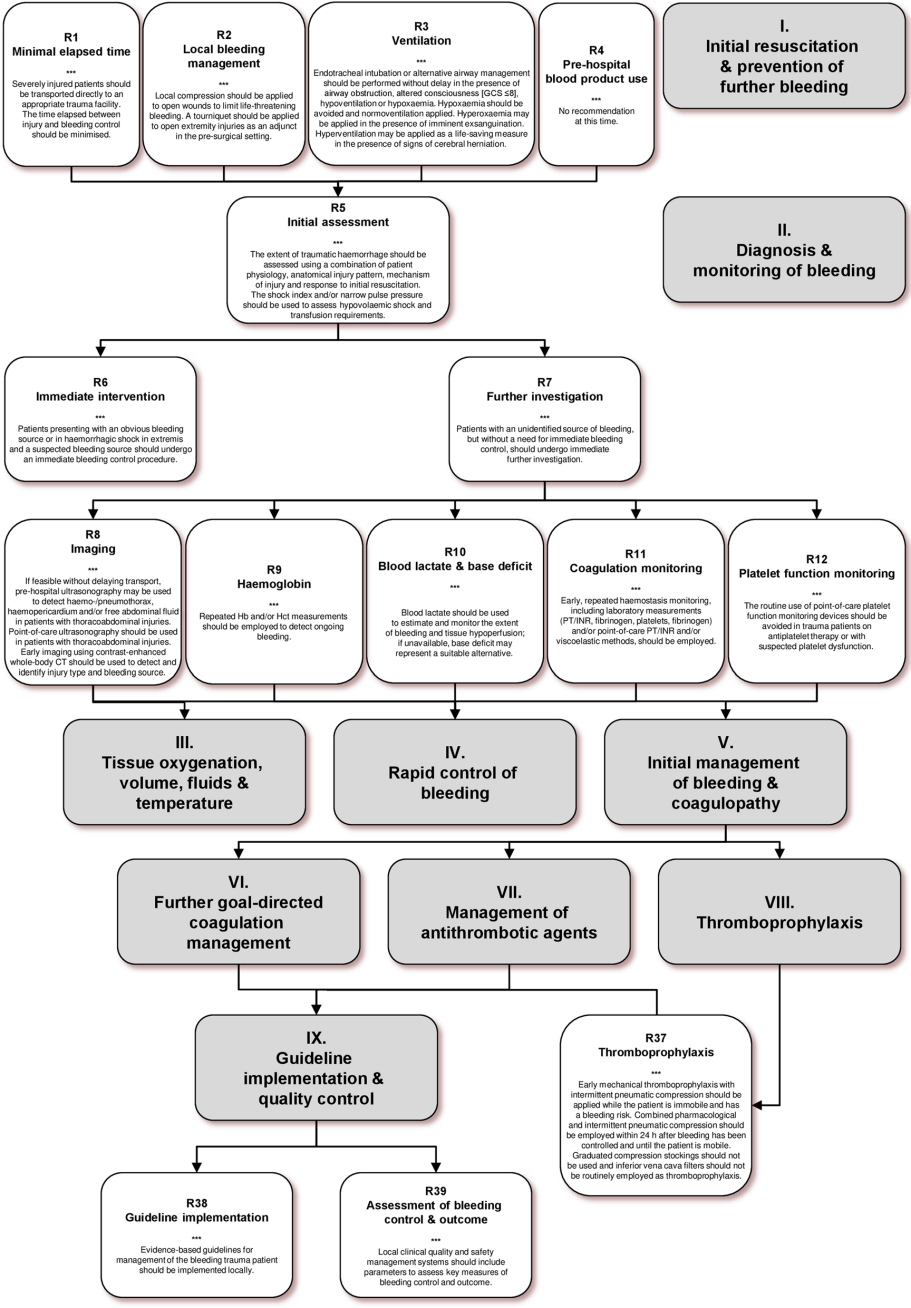

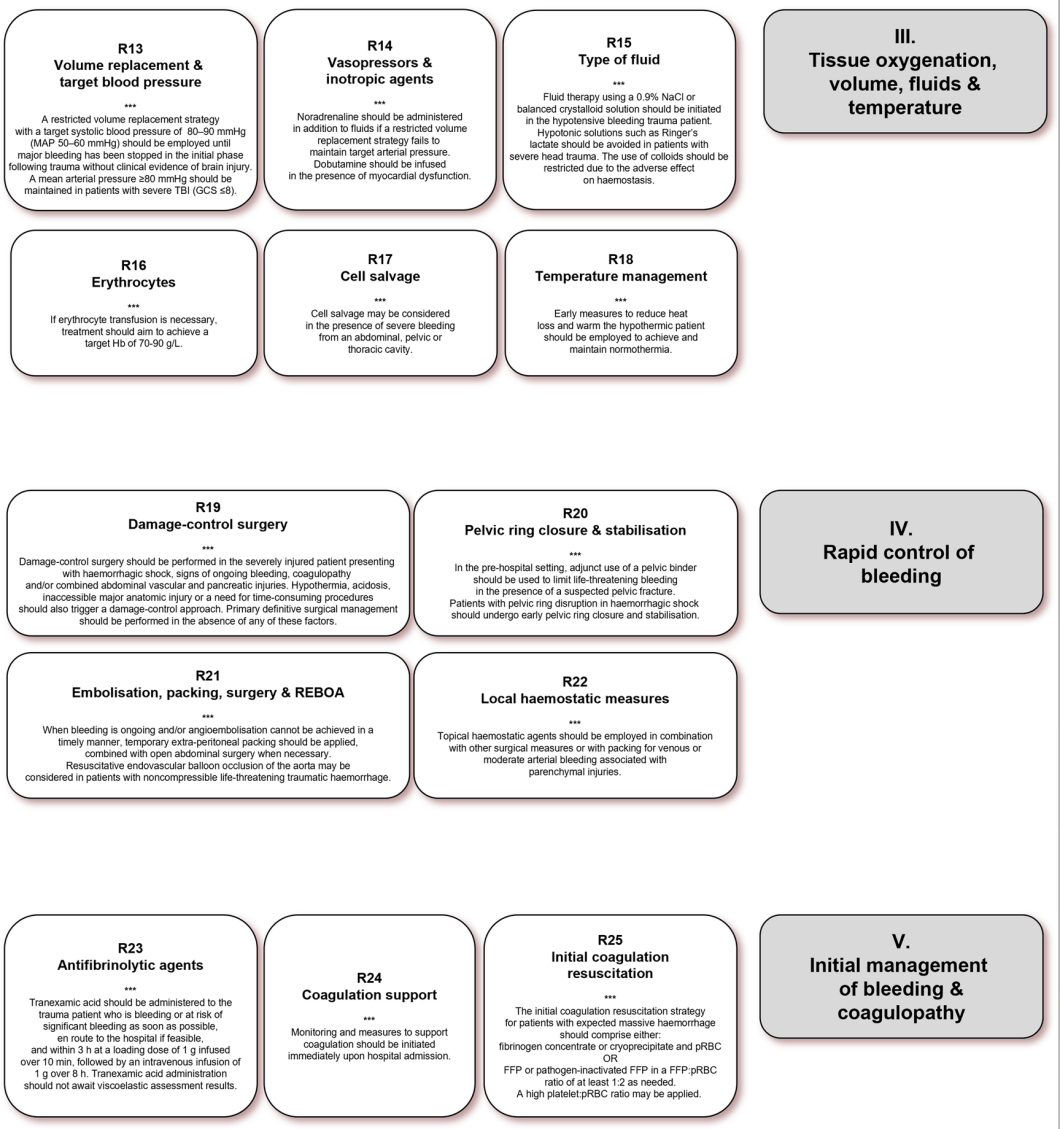

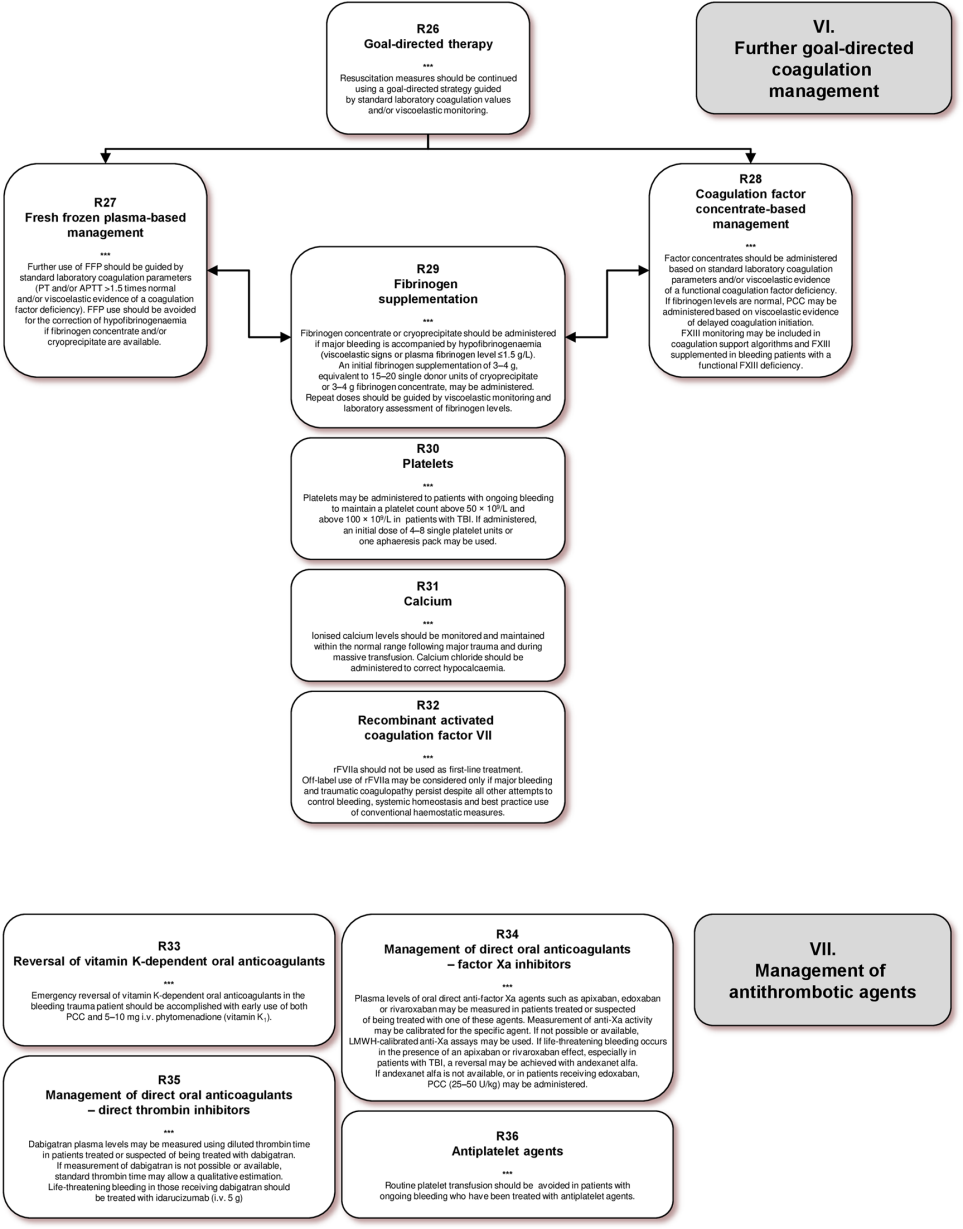
Summary of treatment modalities for the bleeding trauma patient included in this guideline. *APTT* Activated partial thromboplastin time, *CT* Computed tomography, *FFP* Fresh frozen plasma, *GCS* Glasgow coma scale, *Hb* Haemoglobin, *Hct* Haematocrit, *INR* International normalised ratio, *LMWH* Low molecular weight heparin, *MAP* Mean arterial pressure, *PCC* Prothrombin complex concentrate, *PT* Prothrombin time, *pRBC* Packed red blood cells, *REBOA* Resuscitative endovascular balloon occlusion of the aorta, *TBI* Traumatic brain injury

The nine chapters of this guideline continue to follow an approximate temporal path for management of the bleeding trauma patient, with recommendations grouped behind key decision points (Fig. 1; Additional file 4). New to this edition of the guideline is a recommendation and discussion around the use of cell salvage under appropriate circumstances. This edition also discusses the potential pre-hospital use of blood products but does not include a recommendation or suggestion for or against this practice.

This guideline is associated with several limitations. First, only a few of the recommendations are based on high-quality evidence, a fact that highlights the need for future research in this area. Second, in order to support a more general approach to the trauma patient, specific recommendations for special populations such as paediatric patients or patients with TBI have not been included. Third, these guidelines are limited to recommendations for which implementation is likely to be feasible within most European healthcare systems. Nevertheless, we are confident that adherence to these European guidelines for the management of the bleeding trauma patient will result in higher patient survival [4, 5].

In publishing the sixth edition of this guideline, our aim continues to be improvement of outcomes in severely injured trauma patients by optimising and standardising trauma care in line with the available evidence across Europe and beyond.

## Supplementary Information

**Additional file 1.** Summary of PICOs.

**Additional file 2.** Search bundles, structured literature search strategies and results.

**Additional file 3.** Summary of recommendations.

**Additional file 4.** Summary of treatment modalities for the bleeding trauma patient included in this guideline (A0 poster).

## Data Availability

All of the publications cited in this guideline were identified in publicly available databases using the search strategies listed in Additional file 2.

## Abbreviations

AA: Arachidonic acid
ADP: Adenosine diphosphate
AE: Angioembolisation
AIS: Abbreviated injury scale
APA: Antiplatelet agent
APTT: Activated partial thromboplastin time
ARDS: Acute respiratory distress syndrome
ATLS: Advanced trauma life support
AUC: Area under the curve
AUROC: Area under the receiver operating characteristics
CCA: Conventional coagulation assay
CCT: Conventional clotting test
CFC: Coagulation factor concentrate
CPR: Cardiopulmonary resuscitation
CT: Computed tomography
DC: Damage control
DOAC: Direct oral anticoagulant
DVT: Deep vein thrombosis
eFAST: Extended focused assessment with sonography in trauma
EXTEM: Extrinsically activated test with tissue factor
FAST: Focused assessment with sonography in trauma
FFP: Fresh-frozen plasma
FIBTEM: Fibrin-based extrinsically activated test with tissue factor and the platelet inhibitor cytochalasin D
GCS: Glasgow coma scale
GrCS: Graduated compression stockings
Hb: Haemoglobin
Hct: Haematocrit
ICH: Intracranial haemorrhage
ICS: Intraoperative cell salvage
ICU: Intensive care unit
IgE: Immunoglobin E
INR: International normalised ratio
IPC: Intermittent pneumatic compression
ISS: Injury severity score
IVC: Inferior vena cava
LMWH: Low molecular weight heparin
LOS: Length of stay
MAP: Mean arterial pressure
MT: Massive transfusion
OR: Odds ratio
PCC: Prothrombin complex concentrate
PE: Pulmonary embolism
PFT: Platelet function test
PHUS: Pre-hospital ultrasonography
POC: Point-of-care
POCUS: Point-of-care ultrasonography
PP: Pulse pressure
PPP: Preperitoneal pelvic packing
pRBC: Packed red blood cells
PT: Prothrombin time
PTr: Prothrombin time ratio
RCT: Randomised controlled trial
REBOA: Resuscitative endovascular balloon occlusion of the aorta
rFVIIa: Recombinant activated coagulation factor VII
ROTEM: Rotational thromboelastometry
RR: Risk ratio
SI: Shock index
TBI: Traumatic brain injury
TEG: Thromboelastography
TEG-PM: Thromboelastography with platelet mapping
TICH: Traumatic intracranial haemorrhage
TRALI: Transfusion-related acute lung injury
TXA: Tranexamic acid
UFH: Unfractionated heparin
VEM: Viscoelastic monitoring/measures
VKA: Vitamin K antagonist
VTE: Venous thromboembolism
WBCT: Whole-body computed tomography

## References

[1] GBD 2017 Causes of death collaborators: global, regional, and national age-sex-specific mortality for 282 causes of death in 195 countries and territories, 1980–2017: a systematic analysis for the global burden of disease study 2017. Lancet. 2018;392(10159):1736–1788. 10.1016/S0140-6736(18)32203-7 PMC6227606 30496103

[2] GBD 2019 Diseases and injuries collaborators: global burden of 369 diseases and injuries in 204 countries and territories, 1990–2019: a systematic analysis for the global burden of disease study 2019. Lancet. 2020;396(10258):1204–1222. 10.1016/S0140-6736(20)30925-9 PMC7567026 33069326

[3] Cole E, Weaver A, Gall L, West A, Nevin D, Tallach R, O'Neill B, Lahiri S, Allard S, Tai N (2021). A decade of damage control resuscitation: new transfusion practice, new survivors, new directions. Ann Surg.

[4] Godier A, Bacus M, Kipnis E, Tavernier B, Guidat A, Rauch A, Drumez E, Susen S, Garrigue-Huet D (2016). Compliance with evidence-based clinical management guidelines in bleeding trauma patients. Br J Anaesth.

[5] Stein P, Kaserer A, Sprengel K, Wanner GA, Seifert B, Theusinger OM, Spahn DR (2017). Change of transfusion and treatment paradigm in major trauma patients. Anaesthesia.

[6] Spahn DR, Bouillon B, Cerny V, Duranteau J, Filipescu D, Hunt BJ, Komadina R, Maegele M, Nardi G, Riddez L (2019). The European guideline on management of major bleeding and coagulopathy following trauma: fifth edition. Crit Care.

[7] Guyatt G, Gutterman D, Baumann MH, Addrizzo-Harris D, Hylek EM, Phillips B, Raskob G, Lewis SZ, Schünemann H (2006). Grading strength of recommendations and quality of evidence in clinical guidelines: report from an American College of Chest Physicians Task Force. Chest.

[8] Higgins JP, Altman DG, Gotzsche PC, Juni P, Moher D, Oxman AD, Savovic J, Schulz KF, Weeks L, Sterne JA (2011). The Cochrane collaboration's tool for assessing risk of bias in randomised trials. BMJ.

[9] Alharbi RJ, Lewis V, Shrestha S, Miller C (2021). Effectiveness of trauma care systems at different stages of development in reducing mortality: a systematic review and meta-analysis protocol. BMJ Open.

[10] Dufresne P, Moore L, Tardif P, Razek T, Omar M, Boutin A, Clément J (2017). Impact of trauma centre designation level on outcomes following haemorrhagic shock: a multicentre cohort study. Can J Surg.

[11] Kalkwarf KJ, Drake SA, Yang Y, Thetford C, Myers L, Brock M, Wolf DA, Persse D, Wade CE, Holcomb JB (2020). Bleeding to death in a big city: an analysis of all trauma deaths from haemorrhage in a metropolitan area during 1 year. J Trauma Acute Care Surg.

[12] Ruelas OS, Tschautscher CF, Lohse CM, Sztajnkrycer MD (2018). Analysis of prehospital scene times and interventions on mortality outcomes in a national cohort of penetrating and blunt trauma patients. Prehosp Emerg Care.

[13] Nasser AAH, Nederpelt C, El Hechi M, Mendoza A, Saillant N, Fagenholz P, Velmahos G, Kaafarani HMA (2020). Every minute counts: the impact of pre-hospital response time and scene time on mortality of penetrating trauma patients. Am J Surg.

[14] Harmsen AMK, Giannakopoulos GF, Moerbeek PR, Jansma EP, Bonjer HJ, Bloemers FW (2015). The influence of prehospital time on trauma patients outcome: a systematic review. Injury.

[15] Chen C, Shin SD, Sun J, Jamaluddin SF, Tanaka H, Song KJ, Kajino K, Kimura A, Huang EP, Hsieh M (2020). Association between prehospital time and outcome of trauma patients in 4 Asian countries: a cross-national, multicentre cohort study. PLOS Med.

[16] Chehab M, Afaneh A, Bible L, Castanon L, Hanna K, Ditillo M, Khurrum M, Asmar S, Joseph B (2020). Angioembolization in intra-abdominal solid organ injury: Does delay in angioembolization affect outcomes?. J Trauma Acute Care Surg.

[17] Matsushima K, Khor D, Berona K, Antoku D, Dollbaum R, Khan M, Demetriades D (2018). Double jeopardy in penetrating trauma: get FAST, get it right. World J Surg.

[18] Singh RA, Asprou F, Patel A, Trickett RW. Haemorrhage control in extremity stab injury. J Surg Case Rep 2013;2013(12):rjt093. 10.1093/jscr/rjt093 PMC3854963 24968429

[19] Van Waes OJ, Cheriex KCAL, Navsaria PH, van Riet PA, Nicol AJ, Vermeulen J (2012). Management of penetrating neck injuries. Br J Surg.

[20] Bulger EM, Snyder D, Schoelles K, Gotschall C, Dawson D, Lang E, Sanddal ND, Butler FK, Fallat M, Taillac P (2014). An evidence-based prehospital guideline for external haemorrhage control: American College of Surgeons Committee on Trauma. Prehosp Emerg Care.

[21] Lakstein D, Blumenfeld A, Sokolov T, Lin G, Bssorai R, Lynn M, Ben-Abraham R (2003). Tourniquets for haemorrhage control on the battlefield: a 4-year accumulated experience. J Trauma.

[22] Beekley AC, Sebesta JA, Blackbourne LH, Herbert GS, Kauvar DS, Baer DG, Walters TJ, Mullenix PS, Holcomb JB (2008). Prehospital tourniquet use in operation Iraqi freedom: effect on heamorrhage control and outcomes. J Trauma.

[23] Kragh JF, Cooper A, Aden JK, Dubick MA, Baer DG, Wade CE, Blackbourne LH (2012). Survey of trauma registry data on tourniquet use in paediatric war casualties. Pediatr Emerg Care.

[24] Smith AA, Ochoa JE, Wong S, Beatty S, Elder J, Guidry C, McGrew P, McGinness C, Duchesne J, Schroll R (2019). Prehospital tourniquet use in penetrating extremity trauma: decreased blood transfusions and limb complications. J Trauma Acute Care Surg.

[25] Eilertsen KA, Winberg M, Jeppesen E, Hval G, Wisborg T (2021). Prehospital tourniquets in civilians: a systematic review. Prehosp Disaster Med.

[26] Dayan L, Zinmann C, Stahl S, Norman D (2008). Complications associated with prolonged tourniquet application on the battlefield. Mil Med.

[27] Kragh JF, O'Neill ML, Walters TJ, Jones JA, Baer DG, Gershman LK, Wade CE, Holcomb JB (2011). Minor morbidity with emergency tourniquet use to stop bleeding in severe limb trauma: research, history, and reconciling advocates and abolitionists. Mil Med.

[28] Bernard SA, Nguyen V, Cameron P, Masci K, Fitzgerald M, Cooper DJ, Walker T, Myles P, Murray L (2010). Prehospital rapid sequence intubation improves functional outcome for patients with severe traumatic brain injury: a randomised controlled trial. Ann Surg.

[29] Bukur M, Kurtovic S, Berry C, Tanios M, Margulies DR, Ley EJ, Salim A (2011). Pre-hospital intubation is associated with increased mortality after traumatic brain injury. J Surg Res.

[30] Lee A, Chien Y, Lee B, Yang W, Wang Y, Lin H, Huang EP, Chong K, Sun J, Huei-Ming M (2022). Effect of placement of a supraglottic airway device vs endotracheal intubation on return of spontaneous circulation in adults with out-of-hospital cardiac arrest in Taipei, Taiwan: a cluster randomised clinical trial. JAMA Netw Open.

[31] Boer C, Franschman G, Loer SA (2012). Prehospital management of severe traumatic brain injury: concepts and ongoing controversies. Curr Opin Anaesthesiol.

[32] Singer M, Young PJ, Laffey JG, Asfar P, Taccone FS, Skrifvars MB, Meyhoff CS, Radermacher P (2021). Dangers of hyperoxia. Crit Care.

[33] Chu DK, Kim LH, Young PJ, Zamiri N, Almenawer SA, Jaeschke R, Szczeklik W, Schünemann HJ, Neary JD, Alhazzani W (2018). Mortality and morbidity in acutely ill adults treated with liberal versus conservative oxygen therapy (IOTA): a systematic review and meta-analysis. Lancet.

[34] Damiani E, Adrario E, Girardis M, Romano R, Pelaia P, Singer M, Donati A (2014). Arterial hyperoxia and mortality in critically ill patients: a systematic review and meta-analysis. Crit Care.

[35] Page D, Ablordeppey E, Wessman BT, Mohr NM, Trzeciak S, Kollef MH, Roberts BW, Fuller BM (2018). Emergency department hyperoxia is associated with increased mortality in mechanically ventilated patients: a cohort study. Crit Care.

[36] Vincent J, Taccone FS, He X (2017). Harmful effects of hyperoxia in postcardiac arrest, sepsis, traumatic brain injury, or stroke: the importance of individualised oxygen therapy in critically ill patients. Can Respir J.

[37] Brugniaux JV, Coombs GB, Barak OF, Dujic Z, Sekhon MS, Ainslie PN (2018). Highs and lows of hyperoxia: physiological, performance, and clinical aspects. Am J Physiol Regul Integr Comp Physiol.

[38] Smit B, Smulders YM, van der Wouden JC, Oudemans-van Straaten HM, Spoelstra-de Man AME (2018). Haemodynamic effects of acute hyperoxia: systematic review and meta-analysis. Crit Care.

[39] Davis DP, Idris AH, Sise MJ, Kennedy F, Eastman AB, Velky T, Vilke GM, Hoyt DB (2006). Early ventilation and outcome in patients with moderate to severe traumatic brain injury. Crit Care Med.

[40] Sperry JL, Guyette FX, Brown JB, Yazer MH, Triulzi DJ, Early-Young BJ, Adams PW, Daley BJ, Miller RS, Harbrecht BG (2018). Prehospital plasma during air medical transport in trauma patients at risk for haemorrhagic shock. N Engl J Med.

[41] Moore HB, Moore EE, Chapman MP, McVaney K, Bryskiewicz G, Blechar R, Chin T, Burlew CC, Pieracci F, West FB (2018). Plasma-first resuscitation to treat haemorrhagic shock during emergency ground transportation in an urban area: a randomised trial. Lancet.

[42] Reitz KM, Moore HB, Guyette FX, Sauaia A, Pusateri AE, Moore EE, Hassoune A, Chapman MP, Daley BJ, Miller RS (2020). Prehospital plasma in injured patients is associated with survival principally in blunt injury: results from two randomised prehospital plasma trials. J Trauma Acute Care Surg.

[43] Gruen DS, Guyette FX, Brown JB, Okonkwo DO, Puccio AM, Campwala IK, Tessmer MT, Daley BJ, Miller RS, Harbrecht BG (2020). Association of prehospital plasma with survival in patients with traumatic brain injury: a secondary analysis of the PAMPer cluster randomised clinical trial. JAMA Netw Open.

[44] Pusateri AE, Moore EE, Moore HB, Le TD, Guyette FX, Chapman MP, Sauaia A, Ghasabyan A, Chandler J, McVaney K (2020). Association of prehospital plasma transfusion with survival in trauma patients with haemorrhagic shock when transport times are longer than 20 minutes: a post hoc analysis of the PAMPer and COMBAT clinical trials. JAMA Surg.

[45] Coccolini F, Pizzilli G, Corbella D, Sartelli M, Agnoletti V, Agostini V, Baiocchi GL, Ansaloni L, Catena F (2019). Pre-hospital plasma in haemorrhagic shock management: current opinion and meta-analysis of randomised trials. World J Emerg Surg.

[46] Rehn M, Weaver A, Brohi K, Eshelby S, Green L, Røislien J, Lockey DJ (2019). Effect of prehospital red blood cell transfusion on mortality and time of death in civilian trauma patients. Shock.

[47] Rijnhout TWH, Wever KE, Marinus RHAR, Hoogerwerf N, Geeraedts LMG, Tan ECTH (2019). Is prehospital blood transfusion effective and safe in haemorrhagic trauma patients? A systematic review and meta-analysis. Injury.

[48] van Turenhout EC, Bossers SM, Loer SA, Giannakopoulos GF, Schwarte LA, Schober P (2020). Pre-hospital transfusion of red blood cells. Part 2: a systematic review of treatment effects on outcomes. Transfus Med.

[49] Guyette FX, Sperry JL, Peitzman AB, Billiar TR, Daley BJ, Miller RS, Harbrecht BG, Claridge JA, Putnam T, Duane TM (2021). Prehospital blood product and crystalloid resuscitation in the severely injured patient: a secondary analysis of the prehospital air medical plasma trial. Ann Surg.

[50] Shlaifer A, Siman-Tov M, Radomislensky I, Peleg K, Klein Y, Glassberg E, Yitzhak A (2019). The impact of prehospital administration of freeze-dried plasma on casualty outcome. J Trauma Acute Care Surg.

[51] Oakeshott JE, Griggs JE, Wareham GM, Lyon RM (2019). Kent surrey sussex air ambulance trust: feasibility of prehospital freeze-dried plasma administration in a UK helicopter emergency medical service. Eur J Emerg Med.

[52] Crombie N, Doughty HA, Bishop JRB, Desai A, Dixon EF, Hancox JM, Herbert MJ, Leech C, Lewis SJ, Nash MR (2022). Resuscitation with blood products in patients with trauma-related haemorrhagic shock receiving prehospital care (RePHILL): a multicentre, open-label, randomised, controlled, phase 3 trial. Lancet Haematol.

[53] American College of Surgeons: ATLS^®^-advanced trauma life support student course manual, 10th ed. Chicago, IL: American College of Surgeons; 2018. p. 60611–3211.

[54] Mutschler M, Nienaber U, Brockamp T, Wafaisade A, Wyen H, Peiniger S, Paffrath T, Bouillon B, Maegele M (2013). A critical reappraisal of the ATLS classification of hypovolaemic shock: does it really reflect clinical reality?. Resuscitation.

[55] Tran A, Matar M, Lampron J, Steyerberg E, Taljaard M, Vaillancourt C (2018). Early identification of patients requiring massive transfusion, embolization or haemostatic surgery for traumatic haemorrhage: a systematic review and meta-analysis. J Trauma Acute Care Surg.

[56] Liu C, Wang C, Shih H, Wen Y, Wu JJ, Huang C, Hsu H, Huang M, Huang M (2009). Prognostic factors for mortality following falls from height. Injury.

[57] Nutbeam T, Fenwick R, Smith J, Bouamra O, Wallis L, Stassen W (2021). A comparison of the demographics, injury patterns and outcome data for patients injured in motor vehicle collisions who are trapped compared to those patients who are not trapped. Scand J Trauma Resusc Emerg Med.

[58] Vandromme MJ, Griffin RL, Kerby JD, McGwin G, Rue LW, Weinberg JA (2011). Identifying risk for massive transfusion in the relatively normotensive patient: utility of the prehospital shock index. J Trauma.

[59] Campos-Serra A, Montmany-Vioque S, Rebasa-Cladera P, Llaquet-Bayo H, Gràcia-Roman R, Colom-Gordillo A, Navarro-Soto S (2018). The use of the shock index as a predictor of active bleeding in trauma patients. Cir Esp (Engl Ed).

[60] Schroll R, Swift D, Tatum D, Couch S, Heaney JB, Llado-Farrulla M, Zucker S, Gill F, Brown G, Buffin N (2018). Accuracy of shock index versus ABC score to predict need for massive transfusion in trauma patients. Injury.

[61] Terceros-Almanza LJ, García-Fuentes C, Bermejo-Aznárez S, Prieto Del Portillo IJ, Mudarra-Reche C, Domínguez-Aguado H, Viejo-Moreno R, Barea-Mendoza J, Gómez-Soler R, Casado-Flores I (2019). Prediction of massive bleeding in a prehospital setting: validation of six scoring systems. Med Intensiva (Engl Ed).

[62] Sorensen DA, April MD, Fisher AD, Schauer SG. An analysis of the shock index and pulse pressure as a predictor for massive transfusion and death in US and coalition Iraq and Afghanistan. Med J (Ft Sam Houst Tex) 2021(Pb 8-21-07/08/09):63–68. 34449863

[63] El-Menyar A, Goyal P, Tilley E, Latifi R (2018). The clinical utility of shock index to predict the need for blood transfusion and outcomes in trauma. J Surg Res.

[64] Kheirbek T, Martin TJ, Cao J, Hall BM, Lueckel S, Adams CA (2021). Prehospital shock index outperforms hypotension alone in predicting significant injury in trauma patients. Trauma Surg Acute Care Open.

[65] Priestley EM, Inaba K, Byerly S, Biswas S, Wong MD, Lam L, Benjamin E, Demetriades D (2019). Pulse pressure as an early warning of haemorrhage in trauma patients. J Am Coll Surg.

[66] Bankhead-Kendall B, Teixeira P, Roward S, Ali S, Ryder A, Sahi S, Cardenas T, Aydelotte J, Coopwood B, Brown C (2020). Narrow pulse pressure is independently associated with massive transfusion and emergent surgery in haemodynamically stable trauma patients. Am J Surg.

[67] Schellenberg M, Owattanapanich N, Getrajdman J, Matsushima K, Inaba K (2021). Prehospital narrow pulse pressure predicts need for resuscitative thoracotomy and emergent intervention after trauma. J Surg Res.

[68] Warren J, Moazzez A, Chong V, Putnam B, Neville A, Singer G, Deane M, Kim DY (2019). Narrowed pulse pressure predicts massive transfusion and emergent operative intervention following penetrating trauma. Am J Surg.

[69] Johnson JW, Gracias VH, Schwab CW, Reilly PM, Kauder DR, Shapiro MB, Dabrowski GP, Rotondo MF (2001). Evolution in damage control for exsanguinating penetrating abdominal injury. J Trauma.

[70] Martin M, Izenberg S, Cole F, Bergstrom S, Long W (2012). A decade of experience with a selective policy for direct to operating room trauma resuscitations. Am J Surg.

[71] Wieck MM, Cunningham AJ, Behrens B, Ohm ET, Maxwell BG, Hamilton NA, Adams MC, Cole FJ, Jafri MA (2018). Direct to operating room trauma resuscitation decreases mortality among severely injured children. J Trauma Acute Care Surg.

[72] Johnson A, Rott M, Kuchler A, Williams E, Cole F, Ramzy A, Barbosa R, Long W, Martin MJ (2020). Direct to operating room trauma resuscitation: optimizing patient selection and time-critical outcomes when minutes count. J Trauma Acute Care Surg.

[73] Kim H, Jeon CH, Kim JH, Kwon H, Kim CW, Kim GH, Lee CK, Lee SB, Jang JH, Kim SH et al. Relationship between door-to-embolization time and clinical outcomes after transarterial embolization in trauma patients with complex pelvic fracture. Eur J Trauma Emerg Surg. 2021. Preprint. https://www.ncbi.nlm.nih.gov/pubmed/33523237. 10.1007/s00068-021-01601-7 PMC9192384 33523237

[74] Kinoshita T, Yamakawa K, Matsuda H, Yoshikawa Y, Wada D, Hamasaki T, Ono K, Nakamori Y, Fujimi S (2019). The survival benefit of a novel trauma workflow that includes immediate whole-body computed tomography, surgery, and interventional radiology, all in one trauma resuscitation room: a retrospective historical control study. Ann Surg.

[75] Huber-Wagner S, Lefering R, Qvick LM, Körner M, Kay MV, Pfeifer KJ, Reiser M, Mutschler W, Kanz KG (2009). Effect of whole-body CT during trauma resuscitation on survival: a retrospective, multicentre study. Lancet.

[76] Linsenmaier U, Krötz M, Häuser H, Rock C, Rieger J, Bohndorf K, Pfeifer KJ, Reiser M (2002). Whole-body computed tomography in polytrauma: techniques and management. Eur Radiol.

[77] Albrecht T, von Schlippenbach J, Stahel PF, Ertel W, Wolf K (2004). The role of whole body spiral CT in the primary work-up of polytrauma patients - comparison with conventional radiography and abdominal sonography. Rofo.

[78] Huber-Wagner S, Mand C, Ruchholtz S, Kühne CA, Holzapfel K, Kanz K, van Griensven M, Biberthaler P, Lefering R (2014). Effect of the localisation of the CT scanner during trauma resuscitation on survival - a retrospective, multicentre study. Injury.

[79] Huber-Wagner S, Biberthaler P, Häberle S, Wierer M, Dobritz M, Rummeny E, van Griensven M, Kanz KG, Lefering R (2013). Whole-body CT in haemodynamically unstable severely injured patients—a retrospective, multicentre study. PLoS ONE.

[80] Suda AJ, Baran K, Brunnemer S, Köck M, Obertacke U, Eschmann D (2022). Delayed diagnosed trauma in severely injured patients despite guidelines-oriented emergency room treatment: there is still a risk. Eur J Trauma Emerg Surg.

[81] HonShideler C, Bernal-Fernandez M, Hsu M, Shin D, Haran P, Soto J, Anderson S, Ramalingam V (2021). Clinical and laboratory parameters in blunt pelvic trauma not associated with subsequent positive conventional angiography in patients with positive CTA. Emerg Radiol.

[82] Lai Y, Wu C, Chen H, Wang L, Wong Y (2018). Predictors of active arterial haemorrhage on angiography in pelvic fracture patients. Jpn J Radiol.

[83] Birkl J, Kahl T, Thielemann H, Mutze S, Goelz L (2020). Retrospective analysis and systematic review of isolated traumatic dissections of the celiac artery. Ann Vasc Surg.

[84] van der Weide L, Popal Z, Terra M, Schwarte LA, Ket JCF, Kooij FO, Exadaktylos AK, Zuidema WP, Giannakopoulos GF (2019). Prehospital ultrasound in the management of trauma patients: systematic review of the literature. Injury.

[85] Mercer CB, Ball M, Cash RE, Rivard MK, Chrzan K, Panchal AR (2021). Ultrasound use in the prehospital setting for trauma: a systematic review. Prehosp Emerg Care.

[86] Stengel D, Leisterer J, Ferrada P, Ekkernkamp A, Mutze S, Hoenning A (2018). Point-of-care ultrasonography for diagnosing thoracoabdominal injuries in patients with blunt trauma. Cochrane Database Syst Rev.

[87] Rowell SE, Barbosa RR, Holcomb JB, Fox EE, Barton CA, Schreiber MA (2019). The focused assessment with sonography in trauma (FAST) in hypotensive injured patients frequently fails to identify the need for laparotomy: a multi-institutional pragmatic study. Trauma Surg Acute Care Open.

[88] Staub LJ, Biscaro RRM, Kaszubowski E, Maurici R (2018). Chest ultrasonography for the emergency diagnosis of traumatic pneumothorax and haemothorax: a systematic review and meta-analysis. Injury.

[89] Gonzalez-Hadad A, García AF, Serna JJ, Herrera MA, Morales M, Manzano-Nunez R (2020). The role of ultrasound for detecting occult penetrating cardiac wounds in haemodynamically stable patients. World J Surg.

[90] Ianniello S, Conte P, Di Serafino M, Miele V, Trinci M, Vallone G, Galluzzo M (2021). Diagnostic accuracy of pubic symphysis ultrasound in the detection of unstable pelvis in polytrauma patients during e-FAST: the value of FAST-PLUS protocol. A preliminary experience. J Ultrasound.

[91] Pigneri DA, Behm RJ, Granet PJ (2020). Rolling a trauma patient onto the right side increases sensitivity of FAST examination. J Clin Ultrasound.

[92] Hajibandeh S, Hajibandeh S (2015). Systematic review: effect of whole-body computed tomography on mortality in trauma patients. J Inj Violence Res.

[93] Caleo O, Bocchini G, Paoletta S, Ierardi AM, Scionti A, Tonerini M, Guida F, Sica G, Perillo A, Carrafiello G (2015). Spontaneous non-aortic retroperitoneal haemorrhage: etiology, imaging characterization and impact of MDCT on management. A multicentric study. Radiol Med.

[94] Shreffler J, Smiley A, Schultz M, Ross A, Baker J, Nash N, Harbrecht B, Huecker M (2020). Patients with abrasion or ecchymosis seat belt sign have high risk for abdominal injury, but initial computed tomography is 100% sensitive. J Emerg Med.

[95] Sierink JC, Treskes K, Edwards MJ, Beuker BJ, den Hartog D, Hohmann J, Dijkgraaf MG, Luitse JS, Beenen LF, Hollmann MW (2016). Immediate total-body CT scanning versus conventional imaging and selective CT scanning in patients with severe trauma (REACT-2): a randomised controlled trial. Lancet.

[96] Treskes K, Saltzherr TP, Edwards MJR, Beuker BJA, Den Hartog D, Hohmann J, Luitse JS, Beenen LFM, Hollmann MW, Dijkgraaf MGW (2019). Emergency bleeding control interventions after immediate total-body CT scans in trauma patients. World J Surg.

[97] Arruzza E, Chau M, Dizon J (2020). Systematic review and meta-analysis of whole-body computed tomography compared to conventional radiological procedures of trauma patients. Eur J Radiol.

[98] Murao S, Yamakawa K, Kabata D, Kinoshita T, Umemura Y, Shintani A, Fujimi S (2021). Effect of earlier door-to-CT and door-to-bleeding control in severe blunt trauma: a retrospective cohort study. J Clin Med.

[99] Treskes K, Saltzherr TP, Edwards MJR, Beuker BJA, Van Lieshout EMM, Hohmann J, Luitse JSK, Beenen LFM, Hollmann MW, Dijkgraaf MGW (2020). Refining the criteria for immediate total-body CT after severe trauma. Eur Radiol.

[100] Cieslak JA, Jazmati T, Patel A, Chaudhry H, Kumar A, Contractor S, Shukla PA (2020). Trauma CT evaluation prior to selective angiography in patients with traumatic injuries: negative predictive power and factors affecting its utility. Emerg Radiol.

[101] Gamal M, Abdelhamid B, Zakaria D, Dayem OAE, Rady A, Fawzy M, Hasanin A (2018). Evaluation of noninvasive haemoglobin monitoring in trauma patients with low haemoglobin levels. Shock.

[102] Kass LE, Tien IY, Ushkow BS, Snyder HS (1997). Prospective crossover study of the effect of phlebotomy and intravenous crystalloid on haematocrit. Acad Emerg Med.

[103] Ryan ML, Thorson CM, Otero CA, Vu T, Schulman CI, Livingstone AS, Proctor KG (2012). Initial haematocrit in trauma: A paradigm shift?. J Trauma Acute Care Surg.

[104] Knottenbelt JD (1991). Low initial haemoglobin levels in trauma patients: an important indicator of ongoing haemorrhage. J Trauma.

[105] Thorson CM, Van Haren RM, Ryan ML, Pereira R, Olloqui J, Guarch GA, Barrera JM, Busko AM, Livingstone AS, Proctor KG (2013). Admission haematocrit and transfusion requirements after trauma. J Am Coll Surg.

[106] Thorson CM, Ryan ML, Van Haren RM, Pereira R, Olloqui J, Otero CA, Schulman CI, Livingstone AS, Proctor KG (2013). Change in haematocrit during trauma assessment predicts bleeding even with ongoing fluid resuscitation. Am Surg.

[107] Zehtabchi S, Sinert R, Goldman M, Kapitanyan R, Ballas J (2006). Diagnostic performance of serial haematocrit measurements in identifying major injury in adult trauma patients. Injury.

[108] Holstein JH, Culemann U, Pohlemann T (2012). Working group mortality in pelvic fracture patients: What are predictors of mortality in patients with pelvic fractures?. Clin Orthop Relat Res.

[109] Schlimp CJ, Voelckel W, Inaba K, Maegele M, Ponschab M, Schöchl H (2013). Estimation of plasma fibrinogen levels based on haemoglobin, base excess and injury severity score upon emergency room admission. Crit Care.

[110] Figueiredo S, Taconet C, Harrois A, Hamada S, Gauss T, Raux M, Duranteau J (2018). Traumabase group: How useful are haemoglobin concentration and its variations to predict significant haemorrhage in the early phase of trauma? A multicentric cohort study. Ann Intensive Care.

[111] Broder G, Weil MH (1964). Excess lactate: an index of reversibility of shock in human patients. Science.

[112] Caputo N, Fraser R, Paliga A, Kanter M, Hosford K, Madlinger R (2013). Triage vital signs do not correlate with serum lactate or base deficit, and are less predictive of operative intervention in penetrating trauma patients: a prospective cohort study. Emerg Med J.

[113] Vincent JL, Quintairos e Silva A, Couto L, Taccone FS (2016). The value of blood lactate kinetics in critically ill patients: a systematic review. Crit Care.

[114] Gustafson ML, Hollosi S, Tomanguillo Chumbe J, Samanta D, Modak A, Bethea A (2015). The effect of ethanol on lactate and base deficit as predictors of morbidity and mortality in trauma. Am J Emerg Med.

[115] Mutschler M, Nienaber U, Brockamp T, Wafaisade A, Fabian T, Paffrath T, Bouillon B, Maegele M, TraumaRegister DGU (2013). Renaissance of base deficit for the initial assessment of trauma patients: a base deficit-based classification for hypovolemic shock developed on data from 16,305 patients derived from the TraumaRegister DGU®. Crit Care.

[116] Randolph LC, Takacs M, Davis KA (2002). Resuscitation in the pediatric trauma population: admission base deficit remains an important prognostic indicator. J Trauma.

[117] Porter JM, Ivatury RR (1998). In search of the optimal end points of resuscitation in trauma patients: a review. J Trauma.

[118] Wilson M, Davis DP, Coimbra R (2003). Diagnosis and monitoring of haemorrhagic shock during the initial resuscitation of multiple trauma patients: a review. J Emerg Med.

[119] Jiang RM, Pourzanjani AA, Cohen MJ, Petzold L (2021). Associations of longitudinal D-dimer and Factor II on early trauma survival risk. BMC Bioinformatics.

[120] Ishii K, Kinoshita T, Kiridume K, Watanabe A, Yamakawa K, Nakao S, Fujimi S, Matsuoka T (2019). Impact of initial coagulation and fibrinolytic markers on mortality in patients with severe blunt trauma: a multicentre retrospective observational study. Scand J Trauma Resusc Emerg Med.

[121] Deras P, Nouri J, Martinez O, Aubry E, Capdevila X, Charbit J (2018). Diagnostic performance of prothrombin time point-of-care to detect acute traumatic coagulopathy on admission: experience of 522 cases in trauma centre. Transfusion.

[122] Gauss T, Hamada S, Jurcisin I, Dahmani S, Boudaoud L, Mantz J, Paugam-Burtz C (2014). Limits of agreement between measures obtained from standard laboratory and the point-of-care device hemochron signature elite (R) during acute haemorrhage. Br J Anaesth.

[123] Davenport R, Manson J, De'Ath H, Platton S, Coates A, Allard S, Hart D, Pearse R, Pasi KJ, MacCallum P (2011). Functional definition and characterisation of acute traumatic coagulopathy. Crit Care Med.

[124] Baksaas-Aasen K, Van Dieren S, Balvers K, Juffermans NP, Naess PA, Rourke C, Eaglestone S, Ostrowski SR, Stensballe J, Stanworth S (2019). Data-driven development of ROTEM and TEG algorithms for the management of trauma haemorrhage: a prospective observational multicentre study. Ann Surg.

[125] Baksaas-Aasen K, Gall LS, Stensballe J, Juffermans NP, Curry N, Maegele M, Brooks A, Rourke C, Gillespie S, Murphy J (2021). Viscoelastic haemostatic assay augmented protocols for major trauma haemorrhage (ITACTIC): a randomised, controlled trial. Intensive Care Med.

[126] Samuels JM, Moore EE, Silliman CC, Banerjee A, Cohen MJ, Ghasabyan A, Chandler J, Coleman JR, Sauaia A (2019). Severe traumatic brain injury is associated with a unique coagulopathy phenotype. J Trauma Acute Care Surg.

[127] Cannon JW, Dias JD, Kumar MA, Walsh M, Thomas SG, Cotton BA, Schuster JM, Evans SL, Schreiber MA, Adam EH (2021). Use of thromboelastography in the evaluation and management of patients with traumatic brain injury: a systematic review and meta-analysis. Crit Care Explor.

[128] Shammassian BH, Ronald A, Smith A, Sajatovic M, Mangat HS, Kelly ML (2022). Viscoelastic haemostatic assays and outcomes in traumatic brain injury: a systematic literature review. World Neurosurg.

[129] Neal MD, Moore EE, Walsh M, Thomas S, Callcut RA, Kornblith LZ, Schreiber M, Ekeh AP, Singer AJ, Lottenberg L (2020). A comparison between the TEG 6s and TEG 5000 analysers to assess coagulation in trauma patients. J Trauma Acute Care Surg.

[130] Choi PA, Parry PV, Bauer JS, Zusman BE, Panczykowski DM, Puccio AM, Okonkwo DO (2017). Use of aspirin and P2Y12 response assays in detecting reversal of platelet inhibition with platelet transfusion in patients with traumatic brain injury on antiplatelet therapy. Neurosurgery.

[131] Lindblad C, Thelin EP, Nekludov M, Frostell A, Nelson DW, Svensson M, Bellander BM (2018). Assessment of platelet function in traumatic brain injury-a retrospective observational study in the neuro-critical care setting. Front Neurol.

[132] Barton CA, Oetken HJ, Roberti GJ, Dewey EN, Goodman A, Schreiber M (2021). Thromboelastography with platelet mapping: limited predictive ability in detecting preinjury antiplatelet agent use. J Trauma Acute Care Surg.

[133] Eastman DK, Spilman SK, Tang K, Sidwell RA, Pelaez CA (2021). Platelet reactivity testing for aspirin patients who sustain traumatic intracranial haemorrhage. J Surg Res.

[134] Connelly CR, Yonge JD, McCully SP, Hart KD, Hilliard TC, Lape DE, Watson JJ, Rick B, Houser B, Deloughery TG (2017). Assessment of three point-of-care platelet function assays in adult trauma patients. J Surg Res.

[135] Sirajuddin S, Valdez C, DePalma L, Maluso P, Singhal R, Schroeder M, Sarani B (2016). Inhibition of platelet function is common following even minor injury. J Trauma Acute Care Surg.

[136] Alvikas J, Zenati M, Campwala I, Jansen JO, Hassoune A, Phelos H, Okonkwo DO, Neal MD (2022). Rapid detection of platelet inhibition and dysfunction in traumatic brain injury: a prospective observational study. J Trauma Acute Care Surg.

[137] Guillotte AR, Herbert JP, Madsen R, Hammer RD, Litofsky NS (2018). Effects of platelet dysfunction and platelet transfusion on outcomes in traumatic brain injury patients. Brain Inj.

[138] Miles MVP, Hicks RC, Parmer H, Brown C, Edwards A, Stewart K, Gao L, Maxwell R (2022). Traumatic brain injury patients with platelet inhibition receiving platelet transfusion demonstrate decreased need for neurosurgical intervention and decreased mortality. J Trauma Acute Care Surg.

[139] Furay E, Daley M, Teixeira PG, Coopwood TB, Aydelotte JD, Malesa N, Tellinghuisen C, Ali S, Brown LH, Brown CVR (2018). Goal-directed platelet transfusions correct platelet dysfunction and may improve survival in patients with severe traumatic brain injury. J Trauma Acute Care Surg.

[140] Pelaez CA, Spilman SK, Bell CT, Eastman DK, Sidwell RA (2019). Not all head injured patients on antiplatelet drugs need platelets: integrating platelet reactivity testing into platelet transfusion guidelines. Injury.

[141] Bickell WH, Wall MJ, Pepe PE, Martin RR, Ginger VF, Allen MK, Mattox KL (1994). Immediate versus delayed fluid resuscitation for hypotensive patients with penetrating torso injuries. N Engl J Med.

[142] Tran A, Yates J, Lau A, Lampron J, Matar M (2018). Permissive hypotension versus conventional resuscitation strategies in adult trauma patients with haemorrhagic shock: a systematic review and meta-analysis of randomised controlled trials. J Trauma Acute Care Surg.

[143] Safiejko K, Smereka J, Filipiak KJ, Szarpak A, Dabrowski M, Ladny JR, Jaguszewski MJ, Szarpak L. Effectiveness and safety of hypotension fluid resuscitation in traumatic haemorrhagic shock: a systematic review and meta-analysis of randomised controlled trials. Cardiol J. 2020. Preprint. https://www.ncbi.nlm.nih.gov/pubmed/32648249. 10.5603/CJ.a2020.0096 PMC9170316 32648249

[144] Wang C, Hsieh W, Chou H, Huang Y, Shen J, Yeo YH, Chang H, Chen S, Lee C (2014). Liberal versus restricted fluid resuscitation strategies in trauma patients: a systematic review and meta-analysis of randomised controlled trials and observational studies. Crit Care Med.

[145] Albreiki M, Voegeli D (2018). Permissive hypotensive resuscitation in adult patients with traumatic haemorrhagic shock: a systematic review. Eur J Trauma Emerg Surg.

[146] Owattanapanich N, Chittawatanarat K, Benyakorn T, Sirikun J (2018). Risks and benefits of hypotensive resuscitation in patients with traumatic haemorrhagic shock: a meta-analysis. Scand J Trauma Resusc Emerg Med.

[147] Joseph B, Azim A, Zangbar B, Bauman Z, O’Keeffe T, Ibraheem K, Kulvatunyou N, Tang A, Latifi R, Rhee P (2017). Improving mortality in trauma laparotomy through the evolution of damage control resuscitation: analysis of 1030 consecutive trauma laparotomies. J Trauma Acute Care Surg.

[148] Fischer NJ, Civil ID (2022). Haemorrhagic death from severe liver trauma has decreased in the era of haemostatic resuscitation. ANZ J Surg.

[149] Kasotakis G, Sideris A, Yang Y, de Moya M, Alam H, King DR, Tompkins R, Velmahos G (2013). Inflammation and host response to injury investigators: aggressive early crystalloid resuscitation adversely affects outcomes in adult blunt trauma patients: an analysis of the glue grant database. J Trauma Acute Care Surg.

[150] Mbadiwe N, Georgette N, Slidell MB, McQueen A (2021). Higher crystalloid volume during initial pediatric trauma resuscitation is associated with mortality. J Surg Res.

[151] Lou X, Lu G, Zhao M, Jin P (2018). Preoperative fluid management in traumatic shock: a retrospective study for identifying optimal therapy of fluid resuscitation for aged patients. Med (Baltimore).

[152] Barmparas G, Dhillon NK, Smith EJ, Mason R, Melo N, Thomsen GM, Margulies DR, Ley EJ (2018). Patterns of vasopressor utilization during the resuscitation of massively transfused trauma patients. Injury.

[153] Aoki M, Abe T, Saitoh D, Hagiwara S, Oshima K (2018). Use of vasopressor increases the risk of mortality in traumatic haemorrhagic shock: a nationwide cohort study in Japan. Crit Care Med.

[154] Uchida K, Nishimura T, Hagawa N, Kaga S, Noda T, Shinyama N, Yamamoto H, Mizobata Y (2020). The impact of early administration of vasopressor agents for the resuscitation of severe haemorrhagic shock following blunt trauma. BMC Emerg Med.

[155] Fisher AD, April MD, Cunningham C, Schauer SG (2021). Prehospital vasopressor use is associated with worse mortality in combat wounded. Prehosp Emerg Care.

[156] Gauss T, Gayat E, Harrois A, Raux M, Follin A, Daban JL, Cook F, Hamada S (2018). TraumaBase group, prehospital traumabase group Ile de France SAMU=service d’Aide Médicale Urgente: effect of early use of noradrenaline on in-hospital mortality in haemorrhagic shock after major trauma: a propensity-score analysis. Br J Anaesth.

[157] Van Haren RM, Thorson CM, Valle EJ, Guarch GA, Jouria JM, Busko AM, Namias N, Livingstone AS, Proctor KG (2014). Vasopressor use during emergency trauma surgery. Am Surg.

[158] Hylands M, Toma A, Beaudoin N, Frenette AJ, D’Aragon F, Belley-Côté É, Charbonney E, Møller MH, Laake JH, Vandvik PO (2017). Early vasopressor use following traumatic injury: a systematic review. BMJ Open.

[159] Richards JE, Harris T, Dünser MW, Bouzat P, Gauss T (2021). Vasopressors in trauma: A never event?. Anesth Analg.

[160] Sims CA, Holena D, Kim P, Pascual J, Smith B, Martin N, Seamon M, Shiroff A, Raza S, Kaplan L (2019). Effect of low-dose supplementation of arginine vasopressin on need for blood product transfusions in patients with trauma and haemorrhagic shock: a randomised clinical trial. JAMA Surg.

[161] Cohn SM, McCarthy J, Stewart RM, Jonas RB, Dent DL, Michalek JE (2011). Impact of low-dose vasopressin on trauma outcome: prospective randomised study. World J Surg.

[162] Semler MW, Kellum JA (2019). Balanced crystalloid solutions. Am J Respir Crit Care Med.

[163] Semler MW, Self WH, Wanderer JP, Ehrenfeld JM, Wang L, Byrne DW, Stollings JL, Kumar AB, Hughes CG, Hernandez A (2018). Balanced crystalloids versus saline in critically ill adults. N Engl J Med.

[164] Zampieri FG, Machado FR, Biondi RS, Freitas FGR, Veiga VC, Figueiredo RC, Lovato WJ, Amêndola CP, Serpa-Neto A, Paranhos JLR (2021). Effect of intravenous fluid treatment with a balanced solution vs 0.9% saline solution on mortality in critically ill patients: the BaSICS randomised clinical trial. JAMA.

[165] Antequera Martín AM, Barea Mendoza JA, Muriel A, Sáez I, Chico-Fernández M, Estrada-Lorenzo JM, Plana MN (2019). Buffered solutions versus 0.9% saline for resuscitation in critically ill adults and children. Cochrane Database Syst Rev.

[166] Zhu Y, Guo N, Song M, Xia F, Wu Y, Wang X, Chen T, Yang Z, Yang S, Zhang Y (2021). Balanced crystalloids versus saline in critically ill patients: the PRISMA study of a meta-analysis. Med (Baltimore).

[167] Rowell SE, Fair KA, Barbosa RR, Watters JM, Bulger EM, Holcomb JB, Cohen MJ, Rahbar MH, Fox EE, Schreiber MA (2016). The impact of pre-hospital administration of lactated Ringer's solution versus normal saline in patients with traumatic brain injury. J Neurotrauma.

[168] Roquilly A, Moyer JD, Huet O, Lasocki S, Cohen B, Dahyot-Fizelier C, Chalard K, Seguin P, Jeantrelle C, Vermeersch V (2021). Effect of continuous infusion of hypertonic saline vs standard care on 6-month neurological outcomes in patients with traumatic brain injury: the COBI randomised clinical trial. JAMA.

[169] de Crescenzo C, Gorouhi F, Salcedo ES, Galante JM (2017). Prehospital hypertonic fluid resuscitation for trauma patients: a systematic review and meta-analysis. J Trauma Acute Care Surg.

[170] Wu M, Liao T, Lee EM, Chen Y, Hsu W, Lee MG, Tsou P, Chen S, Lee C (2017). Administration of hypertonic solutions for haemorrhagic shock: a systematic review and meta-analysis of clinical trials. Anesth Analg.

[171] Orbegozo Cortés D, Gamarano Barros T, Njimi H, Vincent J (2015). Crystalloids versus colloids: exploring differences in fluid requirements by systematic review and meta-regression. Anesth Analg.

[172] Tseng C, Chen T, Wu M, Chan M, Shih M, Tu Y (2020). Resuscitation fluid types in sepsis, surgical, and trauma patients: a systematic review and sequential network meta-analyses. Crit Care.

[173] Lewis SR, Pritchard MW, Evans DJ, Butler AR, Alderson P, Smith AF, Roberts I (2018). Colloids versus crystalloids for fluid resuscitation in critically ill people. Cochrane Database Syst Rev.

[174] Chappell D, van der Linden P, Ripollés-Melchor J, James MFM (2021). Safety and efficacy of tetrastarches in surgery and trauma: a systematic review and meta-analysis of randomised controlled trials. Br J Anaesth.

[175] Kind SL, Spahn-Nett GH, Emmert MY, Eismon J, Seifert B, Spahn DR, Theusinger OM (2013). Is dilutional coagulopathy induced by different colloids reversible by replacement of fibrinogen and factor XIII concentrates?. Anesth Analg.

[176] Groene P, Wiederkehr T, Kammerer T, Möhnle P, Maerte M, Bayer A, Görlinger K, Rehm M, Schäfer ST (2020). Comparison of two different fibrinogen concentrates in an in vitro model of dilutional coagulopathy. Transfus Med Hemother.

[177] Carson JL, Stanworth SJ, Dennis JA, Trivella M, Roubinian N, Fergusson DA, Triulzi D, Dorée C, Hébert PC (2021). Transfusion thresholds for guiding red blood cell transfusion. Cochrane Database Syst Rev.

[178] Garland-Kledzik M, Gaffley M, Crouse D, Conrad C, Miller P, Martin RS (2019). Effects of a more restrictive transfusion trigger in trauma patients. Am Surg.

[179] Florez-Perdomo WA, García-Ballestas E, Martinez-Perez R, Agrawal A, Deora H, Joaquim AF, Quiñones-Ossa GA, Moscote-Salazar LR. Haemoglobin levels as a transfusion criterion in moderate to severe traumatic brain injury: a systematic review and meta-analysis. Br J Neurosurg 2021:1–7. 10.1080/02688697.2021.1940850. 10.1080/02688697.2021.1940850 34148446

[180] Ngwenya LB, Suen CG, Tarapore PE, Manley GT, Huang MC (2018). Safety and cost efficiency of a restrictive transfusion protocol in patients with traumatic brain injury. J Neurosurg.

[181] Robertson CS, Hannay HJ, Yamal JM, Gopinath S, Goodman JC, Tilley BC, Baldwin A, Rivera Lara L, Saucedo-Crespo H, Ahmed O (2014). Effect of erythropoietin and transfusion threshold on neurological recovery after traumatic brain injury: a randomised clinical trial. JAMA.

[182] Vedantam A, Yamal JM, Rubin ML, Robertson CS, Gopinath SP (2016). Progressive haemorrhagic injury after severe traumatic brain injury: effect of haemoglobin transfusion thresholds. J Neurosurg.

[183] Gobatto ALN, Link MA, Solla DJ, Bassi E, Tierno PF, Paiva W, Taccone FS, Malbouisson LM (2019). Transfusion requirements after head trauma: a randomised feasibility controlled trial. Crit Care.

[184] Li J, Sun SL, Tian JH, Yang K, Liu R, Li J (2015). Cell salvage in emergency trauma surgery. Cochrane Database Syst Rev.

[185] Boyle G, Kuffel A, Parmar K, Gibson K, Smith M, Grehan A, Hunt BJ, Chambers DJ (2019). A comparison of haemostatic biomarkers during low-risk patients undergoing cardiopulmonary bypass using either conventional centrifugal cell salvage or the HemoSep device. Perfusion.

[186] Roets M, Sturgess DJ, Obeysekera MP, Tran TV, Wyssusek KH, Punnasseril JEJ, da Silva D, van Zundert A, Perros AJ, Tung JP (2020). Intraoperative cell salvage as an alternative to allogeneic (donated) blood transfusion: a prospective observational evaluation of the immune response profile. Cell Transplant.

[187] Bowley DM, Barker P, Boffard KD (2006). Intraoperative blood salvage in penetrating abdominal trauma: a randomised, controlled trial. World J Surg.

[188] Brown CVR, Foulkrod KH, Sadler HT, Richards EK, Biggan DP, Czysz C, Manuel T (2010). Autologous blood transfusion during emergency trauma operations. Arch Surg.

[189] Bhangu A, Nepogodiev D, Doughty H, Bowley DM (2013). Intraoperative cell salvage in a combat support hospital: a prospective proof of concept study. Transfusion.

[190] Caliste XA, McArthur KA, Sava JA (2014). Autotransfusion in emergent operative trauma resuscitation. Eur J Trauma Emerg Surg.

[191] Reitano E, Granieri S, Frassini S, Sammartano F, Cimbanassi S, Chiara O (2021). Infectious complications of extra-peritoneal pelvic packing in emergency room. Updates Surg.

[192] Firoozabadi R, Swenson A, Kleweno C, Routt MC (2015). Cell saver use in acetabular surgery: Does approach matter?. J Orthop Trauma.

[193] Odak S, Raza A, Shah N, Clayson A (2013). Clinical efficacy and cost effectiveness of intraoperative cell salvage in pelvic trauma surgery. Ann R Coll Surg Engl.

[194] Jawed A, Ahmed A, Williams MR (2019). Intra-operative cell salvage in pelvic and acetabular fracture surgery: a retrospective comparative study. Int Orthop.

[195] Rhee P, Inaba K, Pandit V, Khalil M, Siboni S, Vercruysse G, Kulvatunyou N, Tang A, Asif A, O'Keeffe T (2015). Early autologous fresh whole blood transfusion leads to less allogeneic transfusions and is safe. J Trauma Acute Care Surg.

[196] Sahloul M, Bowley D, Kirkman E, Doughty H (2018). Blood salvage technology after combat injury. J R Army Med Corps.

[197] Jurkovich GJ, Greiser WB, Luterman A, Curreri PW (1987). Hypothermia in trauma victims: an ominous predictor of survival. J Trauma.

[198] Lester ELW, Fox EE, Holcomb JB, Brasel KJ, Bulger EM, Cohen MJ, Cotton BA, Fabian TC, Kerby JD, O’Keefe T (2019). The impact of hypothermia on outcomes in massively transfused patients. J Trauma Acute Care Surg.

[199] Duque P, Calvo A, Lockie C, Schochl H (2021). Pathophysiology of trauma-induced coagulopathy. Transfus Med Rev.

[200] Reynolds BR, Forsythe RM, Harbrecht BG, Cuschieri J, Minei JP, Maier RV, Moore EE, Billiar EE, Peitzman AB, Sperry JL (2012). Hypothermia in massive transfusion: Have we been paying enough attention to it?. J Trauma Acute Care Surg.

[201] Rubiano AM, Sanchez AI, Estebanez G, Peitzman A, Sperry J, Puyana JC (2013). The effect of admission spontaneous hypothermia on patients with severe traumatic brain injury. Injury.

[202] Cooper DJ, Nichol AD, Bailey M, Bernard S, Cameron PA, Pili-Floury S, Forbes A, Gantner D, Higgins AM, Huet O (2018). Effect of early sustained prophylactic hypothermia on neurologic outcomes among patients with severe traumatic brain injury: the POLAR randomised clinical trial. JAMA.

[203] Hui J, Feng J, Tu Y, Zhang W, Zhong C, Liu M, Wang Y, Long L, Chen L, Liu J (2021). Safety and efficacy of long-term mild hypothermia for severe traumatic brain injury with refractory intracranial hypertension (LTH-1): a multicentre randomised controlled trial. EClinicalMedicine.

[204] Pegoli M, Zurlo Z, Bilotta F (2020). Temperature management in acute brain injury: a systematic review of clinical evidence. Clin Neurol Neurosurg.

[205] Andrews PJ, Sinclair HL, Rodríguez A, Harris B, Rhodes J, Watson H, Murray G (2018). Therapeutic hypothermia to reduce intracranial pressure after traumatic brain injury: the Eurotherm3235 RCT. Health Technol Assess.

[206] Chen H, Wu F, Yang P, Shao J, Chen Q, Zheng R (2019). A meta-analysis of the effects of therapeutic hypothermia in adult patients with traumatic brain injury. Crit Care.

[207] Bennett BL, Giesbrect G, Zafren K, Christensen R, Littlejohn LF, Drew B, Cap AP, Miles EA, Butler FK, Holcomb JB (2020). Management of hypothermia in tactical combat casualty care: TCCC guideline proposed change 20–01 (June 2020). J Spec Oper Med.

[208] Stone HH, Strom PR, Mullins RJ (1983). Management of the major coagulopathy with onset during laparotomy. Ann Surg.

[209] Rotondo MF, Schwab CW, McGonigal MD, Phillips GR, Fruchterman TM, Kauder DR, Latenser BA, Angood PA (1993). Damage control: an approach for improved survival in exsanguinating penetrating abdominal injury. J Trauma.

[210] Roberts DJ, Ball CG, Feliciano DV, Moore EE, Ivatury RR, Lucas CE, Fabian TC, Zygun DA, Kirkpatrick AW, Stelfox HT (2017). History of the innovation of damage control for management of trauma patients: 1902–2016. Ann Surg.

[211] Duchesne JC, McSwain NE, Cotton BA, Hunt JP, Dellavolpe J, Lafaro K, Marr AB, Gonzalez EA, Phelan HA, Bilski T (2010). Damage control resuscitation: the new face of damage control. J Trauma.

[212] Roberts DJ, Bobrovitz N, Zygun DA, Kirkpatrick AW, Ball CG, Faris PD, Stelfox HT (2021). Indications for trauma damage control surgery international study group: evidence for use of damage control surgery and damage control interventions in civilian trauma patients: a systematic review. World J Emerg Surg.

[213] Cullinane DC, Schiller HJ, Zielinski MD, Bilaniuk JW, Collier BR, Como J, Holevar M, Sabater EA, Sems SA, Vassy WM (2011). Eastern Association for the Surgery of Trauma practice management guidelines for haemorrhage in pelvic fracture-update and systematic review. J Trauma.

[214] Höch A, Zeidler S, Pieroh P, Josten C, Stuby FM, Herath SC (2021). German pelvic trauma registry: trends and efficacy of external emergency stabilisation of pelvic ring fractures: results from the German Pelvic Trauma Registry. Eur J Trauma Emerg Surg.

[215] Naseem H, Nesbitt PD, Sprott DC, Clayson A (2018). An assessment of pelvic binder placement at a UK major trauma centre. Ann R Coll Surg Engl.

[216] Audretsch CK, Mader D, Bahrs C, Trulson A, Höch A, Herath SC, Küper MA (2021). Working group on pelvic fractures of the German Trauma Society: comparison of pelvic C-clamp and pelvic binder for emergency stabilisation and bleeding control in type-C pelvic ring fractures. Sci Rep.

[217] Kim MJ, Lee JG, Kim EH, Lee SH (2021). A nomogram to predict arterial bleeding in patients with pelvic fractures after blunt trauma: a retrospective cohort study. J Orthop Surg Res.

[218] Magnone S, Allievi N, Ceresoli M, Coccolini F, Pisano M, Ansaloni L (2021). Prospective validation of a new protocol with preperitoneal pelvic packing as the mainstay for the treatment of haemodynamically unstable pelvic trauma: a 5-year experience. Eur J Trauma Emerg Surg.

[219] Duchesne J, Costantini TW, Khan M, Taub E, Rhee P, Morse B, Namias N, Schwarz A, Graves J, Kim DY (2019). The effect of haemorrhage control adjuncts on outcome in severe pelvic fracture: a multi-institutional study. J Trauma Acute Care Surg.

[220] Shim H, Jang JY, Kim JW, Ryu H, Jung PY, Kim S, Kwon HY, Kim KM, Chung H, Bae KS (2018). Effectiveness and postoperative wound infection of preperitoneal pelvic packing in patients with haemodynamic instability caused by pelvic fracture. PLoS ONE.

[221] Alnumay A, Caminsky N, Eustache JH, Valenti D, Beckett AN, Deckelbaum D, Fata P, Khwaja K, Razek T, McKendy KM (2022). Feasibility of intraoperative angioembolisation for trauma patients using C-arm digital subtraction angiography. Eur J Trauma Emerg Surg.

[222] ClinicalTrials.gov. U.S. National Library of Medicine: Pelvic fractures in polytraumatized patients with haemodynamic instability: angioembolisation vs preperitoneal packing. 2021. https://clinicaltrials.gov/ct2/show/NCT04764864. Accessed 11 Jul 2022.

[223] Muntasar AE, Toner E, Alkhazaaleh OA, Arumugam D, Shah N, Hajibandeh S, Hajibandeh S (2018). Effect of angioembolisation versus surgical packing on mortality in traumatic pelvic haemorrhage: a systematic review and meta-analysis. World J Emerg Med.

[224] Asmar S, Bible L, Chehab M, Tang A, Khurrum M, Douglas M, Castanon L, Kulvatunyou N, Joseph B (2021). Resuscitative endovascular balloon occlusion of the aorta vs pre-peritoneal packing in patients with pelvic fracture. J Am Coll Surg.

[225] Frassini S, Gupta S, Granieri S, Cimbanassi S, Sammartano F, Scalea TM, Chiara O (2021). Emergency management of pelvic bleeding. J Clin Med.

[226] Moore LJ, Rasmussen TE (2022). A contemporary assessment of resuscitative endovascular balloon occlusion of the aorta. J Trauma Acute Care Surg.

[227] Kinslow K, Shepherd A, McKenney M, Elkbuli A (2022). Resuscitative endovascular balloon occlusion of aorta: a systematic review. Am Surg.

[228] Castellini G, Gianola S, Biffi A, Porcu G, Fabbri A, Ruggieri MP, Coniglio C, Napoletano A, Coclite D, D'Angelo D (2021). Resuscitative endovascular balloon occlusion of the aorta (REBOA) in patients with major trauma and uncontrolled haemorrhagic shock: a systematic review with meta-analysis. World J Emerg Surg.

[229] Harfouche MN, Madurska MJ, Elansary N, Abdou H, Lang E, DuBose JJ, Kundi R, Feliciano DV, Scalea TM, Morrison JJ (2022). Resuscitative endovascular balloon occlusion of the aorta associated with improved survival in haemorrhagic shock. PLoS ONE.

[230] Cantle PM (2022). REBOA utility. Surg Open Sci.

[231] Pursifull NF, Morris MS, Harris RA, Morey AF (2006). Damage control management of experimental grade 5 renal injuries: further evaluation of FloSeal gelatin matrix. J Trauma.

[232] Schenk WG, Burks SG, Gagne PJ, Kagan SA, Lawson JH, Spotnitz WD (2003). Fibrin sealant improves haemostasis in peripheral vascular surgery: a randomised prospective trial. Ann Surg.

[233] Sherman R, Chapman WC, Hannon G, Block JE (2001). Control of bone bleeding at the sternum and iliac crest donor sites using a collagen-based composite combined with autologous plasma: results of a randomised controlled trial. Orthopedics.

[234] Testini M, Marzaioli R, Lissidini G, Lippolis A, Logoluso F, Gurrado A, Lardo D, Poli E, Piccinni G (2009). The effectiveness of FloSeal matrix haemostatic agent in thyroid surgery: a prospective, randomised, control study. Langenbecks Arch Surg.

[235] Weaver FA, Hood DB, Zatina M, Messina L, Badduke B (2002). Gelatin-thrombin-based haemostatic sealant for intraoperative bleeding in vascular surgery. Ann Vasc Surg.

[236] Witte B, Kroeber SM, Hillebrand H, Wolf M, Huertgen M (2013). Cotton-derived oxidised cellulose in minimally invasive thoracic surgery: a clinicopathological study. Innovations (Phila).

[237] Woodworth BA, Chandra RK, LeBenger JD, Ilie B, Schlosser RJ (2009). A gelatin-thrombin matrix for haemostasis after endoscopic sinus surgery. Am J Otolaryngol.

[238] Winstanley M, Smith JE, Wright C (2019). Catastrophic haemorrhage in military major trauma patients: a retrospective database analysis of haemostatic agents used on the battlefield. J R Army Med Corps.

[239] Welch M, Barratt J, Peters A, Wright C (2020). Systematic review of prehospital haemostatic dressings. BMJ Mil Health.

[240] Choron RL, Hazelton JP, Hunter K, Capano-Wehrle L, Gaughan J, Chovanes J, Seamon MJ (2017). Intra-abdominal packing with laparotomy pads and QuikClot™ during damage control laparotomy: a safety analysis. Injury.

[241] Shakur H, Roberts I, Bautista R, Caballero J, Coats T, Dewan Y, El-Sayed H, Gogichaishvili T, Gupta S, CRASH-2 trial collaborators (2010). Effects of tranexamic acid on death, vascular occlusive events, and blood transfusion in trauma patients with significant haemorrhage (CRASH-2): a randomised, placebo-controlled trial. Lancet.

[242] CRASH-3 trial collaborators (2019). Effects of tranexamic acid on death, disability, vascular occlusive events and other morbidities in patients with acute traumatic brain injury (CRASH-3): a randomised, placebo-controlled trial. Lancet.

[243] Rowell SE, Meier EN, McKnight B, Kannas D, May S, Sheehan K, Bulger EM, Idris AH, Christenson J, Morrison LJ (2020). Effect of out-of-hospital tranexamic acid vs placebo on 6-month functional neurologic outcomes in patients with moderate or severe traumatic brain injury. JAMA.

[244] Mojallal F, Nikooieh M, Hajimaghsoudi M, Baqherabadi M, Jafari M, Esmaeili A, Karimi NM, Zarepur E (2020). The effect of intravenous tranexamic acid on preventing the progress of cerebral haemorrhage in patients with brain traumatic injuries compared to placebo: a randomised clinical trial. Med J Islam Repub Iran.

[245] Fakharian E, Abedzadeh-Kalahroudi M, Atoof F (2018). Effect of tranexamic acid on prevention of haemorrhagic mass growth in patients with traumatic brain injury. World Neurosurg.

[246] Roberts I, Shakur-Still H, Aeron-Thomas A, Beaumont D, Belli A, Brenner A, Cargill M, Chaudhri R, Douglas N, Frimley L (2021). Tranexamic acid to reduce head injury death in people with traumatic brain injury: the CRASH-3 international RCT. Health Technol Assess.

[247] CRASH-3 Intracranial Bleeding Mechanistic Study collaborators (2021). Tranexamic acid in traumatic brain injury: an explanatory study nested within the CRASH-3 trial. Eur J Trauma Emerg Surg.

[248] Li SR, Guyette F, Brown J, Zenati M, Reitz KM, Eastridge B, Nirula R, Vercruysse GA, O'Keeffe T, Joseph B (2021). Early prehospital tranexamic acid following injury is associated with a 30-day survival benefit: a secondary analysis of a randomised clinical trial. Ann Surg.

[249] Dixon AL, McCully BH, Rick EA, Dewey E, Farrell DH, Morrison LJ, McMullan J, Robinson BRH, Callum J, Tibbs B (2020). Tranexamic acid administration in the field does not affect admission thromboelastography after traumatic brain injury. J Trauma Acute Care Surg.

[250] Perkins ZB, Yet B, Marsden M, Glasgow S, Marsh W, Davenport R, Brohi K, Tai NRM (2021). Early identification of trauma-induced coagulopathy: development and validation of a multivariable risk prediction model. Ann Surg.

[251] Innerhofer P, Fries D, Mittermayr M, Innerhofer N, von Langen D, Hell T, Gruber G, Schmid S, Friesenecker B, Lorenz IH (2017). Reversal of trauma-induced coagulopathy using first-line coagulation factor concentrates or fresh frozen plasma (RETIC): a single-centre, parallel-group, open-label, randomised trial. Lancet Haematol.

[252] Schöchl H, Nienaber U, Maegele M, Hochleitner G, Primavesi F, Steitz B, Arndt C, Hanke A, Voelckel W, Solomon C (2011). Transfusion in trauma: thromboelastometry-guided coagulation factor concentrate-based therapy versus standard fresh frozen plasma-based therapy. Crit Care.

[253] Gonzalez E, Moore EE, Moore HB, Chapman MP, Chin TL, Ghasabyan A, Wohlauer MV, Barnett CC, Bensard DD, Biffl WL (2016). Goal-directed haemostatic resuscitation of trauma-induced coagulopathy: a pragmatic randomised clinical trial comparing a viscoelastic assay to conventional coagulation assays. Ann Surg.

[254] Cochrane C, Chinna S, Um JY, Dias JD, Hartmann J, Bradley J, Brooks A (2020). Site-of-care viscoelastic assay in major trauma improves outcomes and is cost neutral compared with standard coagulation tests. Diagnostics (Basel).

[255] Lammers DT, Marenco CW, Morte KR, Bingham JR, Martin MJ, Eckert MJ (2020). Viscoelastic testing in combat resuscitation: Is it time for a new standard?. J Trauma Acute Care Surg.

[256] Baksaas-Aasen K, Gall L, Eaglestone S, Rourke C, Juffermans NP, Goslings JC, Naess PA, van Dieren S, Ostrowski SR, Stensballe J (2017). iTACTIC-implementing treatment algorithms for the correction of trauma-induced coagulopathy: study protocol for a multicentre, randomised controlled trial. Trials.

[257] Holcomb JB, Tilley BC, Baraniuk S, Fox EE, Wade CE, Podbielski JM, del Junco DJ, Brasel KJ, Bulger EM, Callcut RA (2015). Transfusion of plasma, platelets, and red blood cells in a 1:1:1 vs a 1:1:2 ratio and mortality in patients with severe trauma: the PROPPR randomised clinical trial. JAMA.

[258] Meneses E, Boneva D, McKenney M, Elkbuli A (2020). Massive transfusion protocol in adult trauma population. Am J Emerg Med.

[259] Cardenas JC, Zhang X, Fox EE, Cotton BA, Hess JR, Schreiber MA, Wade CE, Holcomb JB (2018). Platelet transfusions improve haemostasis and survival in a substudy of the prospective, randomised PROPPR trial. Blood Adv.

[260] Nguyen M, Pirracchio R, Kornblith LZ, Callcut R, Fox EE, Wade CE, Schreiber M, Holcomb JB, Coyle J, Cohen M (2020). Dynamic impact of transfusion ratios on outcomes in severely injured patients: targeted machine learning analysis of the pragmatic, randomised optimal platelet and plasma ratios randomised clinical trial. J Trauma Acute Care Surg.

[261] Peralta R, Vijay A, El-Menyar A, Consunji R, Afifi I, Mahmood I, Asim M, Latifi R, Al-Thani H (2016). Early high ratio platelet transfusion in trauma resuscitation and its outcomes. Int J Crit Illn Inj Sci.

[262] Hamada SR, Garrigue D, Nougue H, Meyer A, Boutonnet M, Meaudre E, Culver A, Gaertner E, Audibert G, Vigué B (2022). Impact of platelet transfusion on outcomes in trauma patients. Crit Care.

[263] Černý V, Maegele M, Agostini V, Fries D, Leal-Noval SR, Nardai G, Nardi G, Östlund A, Schöchl H (2022). Variations and obstacles in the use of coagulation factor concentrates for major trauma bleeding across Europe: outcomes from a European expert meeting. Eur J Trauma Emerg Surg.

[264] Collins PW, Solomon C, Sutor K, Crispin D, Hochleitner G, Rizoli S, Schöchl H, Schreiber M, Ranucci M (2014). Theoretical modelling of fibrinogen supplementation with therapeutic plasma, cryoprecipitate, or fibrinogen concentrate. Br J Anaesth.

[265] Nardi G, Agostini V, Rondinelli B, Russo E, Bastianini B, Bini G, Bulgarelli S, Cingolani E, Donato A, Gambale G (2015). Trauma-induced coagulopathy: impact of the early coagulation support protocol on blood product consumption, mortality and costs. Crit Care.

[266] Winearls J, Wullschleger M, Wake E, Hurn C, Furyk J, Ryan G, Trout M, Walsham J, Holley A, Cohen J (2017). Fibrinogen early in severe trauma study (FEISTY): study protocol for a randomised controlled trial. Trials.

[267] Hamada SR, Pirracchio R, Beauchesne J, Benlaldj MN, Meaudre E, Leone M, Pottecher J, Abback PS, Gauss T, Boutonnet M (2020). Effect of fibrinogen concentrate administration on early mortality in traumatic haemorrhagic shock: a propensity score analysis. J Trauma Acute Care Surg.

[268] Schöchl H, Maegele M, Voelckel W (2016). Fixed ratio versus goal-directed therapy in trauma. Curr Opin Anaesthesiol.

[269] Bainbridge FJ, Sinha R, Tocchetti R, Clarke C, Martin D, Foo N, Palmer CS, Ellis DY (2021). Introduction of point-of-care ROTEM testing in the emergency department of an Australian level 1 trauma centre and its effect on blood product use. Emerg Med Australas.

[270] Nascimento B, Callum J, Tien H, Rubenfeld G, Pinto R, Lin Y, Rizoli S (2013). Effect of a fixed-ratio (1:1:1) transfusion protocol versus laboratory-results-guided transfusion in patients with severe trauma: a randomised feasibility trial. CMAJ.

[271] Hilbert-Carius P, Hofmann G, Stuttmann R (2015). Haemoglobin-oriented and coagulation factor-based algorithm : effect on transfusion needs and standardized mortality rate in massively transfused trauma patients. Anaesthesist.

[272] Gratz J, Güting H, Thorn S, Brazinova A, Görlinger K, Schäfer N, Schöchl H, Stanworth S, Maegele M (2019). Protocolised thromboelastometric-guided haemostatic management in patients with traumatic brain injury: a pilot study. Anaesthesia.

[273] Inaba K, Rizoli S, Veigas PV, Callum J, Davenport R, Hess J, Maegele M (2015). Viscoelastic testing in trauma consensus panel: 2014 consensus conference on viscoelastic test-based transfusion guidelines for early trauma resuscitation: report of the panel. J Trauma Acute Care Surg.

[274] Einersen PM, Moore EE, Chapman MP, Moore HB, Gonzalez E, Silliman CC, Banerjee A, Sauaia A (2017). Rapid thrombelastography thresholds for goal-directed resuscitation of patients at risk for massive transfusion. J Trauma Acute Care Surg.

[275] Campbell D, Wake E, Walters K, Ho D, Keijzers G, Wullschleger M, Winearls J (2020). Implementation of point-of-care ROTEM(R) into a trauma major haemorrhage protocol: a before and after study. Emerg Med Australas.

[276] Ponschab M, Voelckel W, Pavelka M, Schlimp CJ, Schochl H (2015). Effect of coagulation factor concentrate administration on ROTEM(R) parameters in major trauma. Scand J Trauma Resusc Emerg Med.

[277] Rimaitis M, Bilskiene D, Tamosuitis T, Vilcinis R, Rimaitis K, Macas A (2020). Implementation of thromboelastometry for coagulation management in isolated traumatic brain injury patients undergoing craniotomy. Med Sci Monit.

[278] Whiting P, Al M, Westwood M, Ramos IC, Ryder S, Armstrong N, Misso K, Ross J, Severens J, Kleijnen J (2015). Viscoelastic point-of-care testing to assist with the diagnosis, management and monitoring of haemostasis: a systematic review and cost-effectiveness analysis. Health Technol Assess.

[279] Fahrendorff M, Oliveri RS, Johansson PI (2017). The use of viscoelastic haemostatic assays in goal-directing treatment with allogeneic blood products - a systematic review and meta-analysis. Scand J Trauma Resusc Emerg Med.

[280] Bugaev N, Como JJ, Golani G, Freeman JJ, Sawhney JS, Vatsaas CJ, Yorkgitis BK, Kreiner LA, Garcia NM, Aziz HA (2020). Thromboelastography and rotational thromboelastometry in bleeding patients with coagulopathy: practice management guideline from the Eastern Association for the Surgery of Trauma. J Trauma Acute Care Surg.

[281] Santos AS, Oliveira AJF, Barbosa MCL, Nogueira JLDS (2020). Viscoelastic haemostatic assays in the perioperative period of surgical procedures: systematic review and meta-analysis. J Clin Anesth.

[282] Wikkelsø A, Wetterslev J, Møller AM, Afshari A (2016). Thromboelastography (TEG) or thromboelastometry (ROTEM) to monitor haemostatic treatment versus usual care in adults or children with bleeding. Cochrane Database Syst Rev.

[283] Barelli S, Alberio L (2018). The role of plasma transfusion in massive bleeding: Protecting the endothelial glycocalyx?. Front Med (Lausanne).

[284] Adam EH, Fischer D (2020). Plasma transfusion practice in adult surgical patients: systematic review of the literature. Transfus Med Hemother.

[285] Zhang L, Li R, Zhao X, Zhang Q, Luo X (2017). Increased transfusion of fresh frozen plasma is associated with mortality or worse functional outcomes after severe traumatic brain injury: a retrospective study. World Neurosurg.

[286] Nederpelt CJ, El Hechi MW, Kongkaewpaisan N, Kokoroskos N, Mendoza AE, Saillant NN, Fagenholz PJ, King DR, Velmahos GC, Kaafarani HM (2020). Fresh frozen plasma-to-packed red blood cell ratio and mortality in traumatic haemorrhage: nationwide analysis of 4427 patients. J Am Coll Surg.

[287] Chang R, Kerby JD, Kalkwarf KJ, Van Belle G, Fox EE, Cotton BA, Cohen MJ, Schreiber MA, Brasel K, Bulger EM (2019). Earlier time to haemostasis is associated with decreased mortality and rate of complications: results from the pragmatic randomised optimal platelet and plasma ratio trial. J Trauma Acute Care Surg.

[288] Khan S, Davenport R, Raza I, Glasgow S, De'Ath HD, Johansson PI, Curry N, Stanworth S, Gaarder C, Brohi K (2015). Damage control resuscitation using blood component therapy in standard doses has a limited effect on coagulopathy during trauma haemorrhage. Intensive Care Med.

[289] Gratz J, Ponschab M, Iapichino GE, Schlimp CJ, Cadamuro J, Grottke O, Zipperle J, Oberladstätter D, Gabriel C, Ziegler B (2020). Comparison of fresh frozen plasma vs. coagulation factor concentrates for reconstitution of blood: an in vitro study. Eur J Anaesthesiol.

[290] Akbari E, Safari S, Hatamabadi H (2018). The effect of fibrinogen concentrate and fresh frozen plasma on the outcome of patients with acute traumatic coagulopathy: a quasi-experimental study. Am J Emerg Med.

[291] Allen CJ, Shariatmadar S, Meizoso JP, Hanna MM, Mora JL, Ray JJ, Namias N, Dudaryk R, Proctor KG (2015). Liquid plasma use during "super" massive transfusion protocol. J Surg Res.

[292] Fadeyi EA, Saha AK, Soltani S, Naal T, Palmer R, Bakht A, Warren CS, Simmons JH, Pomper GJ (2022). A comparison between liquid group A plasma and thawed group A plasma for massive transfusion activation in trauma patients. Vox Sang.

[293] Mok G, Hoang R, Khan MW, Pannell D, Peng H, Tien H, Nathens A, Callum J, Karkouti K, Beckett A (2021). Freeze-dried plasma for major trauma-systematic review and meta-analysis. J Trauma Acute Care Surg.

[294] de Roulet A, Kerby JD, Weinberg JA, Lewis RH, Hudgins JP, Shulman IA, Fox EE, Holcomb JB, Brasel KJ, Bulger EM (2020). Group A emergency-release plasma in trauma patients requiring massive transfusion. J Trauma Acute Care Surg.

[295] Beynon C, Nofal M, Rizos T, Laible M, Sakowitz OW, Unterberg AW (2020). Prothrombin complex concentrate for vitamin K antagonist reversal in traumatic intracranial haemorrhage. J Clin Neurosci.

[296] Tanaka KA, Shettar S, Vandyck K, Shea SM, Abuelkasem E (2021). Roles of four-factor prothrombin complex concentrate in the management of critical bleeding. Transfus Med Rev.

[297] Kao T, Lee Y, Chang H (2021). Prothrombin complex concentrate for trauma induced coagulopathy: a systematic review and meta-analysis. J Acute Med.

[298] Dunbar NM, Chandler WL (2009). Thrombin generation in trauma patients. Transfusion.

[299] Schöchl H, Nienaber U, Hofer G, Voelckel W, Jambor C, Scharbert G, Kozek-Langenecker S, Solomon C (2010). Goal-directed coagulation management of major trauma patients using thromboelastometry (ROTEM)-guided administration of fibrinogen concentrate and prothrombin complex concentrate. Crit Care.

[300] Schöchl H, Voelckel W, Maegele M, Kirchmair L, Schlimp CJ (2014). Endogenous thrombin potential following haemostatic therapy with 4-factor prothrombin complex concentrate: a 7-day observational study of trauma patients. Crit Care.

[301] Hethershaw EL, Cilia La Corte AL, Duval C, Ali M, Grant PJ, Ariëns RA, Philippou H (2014). The effect of blood coagulation factor XIII on fibrin clot structure and fibrinolysis. J Thromb Haemost.

[302] Kozek-Langenecker SA, Ahmed AB, Afshari A, Albaladejo P, Aldecoa C, Barauskas G, De Robertis E, Faraoni D, Filipescu DC, Fries D (2017). Management of severe perioperative bleeding: guidelines from the European Society of Anaesthesiology: first update 2016. Eur J Anaesthesiol.

[303] Curry N, Rourke C, Davenport R, Beer S, Pankhurst L, Deary A, Thomas H, Llewelyn C, Green L, Doughty H (2015). Early cryoprecipitate for major haemorrhage in trauma: a randomised controlled feasibility trial. Br J Anaesth.

[304] Curry N, Foley C, Wong H, Mora A, Curnow E, Zarankaite A, Hodge R, Hopkins V, Deary A, Ray J (2018). Early fibrinogen concentrate therapy for major haemorrhage in trauma (E-FIT 1): results from a UK multi-centre, randomised, double blind, placebo-controlled pilot trial. Crit Care.

[305] Lucena LS, Rodrigues RDR, Carmona MJC, Noronha FJD, Oliveira HP, Lima NM, Pinheiro RB, Silva WAD, Cavalcanti AB (2021). Early administration of fibrinogen concentrate in patients with polytrauma with thromboelastometry suggestive of hypofibrinogenemia: a randomised feasibility trial. Clinics (Sao Paulo).

[306] Nascimento B, Callum J, Tien H, Peng H, Rizoli S, Karanicolas P, Alam A, Xiong W, Selby R, Garzon AM (2016). Fibrinogen in the initial resuscitation of severe trauma (FiiRST): a randomised feasibility trial. Br J Anaesth.

[307] Ziegler B, Bachler M, Haberfellner H, Niederwanger C, Innerhofer P, Hell T, Kaufmann M, Maegele M, Martinowitz U, Nebl C (2021). Efficacy of prehospital administration of fibrinogen concentrate in trauma patients bleeding or presumed to bleed (FIinTIC): a multicentre, double-blind, placebo-controlled, randomised pilot study. Eur J Anaesthesiol.

[308] Almskog LM, Hammar U, Wikman A, Östlund A, Svensson J, Wanecek M, Ågren A (2020). A retrospective register study comparing fibrinogen treated trauma patients with an injury severity score matched control group. Scand J Trauma Resusc Emerg Med.

[309] Stabler SN, Li SS, Karpov A, Vu EN (2020). Use of fibrinogen concentrate for trauma-related bleeding: a systematic-review and meta-analysis. J Trauma Acute Care Surg.

[310] Tauber H, Innerhofer N, von Langen D, Ströhle M, Fries D, Mittermayr M, Hell T, Oswald E, Innerhofer P (2020). Dynamics of platelet counts in major trauma: the impact of haemostatic resuscitation and effects of platelet transfusion-a sub-study of the randomised controlled RETIC trial. J Clin Med.

[311] Estcourt LJ, Birchall J, Allard S, Bassey SJ, Hersey P, Kerr JP, Mumford AD, Stanworth SJ, Tinegate H (2017). British committee for standards in haematology: guidelines for the use of platelet transfusions. Br J Haematol.

[312] Dorken Gallastegi A, Naar L, Gaitanidis A, Gebran A, Nederpelt CJ, Parks JJ, Hwabejire JO, Fawley J, Mendoza AE, Saillant NN (2022). Do not forget the platelets: the independent impact of red blood cell to platelet ratio on mortality in massively transfused trauma patients. J Trauma Acute Care Surg.

[313] Kleinveld DJB, van Amstel RBE, Wirtz MR, Geeraedts LMG, Goslings JC, Hollmann MW, Juffermans NP (2021). Platelet-to-red blood cell ratio and mortality in bleeding trauma patients: a systematic review and meta-analysis. Transfusion.

[314] Rijnhout TWH, Duijst J, Noorman F, Zoodsma M, van Waes OJF, Verhofstad MHJ, Hoencamp R (2021). Platelet to erythrocyte transfusion ratio and mortality in massively transfused trauma patients. A systematic review and meta-analysis. J Trauma Acute Care Surg.

[315] Kornblith LZ, Decker A, Conroy AS, Hendrickson CM, Fields AT, Robles AJ, Callcut RA, Cohen MJ (2019). It's about time: transfusion effects on postinjury platelet aggregation over time. J Trauma Acute Care Surg.

[316] Lier H, Krep H, Schroeder S, Stuber F (2008). Preconditions of haemostasis in trauma: a review. The influence of acidosis, hypocalcaemia, anaemia, and hypothermia on functional haemostasis in trauma. J Trauma.

[317] Hall C, Nagengast AK, Knapp C, Behrens B, Dewey EN, Goodman A, Bommiasamy A, Schreiber M (2021). Massive transfusions and severe hypocalcaemia: an opportunity for monitoring and supplementation guidelines. Transfusion.

[318] Vasudeva M, Mathew JK, Fitzgerald MC, Cheung Z, Mitra B (2020). Hypocalcaemia and traumatic coagulopathy: an observational analysis. Vox Sang.

[319] Matthay ZA, Fields AT, Nunez-Garcia B, Patel MH, Cohen MJ, Callcut RA, Kornblith LZ (2020). Dynamic effects of calcium on in vivo and ex vivo platelet behaviour after trauma. J Trauma Acute Care Surg.

[320] Magnotti LJ, Bradburn EH, Webb DL, Berry SD, Fischer PE, Zarzaur BL, Schroeppel TJ, Fabian TC, Croce MA (2011). Admission ionized calcium levels predict the need for multiple transfusions: a prospective study of 591 critically ill trauma patients. J Trauma.

[321] Giancarelli A, Birrer KL, Alban RF, Hobbs BP, Liu-DeRyke X (2016). Hypocalcaemia in trauma patients receiving massive transfusion. J Surg Res.

[322] Ho KM, Leonard AD (2011). Concentration-dependent effect of hypocalcaemia on mortality of patients with critical bleeding requiring massive transfusion: a cohort study. Anaesth Intensive Care.

[323] Lehmann M, Wallbank AM, Dennis KA, Wufsus AR, Davis KM, Rana K, Neeves KB (2015). On-chip recalcification of citrated whole blood using a microfluidic herringbone mixer. Biomicrofluidics.

[324] Knudson MM, Cohen MJ, Reidy R, Jaeger S, Bacchetti P, Jin C, Wade CE, Holcomb JB (2011). Trauma, transfusions, and use of recombinant factor VIIa: a multicentre case registry report of 380 patients from the Western Trauma Association. J Am Coll Surg.

[325] Hauser CJ, Boffard K, Dutton R, Bernard GR, Croce MA, Holcomb JB, Leppaniemi A, Parr M, Vincent JL, Tortella BJ (2010). Results of the CONTROL trial: efficacy and safety of recombinant activated factor VII in the management of refractory traumatic haemorrhage. J Trauma.

[326] Simpson E, Lin Y, Stanworth S, Birchall J, Doree C, Hyde C (2012). Recombinant factor VIIa for the prevention and treatment of bleeding in patients without haemophilia. Cochrane Database Syst Rev.

[327] DeLoughery EP, Lenfesty B, DeLoughery TG (2013). The use of recombinant factor VIIa in warfarin patients with traumatic brain injury: a retrospective case-control study. Blood Coagul Fibrinolysis.

[328] Lombardo S, Millar D, Jurkovich GJ, Coimbra R, Nirula R (2018). Factor VIIa administration in traumatic brain injury: an AAST-MITC propensity score analysis. Trauma Surg Acute Care Open.

[329] Perel P, Roberts I, Shakur H, Thinkhamrop B, Phuenpathom N, Yutthakasemsunt S (2010). Haemostatic drugs for traumatic brain injury. Cochrane Database Syst Rev.

[330] Levi M, Levy JH, Andersen HF, Truloff D (2010). Safety of recombinant activated factor VII in randomised clinical trials. N Engl J Med.

[331] O'Connell KA, Wood JJ, Wise RP, Lozier JN, Braun MM (2006). Thromboembolic adverse events after use of recombinant human coagulation factor VIIa. JAMA.

[332] Wirtz MR, Schalkers DV, Goslings JC, Juffermans NP (2020). The impact of blood product ratio and procoagulant therapy on the development of thromboembolic events in severely injured haemorrhaging trauma patients. Transfusion.

[333] Levi M, Eerenberg E, Kamphuisen PW (2011). Bleeding risk and reversal strategies for old and new anticoagulants and antiplatelet agents. J Thromb Haemost.

[334] Dowlatshahi D, Butcher KS, Asdaghi N, Nahirniak S, Bernbaum ML, Giulivi A, Wasserman JK, Poon MC, Coutts SB (2012). Canadian PCC registry (CanPro) investigators: poor prognosis in warfarin-associated intracranial haemorrhage despite anticoagulation reversal. Stroke.

[335] Edavettal M, Rogers A, Rogers F, Horst M, Leng W (2014). Prothrombin complex concentrate accelerates international normalized ratio reversal and diminishes the extension of intracranial haemorrhage in geriatric trauma patients. Am Surg.

[336] Fang MC, Go AS, Chang Y, Hylek EM, Henault LE, Jensvold NG, Singer DE (2007). Death and disability from warfarin-associated intracranial and extracranial haemorrhages. Am J Med.

[337] Kuramatsu JB, Gerner ST, Schellinger PD, Glahn J, Endres M, Sobesky J, Flechsenhar J, Neugebauer H, Jüttler E, Grau A (2015). Anticoagulant reversal, blood pressure levels, and anticoagulant resumption in patients with anticoagulation-related intracerebral haemorrhage. JAMA.

[338] Steiner T, Poli S, Griebe M, Hüsing J, Hajda J, Freiberger A, Bendszus M, Bösel J, Christensen H, Dohmen C (2016). Fresh frozen plasma versus prothrombin complex concentrate in patients with intracranial haemorrhage related to vitamin K antagonists (INCH): a randomised trial. Lancet Neurol.

[339] Hunt BJ, Levi M (2018). Urgent reversal of vitamin K antagonists. BMJ.

[340] Margraf DJ, Seaburg S, Beilman GJ, Wolfson J, Gipson JC, Chapman SA (2020). Propensity score adjusted comparison of three-factor versus four-factor prothrombin complex concentrate for emergent warfarin reversal: a retrospective cohort study. BMC Emerg Med.

[341] Brekelmans MPA, van Ginkel K, Daams JG, Hutten BA, Middeldorp S, Coppens M (2017). Benefits and harms of 4-factor prothrombin complex concentrate for reversal of vitamin K antagonist associated bleeding: a systematic review and meta-analysis. J Thromb Thrombolysis.

[342] Dentali F, Ageno W, Crowther M (2006). Treatment of coumarin-associated coagulopathy: a systematic review and proposed treatment algorithms. J Thromb Haemost.

[343] Peyko V, Shams D, Urbanski R, Noga J (2019). 4-factor prothrombin complex concentrate administration via intraosseous access for urgent reversal of warfarin. J Emerg Med.

[344] Dezee KJ, Shimeall WT, Douglas KM, Shumway NM, O'Malley PG (2006). Treatment of excessive anticoagulation with phytonadione (vitamin K): a meta-analysis. Arch Intern Med.

[345] Britt RB, Brown JN (2018). Characterising the severe reactions of parenteral vitamin K1. Clin Appl Thromb Hemost.

[346] Mangram A, Oguntodu OF, Dzandu JK, Hollingworth AK, Hall S, Cung C, Rodriguez J, Yusupov I, Barletta JF (2016). Is there a difference in efficacy, safety, and cost-effectiveness between 3-factor and 4-factor prothrombin complex concentrates among trauma patients on oral anticoagulants?. J Crit Care.

[347] Pernod G, Albaladejo P, Godier A, Samama CM, Susen S, Gruel Y, Blais N, Fontana P, Cohen A, Llau JV (2013). Management of major bleeding complications and emergency surgery in patients on long-term treatment with direct oral anticoagulants, thrombin or factor-Xa inhibitors: proposals of the working group on perioperative haemostasis (GIHP)-March 2013. Arch Cardiovasc Dis.

[348] Willekens G, Studt J, Mendez A, Alberio L, Fontana P, Wuillemin WA, Schmidt A, Graf L, Gerber B, Bovet C (2021). A universal anti-Xa assay for rivaroxaban, apixaban, and edoxaban measurements: method validation, diagnostic accuracy and external validation. Br J Haematol.

[349] Pavoni V, Gianesello L, Conti D, Ballo P, Dattolo P, Prisco D, Gorlinger K (2022). "In less than no time": feasibility of rotational thromboelastometry to detect anticoagulant drugs activity and to guide reversal therapy. J Clin Med.

[350] Seyve L, Richarme C, Polack B, Marlu R (2018). Impact of four direct oral anticoagulants on rotational thromboelastometry (ROTEM). Int J Lab Hematol.

[351] Connolly SJ, Milling TJ, Eikelboom JW, Gibson CM, Curnutte JT, Gold A, Bronson MD, Lu G, Conley PB, Verhamme P (2016). Andexanet alfa for acute major bleeding associated with factor Xa inhibitors. N Engl J Med.

[352] Cohen AT, Lewis M, Connor A, Connolly SJ, Yue P, Curnutte J, Alikhan R, MacCallum P, Tan J, Green L (2022). Thirty-day mortality with andexanet alfa compared with prothrombin complex concentrate therapy for life-threatening direct oral anticoagulant-related bleeding. J Am Coll Emerg Physicians Open.

[353] Demchuk AM, Yue P, Zotova E, Nakamya J, Xu L, Milling TJ, Ohara T, Goldstein JN, Middeldorp S, Verhamme P (2021). Haemostatic efficacy and anti-FXa (factor Xa) reversal with andexanet alfa in intracranial haemorrhage: ANNEXA-4 substudy. Stroke.

[354] Parsels KA, Seabury RW, Zyck S, Miller CD, Krishnamurthy S, Darko W, Probst LA, Latorre JG, Cwikla GM, Feldman EA (2022). Andexanet alfa effectiveness and safety versus four-factor prothrombin complex concentrate (4F-PCC) in intracranial haemorrhage while on apixaban or rivaroxaban: a single-centre, retrospective, matched cohort analysis. Am J Emerg Med.

[355] Benz AP, Xu L, Eikelboom JW, Middeldorp S, Milling TJ Jr, Crowther M, Yue P, Conley P, Lu G, Connolly SJ et al. Andexanet alfa for specific anticoagulation reversal in patients with acute bleeding during treatment with edoxaban. Thromb Haemost. 2022. Preprint. https://pubmed.ncbi.nlm.nih.gov/34996121/. 10.1055/s-0041-1740180 PMC9251710 34996121

[356] Bourdin M, Perrotin D, Mathieu O, Herve T, Depasse F, Lu G, Conley PB, Contant G (2021). Measuring residual anti-Xa activity of direct factor Xa inhibitors after reversal with andexanet alfa. Int J Lab Hematol.

[357] Hormese M, Littler A, Doane B, Glowacki N, Khimani A, Vivacqua N, Rudenberg K (2021). Comparison of high- and low-dose 4-factor prothrombin complex concentrate for the emergent reversal of oral factor Xa inhibitors. J Thromb Thrombolysis.

[358] Glund S, Stangier J, Schmohl M, Gansser D, Norris S, van Ryn J, Lang B, Ramael S, Moschetti V, Gruenenfelder F (2015). Safety, tolerability, and efficacy of idarucizumab for the reversal of the anticoagulant effect of dabigatran in healthy male volunteers: a randomised, placebo-controlled, double-blind phase 1 trial. Lancet.

[359] Pollack CV, Reilly PA, Eikelboom J, Glund S, Verhamme P, Bernstein RA, Dubiel R, Huisman MV, Hylek EM, Kamphuisen PW (2015). Idarucizumab for dabigatran reversal. N Engl J Med.

[360] Athavale A, Jamshidi N, Roberts DM (2020). Incomplete responses to the recommended dose of idarucizumab: a systematic review and pharmacokinetic analysis. Clin Toxicol (Phila).

[361] Eikelboom JW, Quinlan DJ, van Ryn J, Weitz JI (2015). Idarucizumab: the antidote for reversal of dabigatran. Circulation.

[362] Yang Z, Ni J, Long Z, Kuang L, Gao Y, Tao S (2020). Is hip fracture surgery safe for patients on antiplatelet drugs and is it necessary to delay surgery? A systematic review and meta-analysis. J Orthop Surg Res.

[363] Fiorelli EM, Bozzano V, Bonzi M, Rossi SV, Colombo G, Radici G, Canini T, Kurihara H, Casazza G, Solbiati M (2020). Incremental risk of intracranial haemorrhage after mild traumatic brain injury in patients on antiplatelet therapy: systematic review and meta-analysis. J Emerg Med.

[364] Colombo G, Bonzi M, Fiorelli E, Jachetti A, Bozzano V, Casazza G, Solbiati M, Costantino G (2021). Incidence of delayed bleeding in patients on antiplatelet therapy after mild traumatic brain injury: a systematic review and meta-analysis. Scand J Trauma Resusc Emerg Med.

[365] Cheng L, Cui G, Yang R (2022). The impact of preinjury use of antiplatelet drugs on outcomes of traumatic brain injury: a systematic review and meta-analysis. Front Neurol.

[366] Thorn S, Güting H, Mathes T, Schäfer N, Maegele M (2019). The effect of platelet transfusion in patients with traumatic brain injury and concomitant antiplatelet use: a systematic review and meta-analysis. Transfusion.

[367] Alvikas J, Myers SP, Wessel CB, Okonkwo DO, Joseph B, Pelaez C, Doberstein C, Guillotte AR, Rosengart MR, Neal MD (2020). A systematic review and meta-analysis of traumatic intracranial haemorrhage in patients taking prehospital antiplatelet therapy: is there a role for platelet transfusions?. J Trauma Acute Care Surg.

[368] Yorkgitis BK, Tatum DM, Taghavi S, Schroeppel TJ, Noorbakhsh MR, Philps FH, Bugaev N, Mukherjee K, Bellora M, Ong AW (2022). Eastern Association for the Surgery of Trauma multicentre trial: comparison of pre-injury antithrombotic use and reversal strategies among severe traumatic brain injury patients. J Trauma Acute Care Surg.

[369] Lokhandwala AM, Asmar S, Khurrum M, Chehab M, Bible L, Castanon L, Ditillo M, Joseph B (2021). Platelet transfusion after traumatic intracranial haemorrhage in patients on antiplatelet agents. J Surg Res.

[370] Brogi E, Corbella D, Coccolini F, Gamberini E, Russo E, Agnoletti V, Forfori F (2020). The role of platelet transfusions after intracranial haemorrhage in patients on antiplatelet agents: a systematic review and meta-analysis. World Neurosurg.

[371] Jehan F, Zeeshan M, Kulvatunyou N, Khan M, O'Keeffe T, Tang A, Gries L, Joseph B (2019). Is there a need for platelet transfusion after traumatic brain injury in patients on P2Y12 inhibitors?. J Surg Res.

[372] Geerts WH, Code KI, Jay RM, Chen E, Szalai JP (1994). A prospective study of venous thromboembolism after major trauma. N Engl J Med.

[373] Shalhoub J, Lawton R, Hudson J, Baker C, Bradbury A, Dhillon K, Everington T, Gohel MS, Hamady Z, Hunt BJ (2020). Graduated compression stockings as adjuvant to pharmaco-thromboprophylaxis in elective surgical patients (GAPS study): randomised controlled trial. BMJ.

[374] Dennis M, Sandercock P, Reid J, Graham C, Forbes J, Murray G, CLOTS (Clots in Legs or sTockings after Stroke) Trials Collaboration (2013). Effectiveness of intermittent pneumatic compression in reduction of risk of deep vein thrombosis in patients who have had a stroke (CLOTS 3): a multicentre randomised controlled trial. Lancet.

[375] Kakkos S, Kirkilesis G, Caprini JA, Geroulakos G, Nicolaides A, Stansby G, Reddy DJ (2022). Combined intermittent pneumatic leg compression and pharmacological prophylaxis for prevention of venous thromboembolism. Cochrane Database Syst Rev.

[376] Alhazzani W, Lim W, Jaeschke RZ, Murad MH, Cade J, Cook DJ (2013). Heparin thromboprophylaxis in medical-surgical critically ill patients: a systematic review and meta-analysis of randomised trials. Crit Care Med.

[377] Gaitanidis A, Breen KA, Christensen MA, Saillant NN, Kaafarani HMA, Velmahos GC, Mendoza AE (2021). Low-molecular weight heparin is superior to unfractionated heparin for elderly trauma patients. J Surg Res.

[378] Ko A, Harada MY, Barmparas G, Chung K, Mason R, Yim DA, Dhillon N, Margulies DR, Gewertz BL, Ley EJ (2016). Association between enoxaparin dosage adjusted by anti-factor Xa trough level and clinically evident venous thromboembolism after trauma. JAMA Surg.

[379] Singer GA, Riggi G, Karcutskie CA, Vaghaiwalla TM, Lieberman HM, Ginzburg E, Namias N, Lineen EB (2016). Anti-Xa-guided enoxaparin thromboprophylaxis reduces rate of deep venous thromboembolism in high-risk trauma patients. J Trauma Acute Care Surg.

[380] Spano PJ, Shaikh S, Boneva D, Hai S, McKenney M, Elkbuli A (2020). Anticoagulant chemoprophylaxis in patients with traumatic brain injuries: a systematic review. J Trauma Acute Care Surg.

[381] Ho KM, Rao S, Honeybul S, Zellweger R, Wibrow B, Lipman J, Holley A, Kop A, Geelhoed E, Corcoran T (2019). A multicentre trial of vena cava filters in severely injured patients. N Engl J Med.

[382] Shariff M, Kumar A, Adalja D, Doshi R (2021). Inferior vena cava filters reduce symptomatic but not fatal pulmonary emboli after major trauma: a meta-analysis with trial sequential analysis. Eur J Trauma Emerg Surg.

[383] Brolliar SM, Moore M, Thompson HJ, Whiteside LK, Mink RB, Wainwright MS, Groner JI, Bell MJ, Giza CC, Zatzick DF (2016). A qualitative study exploring factors associated with provider adherence to severe paediatric traumatic brain injury guidelines. J Neurotrauma.

[384] Harwayne-Gidansky I, Askin G, Fein DM, McNamara C, Duncan E, Delaney K, Greenberg J, Mojica M, Clapper T, Ching K (2022). Effectiveness of a simulation curriculum on clinical application: a randomised educational trial. Simul Healthc.

[385] Kaserer A, Rössler J, Braun J, Farokhzad F, Pape HC, Dutkowski P, Plass A, Horisberger T, Volbracht J, Manz MG (2019). Impact of a patient blood management monitoring and feedback programme on allogeneic blood transfusions and related costs. Anaesthesia.

[386] Coggins A, Zaklama R, Szabo RA, Diaz-Navarro C, Scalese RJ, Krogh K, Eppich W (2021). Twelve tips for facilitating and implementing clinical debriefing programmes. Med Teach.

[387] Dewolf P, Clarebout G, Wauters L, Van Kerkhoven J, Verelst S (2021). The effect of teaching nontechnical skills in advanced life support: a systematic review. AEM Educ Train.

[388] Park C, Grant J, Dumas RP, Dultz L, Shoultz TH, Scott DJ, Luk S, Abdelfattah KR, Cripps MW (2020). Does simulation work? Monthly trauma simulation and procedural training are associated with decreased time to intervention. J Trauma Acute Care Surg.

[389] Lee JC, Rittenhouse K, Bupp K, Gross B, Rogers A, Rogers FB, Horst M, Estrella L, Thurmond J (2015). An analysis of brain trauma foundation traumatic brain injury guideline compliance and patient outcome. Injury.

[390] Merck LH, Yeatts SD, Silbergleit R, Manley GT, Pauls Q, Palesch Y, Conwit R, Le Roux P, Miller J, Frankel M (2019). The effect of goal-directed therapy on patient morbidity and mortality after traumatic brain injury: results from the progesterone for the treatment of traumatic brain injury III clinical trial. Crit Care Med.

[391] Stein P, Spahn GH, Müller S, Zollinger A, Baulig W, Brüesch M, Seifert B, Spahn DR (2017). Impact of city police layperson education and equipment with automatic external defibrillators on patient outcome after out of hospital cardiac arrest. Resuscitation.

