# Supplementary material for: The European guideline on management of major bleeding and coagulopathy following trauma: sixth edition
Source: Crit Care. 2023 Mar 1;27:80. doi: 10.1186/s13054-023-04327-7 (PMC9977110; doi:10.1186/s13054-023-04327-7)
Supplement: Supplementary file 1 — Additional file 1. Summary of PICOs. [file 13054_2023_4327_MOESM1_ESM.pdf]

The European guideline on management of major bleeding and coagulopathy following trauma: Sixth edition

Additional file 1: Summary of PICOs

PICO	Population (P)	Intervention (I)	Comparison (C)	Search bundle
**PICO 1**	• All adult trauma patients with severe bleeding<br>• Need a corporal cavity (surgical) to collect autologous blood	• Use of cell salvage during resuscitation to collect and retransfuse washed autologous blood	• No use of cell salvage and autologous transfusion during resuscitation. Use only allogeneic blood to treat severe blood loss and anaemia	8
**PICO 2***	• Adults, severe trauma and haemorrhage	• Use a simulation educational tool, clinical debrief or after-action review during the rationale for the recommendations of the trauma guidelines<br>• Clinical debriefing topics following experiences with trauma patients: decision making, communication, resource utilisation, space, equipment, environmental, leadership, situational awareness, teamwork<br>• Assessment tools for cognitive biases and personality traits and their potential impact on physicians' decisions, medical errors and patient outcomes	• Any quality assurance reporting from facilitation strategies: debriefings or actions reviews, standard care or no simulation, standard care	11
**PICO 3**	Adult trauma patients:<br>• with severe or multiple injuries<br>• with multiple injuries<br>• with severe injuries<br>• with polytrauma<br>• with (acute) trauma haemorrhage<br>• with haemorrhagic shock<br>• with severe bleeding<br>• with ongoing haemorrhage<br>• with an injury ISS ≥16	• Transportation to trauma centre or level 1 trauma centre<br>• Minimize time between injury and bleeding control<br>• Time between arrival at hospital and start of emergency laparotomy <60 min<br>• Time between arrival at hospital and start of first red blood cell	• Transportation to a non-trauma centre or a non-level 1 trauma centre<br>• Standard care, no effort to optimise bleeding control time<br>• Time between arrival at hospital and start of emergency laparotomy <60 min	9

		(RBC) transfusion <60 min if transfused<br>• Time between arrival at hospital and packing of pelvis <60 min<br>• Time between arrival at hospital and start of any emergency surgery <60 min	• Time between arrival at hospital and start of first RBC transfusion <60 min if transfused<br>• Time between arrival at hospital and packing of pelvis <60 min<br>• Time between arrival at hospital and start of any emergency surgery ≥60 min	
**PICO 4**	• Trauma patients with severe- or life-threatening bleeding from extremity injuries	• Application of tourniquet<br>• Application of a pelvic binder	• No tourniquet when compression<br>• No use of pelvic binder<br>• Standard care	2
**PICO 5**	• All adult trauma patients:<br>• with trauma haemorrhage (blood loss >2 L)<br>• with bleeding (blood loss >2 L)<br>• with haemorrhagic shock (systolic blood pressure <90 mmHg)<br>• with haemorrhagic shock (shock index >0.9)<br>• with multiple injuries<br>• with penetrating injuries<br>• with blunt injuries	• Assessment of mechanism of injury (high velocity/speed, high energy, fall height)<br>• Assessment of magnitude of injury (e.g., through injury severity core [ISS])<br>• Assessment of anatomical injury (abbreviated injury scale [AIS]; all body regions ≥2)<br>• Assessment of the shock-index (SI)	• Assessment of mechanism of injury<br>• No assessment of magnitude of injury<br>• No assessment of anatomical injury<br>• No assessment of the shock-index (SI)<br>• Assessment of heart rate alone<br>• Assessment of systolic blood pressure alone	10
**PICO 6**	• Adult trauma patients in severe shock and/or with an obvious source of bleeding and/or extremis<br><br>*Severe shock is approximatively a blood loss of more than 40% of the blood volume (ATLS® classification) or a base deficit >10 mmol [1]. An extremis is the patient who is “trying to die” or the last minute or minutes before the heart stops due to severe bleeding.	• Immediate intervention, surgical and/or angiographic to control bleeding	• Delayed bleeding control/intervention	1

**PICO 7**	• Trauma patients, severe injury with or without haemodynamic stability	• Imaging for identify major sources of acute blood loss following traumatic injury: computed tomography (CT scan), ultrasonography, conventional radiology, whole body, multi-slice, delayed-phase or contrast medium-enhanced CT, torso CT scan/CT scan of the chest, abdominal cavity and pelvic ring<br>• CT scanners integrated into modern resuscitation units and emergency departments as diagnostic measure during the primary survey, pre-hospital primary survey and treatment by trained and experienced emergency personnel and short transportation times<br>• Proximity of the CT scanner to the resuscitation room in the emergency department <50 m<br>• Transport to CT scan room<br>• Transfer to CT scan closely monitored with continued resuscitation measures<br>• Two trained persons trauma team for transfer to CT scan<br>• A doctor with training in airway management, advanced cardiac life support and experience in critical care should accompany all patients who are unstable<br>• The doctor should be experienced and competent in transport medicine, and the other attendant should be a suitable nurse,	• Inattention to or omission of CT scan or only conventional radiology, ultrasonography, CT scan of the chest, abdominal cavity and pelvic ring<br>• Ultrasonography, abdominal ultrasound, chest and limb radiology<br>• No CT scan, ultrasonography and conventional radiographic imaging in emergency department<br>• No pre-hospital completed primary survey and treatment<br>• CT scanner placed at distance >50 m from emergency department<br>• No CT scan<br>• CT scan in emergency department<br>• Ultrasonography<br>• Regular transfer<br>• Monitored transfer without treatment<br>• Regular transfer with stretcher-bears<br>• Emergent surgical intervention<br>• No imaging in pre-hospital	3

		paramedic or technician familiar with intensive care procedures<br>• Non-surgical interventions<br>• Proximity of the CT scanner to the resuscitation room in the emergency department <50 m<br>• Pre-hospital extended focused assessment with sonography in trauma (eFAST)		
**PICO 8**	Adult trauma patients:<br>• with torso trauma and/or haemodynamic instability<br>• with intra-abdominal bleeding<br>• with torso trauma<br>• with abdominal trauma<br>• with polytrauma<br>• with multiple injuries<br>• with penetrating torso injuries<br>• with blunt torso injuries	• Focussed assessment with sonography in trauma (FAST)<br>• eFAST<br>• Emergency ultrasound<br>• Emergency room ultrasound<br>• Contrast-enhanced whole-body CT (WBCT)<br>• Early* imaging (WBCT)<br>*early: immediately or within one hour upon hospital/emergency room/trauma bay admission	• No focussed assessment with sonography in trauma (FAST)<br>• No eFAST<br>• No ultrasound<br>• No contrast-enhanced whole-body CT (WBCT)<br>• No early imaging (WBCT)	3
**PICO 9**	Trauma patients (no age restriction):<br>• with bleeding prehospital/at site/ intrahospital<br>• with/without anticoagulants or antiplatelet agents	• Conventional clotting tests (activated partial thromboplastin time [APTT] / prothrombin time [PT]/ fibrinogen/ international normalized ratio [INR]/ PT ratio)<br>• Viscoelastic tests (thromboelastography [TEG]/ rotational thromboelastometry [ROTEM]/ Sonoclot)<br>• Viscoelastic measures of fibrinogen (e.g., functional fibrinogen/ fibrin-based extrinsically activated test with tissue factor and the platelet inhibitor cytochalasin D [FIBTEM])<br>• Point of care tests - all of the above	• Conventional clotting tests (APTT/PT/fibrinogen/INR)<br>• Viscoelastic tests (TEG/ROTEM/Sonoclot) - including fibrinogen measures (functional fibrinogen and FIBTEM)<br>• Point of care tests - all of the above	4

**PICO 10**	• Adult (>18 years old) trauma patient with and without traumatic intracranial haemorrhage (TICH) including patients with pre-injury antiplatelet medication*<br><br>*It is preferrable to look at aspirin, clopidogrel, ticagrelor, prasugrel, dual antiplatelet medication and dipyridamole as mentioned in the PICO, but many articles mention “antiplatelet agents”	• Point-of-care platelet function monitoring<br>• Whole-blood multiple electrode impedance aggregometry<br>• Platelet function analyser (PFA-100®)<br>• Platelet reactivity assay (e.g., VerifyNow®)<br>• Vasodilator-stimulated phosphoprotein<br>• Viscoelastic devices with channels for measuring platelet function. There is no consensus on the definition or standard threshold of platelet function monitoring	• No platelet function monitoring<br>• Standard laboratory platelet function monitoring#<br>• Coagulation monitoring by viscoelastic tests<br>• Standard laboratory coagulation monitoring<br><br>#Light transmission aggregometry is regarded as the gold standard of platelet function testing and is still the most used test for the identification and diagnosis of platelet function defects	4
**PICO 11**	• Bleeding trauma patients with or without traumatic brain injury	• Permissive hypotension<br>• Restrictive volume replacement<br>• Isotonic crystalloid solution<br>• Vasopressors in severe hypotension<br>• Inotropic agents in myocardial dysfunction<br>• Restrictive transfusions of erythrocytes<br>• Aiming for normothermia	• Normotension<br>• Liberal volume replacement<br>• Saline solutions, hypo- or hypertonic solutions, gelatine solutions, starch solutions<br>• No vasopressors in life-threatening hypotension<br>• No inotropic agents in myocardial dysfunction<br>• Liberal transfusions of erythrocytes<br>• Aiming for hypothermia	5
**PICO 12**	• All trauma patients<br>• Severely injured patients presenting deep haemorrhagic shock, signs of bleeding and coagulopathy, Ph <7.2, lactate level > 5, coagulopathy, massive blood loss and low body temperature	• Damage control Surgery and resuscitation<br>• Abbreviated laparotomy<br>• Mass transfusion protocol<br>• Intensive care	• Definitive repair (no damage control surgery)<br>• No mass transfusion protocol<br>• Traditional volume replacement and transfusion strategy<br>• Definitive surgical repair of all abdominal injuries<br>• Standard care	1

**PICO 13**	• Trauma patients with pelvic ring disruption and haemorrhagic shock	• Pelvic closure, haemorrhagic control with packing, embolization, vascular surgery	• No surgical intervention or interventional radiology<br>• Standard care<br>• Conservative treatment	2
**PICO 14**	Trauma patients:<br>• with intrathoracic and or/intra-abdominal visceral bleeding<br>• bleeding from soft tissue or bone in the pre-hospital setting	• Application of local haemostatic products on bleeding surfaces	• No application of those products or application of other non-haemostatic agents<br>• Standard care	6
**PICO 15**	• All ages with trauma at the scene/or in hospital	• Tranexamic acid/anti-fibrinolytic drugs	• No tranexamic acid/anti-fibrinolytic drugs	6
**PICO 16**	• Adult trauma patients with expected massive haemorrhage or major injury	• Fresh frozen plasma (FFP), cryoprecipitate, fibrinogen and factor XIII administration by trauma teams, anaesthesiologists and surgeons<br>• Liberal and prophylactic FFP administration<br>• FFP in a fixed ratio of FFP:RBC (e.g., 1:1 or 1:2) or FFP:RBC:platelet (e.g., 1:1:1)<br>• FFP transfusion for fibrinogen depletion or hypofibrinogenaemia<br>• FFP+RBC as initial management<br>• Fibrinogen concentrate and/or prothrombin concentrate and/or factor XIII<br>• Freeze-dried plasma<br>• Coagulation monitoring and transfusion triggers<br>• Transfusion based on viscoelastic methods and visco haemostatic assays<br>• Transfusion based on rotational thromboelastometry (ROTEM) and thromboelastography (TEG)	• Standard care administered by trauma teams, anaesthesiologists and surgeons<br>• No liberal or prophylactic FFP administration<br>• Liberal transfusion regime using FFP<br>• Fibrinogen concentrate<br>• Substitution for fibrinogen depletion or hypofibrinogenaemia<br>• RBC only as initial management<br>• FFP and cryoprecipitate and platelet transfusion<br>• FFP and cryoprecipitate and platelet transfusion instead of freeze dried<br>• Standard care<br>• Standard laboratory and coagulation tests (INR, PT and APTT)<br>• No point of care tests<br>• No pre-defined transfusion triggers and standard care<br>• No algorithm transfusion management	8

		• FFP and coagulation factor therapy (fibrinogen concentrate, prothrombin concentrate) based on pre-specified transfusion triggers<br>• Algorithm based coagulation and transfusion management<br>• Transfusion triggers<br>• Prophylactic tranexamic acid and antifibrinolytics<br>• Fibrinogen concentrate/cryo<br>• Pre-hospital, in-hospital, surgical procedure and post-procedure<br>• Fibrinogen/cryo administration<br>• Fibrinogen concentrate<br>• Fibrinogen concentrate based on viscoelastic/visco haemostatic assays and algorithm<br>• Ionized calcium measurement and monitoring and calcium administration<br>• Point of care calcium measurement<br>• Calcium chloride and/or calcium gluconate administration	• Fixed ratio coagulation and transfusion treatment<br>• No prophylactic tranexamic acid and antifibrinolytics<br>• Standard care, placebo or saline vs cryoprecipitate vs FFP<br>• Fibrinogen concentrate based on transfusion triggers<br>• Standard laboratory tests<br>• No calcium administration	
**PICO 17**	Adult trauma patients:<br>• with bleeding<br>• with (acute) trauma haemorrhage<br>• with haemorrhagic shock<br>• with severe bleeding<br>• with ongoing haemorrhage<br>• with acute traumatic coagulopathy<br>• with haemostatic abnormalities<br>• with acquired coagulopathy<br>• with concern for coagulopathy<br>• with traumatic brain injury and bleeding with or without acute coagulopathy	• Goal-directed therapeutic treatment algorithm<br>• Viscoelastic haemostatic assay-based treatment algorithm/transfusion strategy<br>• Conventional coagulation assay-based treatment algorithm<br>• Point-of-care testing-based algorithm<br>• Early coagulation monitoring<br>• Early coagulation assessment<br>• Point-of-care testing monitoring	• No therapy<br>• No monitoring<br>• No assessment<br>• No goal-directed therapeutic treatment algorithm<br>• No viscoelastic haemostatic assay-based treatment algorithm or transfusion strategy<br>• Conventional coagulation assay-based treatment algorithm<br>• Pont-of-care testing-based algorithm<br>• Any other transfusion strategy	13

	All adult traumatic brain injury (TBI) patients:<br>• with intracranial haemorrhage<br>• with intracranial bleeding<br>• with ongoing intracranial bleeding<br>• with progression of intracranial haemorrhage<br>• with bleeding progression<br>• with expansion of haemorrhagic lesions<br>• with delayed intracranial haemorrhage<br>• with haemostatic abnormalities<br>• with acquired coagulopathy<br>• with concern for coagulopathy			
**PICO 18**	Adult (>18 years old) trauma patients:<br>• with major bleeding<br>• with TBI<br>• with thrombocytopenia and/or coagulopathy<br>• with intracranial haemorrhage (TICH)	• Platelet transfusion<br>• Fresh, room-temperature and ABO-compatible platelets<br>• Old platelets<br>• ABO-identical platelets<br>• ABO-incompatible platelets<br>• Cold-sored platelets<br>• Pathogen-reduced platelets<br>• Single-unit platelet<br>• Apheresis platelets<br>• Buffy coat platelets<br>• Platelet-rich plasma<br>• Early platelet transfusion based on ratio of blood products<br>• 1:1:1 ratio of blood products<br>• 2:1:1 ratio of blood products<br>• Other ratios of blood products	• Alternatives to platelet transfusion<br>• No platelet transfusion<br>• Tranexamic acid<br>• Fibrinogen/cryoprecipitate<br>• Desmopressin<br>• Synthetic platelets<br>• Plasma transfusion<br>• Alternatives to platelets given in fixed ratio with red blood cells and plasma<br>• Platelet transfusion based on viscoelastic/visco haemostatic tests<br>• Platelet transfusion based on platelet count<br>• Platelet transfusion based on platelet function tests<br>• No platelet transfusion	8
**PICO 19**	• Bleeding trauma patient	• Administration of recombinant activated coagulation factor VII	• No administration of recombinant activated coagulation factor VII	6

	• Bleeding patient undergoing surgery due to trauma<br>• Bleeding patient on extracorporeal membrane oxygenation support<br>• Bleeding patient undergoing elective major surgery			
**PICO 20**	• All ages with trauma, at scene or in hospital, including traumatic head injury<br>• Patients taking vitamin K antagonists/ direct-acting oral anticoagulants (DOAC)/anti-platelet agents	• Reversal of vitamin K-dependent oral anticoagulants<br>• Reversal of dabigatran anticoagulation with idarucizumab<br>• Reversal of anticoagulation due to apixaban, edoxaban or rivaroxaban with andexanet alfa<br>• Anticoagulation monitoring based on ROTEM, TEG, visco haemostatic and viscoelastic test	• No reversal therapy<br>• Reversal of dabigatran anticoagulation with prothrombin complex concentrate<br>• Reversal of anticoagulation due to apixaban, edoxaban or rivaroxaban with prothrombin concentrate<br>• Anticoagulation monitoring based on anti Xa activity, (diluted) thrombin time, PT and coagulation tests	7
**PICO 21**	Adult (>18 years old) trauma patients on antiplatelet agents:<br>• with major bleeding<br>• including patients with TBI<br>• including patients with thrombocytopenia<br>• including patients with intracranial bleeding<br>• (aspirin, clopidogrel, ticagrelor, prasugrel, dual antiplatelet medication, dipyridamole or the term antiplatelet agents)<br><br>Adult (> 18 years old) trauma patient:<br>• with major bleeding<br>• including patients with TBI	• Platelet transfusion<br>• Platelet transfusion based on platelet count<br>• Platelet transfusion based on platelet function tests<br>• Platelet transfusion based on viscoelastic/visco haemostatic tests<br>• Platelet transfusion based on ratios of blood products<br>• Platelet transfusion based on algorithm or transfusion trigger<br>• Pre-injury antiplatelet medication: aspirin, clopidogrel, ticagrelor and prasugrel<br>• Dual antiplatelet medication: dipyridamole	• Alternatives<br>• Tranexamic acid<br>• Desmopressin<br>• Recombinant factor VIIa<br>• No platelet transfusion<br>• Platelet transfusion based on target value/range<br>• No pre-injury antiplatelet medication<br>• Pre-injury oral anticoagulation (antivitamin K or DOAC)	8

	• including patients with thrombocytopenia<br>• including patients with intracranial bleeding			
**PICO 22**	• All ages (but mostly 16 years and above)<br>• In hospital<br>• Post-discharge<br>• Specifically, immediately after injury vs longer after injury	• Pharmacological thromboprophylaxis (unfractionated heparin/low molecular weight heparin/DOAC)<br>• Mechanical thromboprophylaxis<br>• Pneumatic compression<br>• Internal vena cava filter	• Mechanical thromboprophylaxis<br>• Pharmacological<br>• Thromboprophylaxis (another type)<br>• Combination mechanical and chemical thromboprophylaxis<br>• Stockings<br>• No thromboprophylaxis<br>• No internal vena cava filter	7
**PICO 23**	• Acute care medical trauma teams anaesthesiologists, trauma surgeons	• Active intervention to implement the guideline<br>• Repetitive educational activities<br>• Monitoring of guideline adherence<br>• Institutional quality program feedback<br>• Introduction of bundles, checklists and coagulation algorithms	• Standard care<br>• No active intervention	12
**PICO 24***	• Adult trauma patients with or without suspected bleeding<br>• ISS >15 and multiple injuries with/without haemodynamic instability (systolic blood pressure <90 mmHg; SI >0,9)<br>• Torso trauma (AIS abdomen >2) with/without haemodynamic instability (AIS abdomen <90 mmHg; SI >0,9)<br>• Abdominal trauma (AIS abdomen >2)<br>• Thoracic trauma (AIS thorax >2) and/or penetrating thoracic injuries and/or blunt thoracic injuries with/without and	• Point of care ultrasound<br>• Prehospital ultrasound	• No prehospital ultrasound	3

	haemodynamic instability (AIS abdomen <90 mmHg; SI >0,9)<br>• Pelvic injury (AIS pelvis >2) and/or penetrating pelvic injuries and/or blunt pelvic injuries<br>• Polytrauma (ISS >15)<br>• Penetrating and/or blunt torso injuries			
**PICO 25**	• Bleeding trauma patient	• Administration of blood/FFP transfusions or fibrinogen at prehospital setting	• No administration of blood transfusions at prehospital setting	8

*Omitted from the search strategy due to the volume of results.

REFERENCE

1. Mutschler M, Nienaber U, Brockamp T, Wafaisade A, Fabian T, Paffrath T, Bouillon B, Maegele M, TraumaRegister DGU: Renaissance of base deficit for the initial assessment of trauma patients: a base deficit-based classification for hypovolemic shock developed on data from 16,305 patients derived from the TraumaRegister DGU®. Crit Care 2013, 17(2):R42.
