# Supplementary material for: The European guideline on management of major bleeding and coagulopathy following trauma: sixth edition
Source: Crit Care. 2023 Mar 1;27:80. doi: 10.1186/s13054-023-04327-7 (PMC9977110; doi:10.1186/s13054-023-04327-7)
Supplement: Supplementary file 2 — Additional file 2. Search bundles, structured literature search strategies and results. [file 13054_2023_4327_MOESM2_ESM.pdf]

The European guideline on management of major bleeding and coagulopathy following trauma: Sixth edition

Additional file 2: Search bundles, structured literature search strategies and results

Additional file 2: Search bundles, structured literature search strategies and results

Table 1: Search strategy bundles based on PICOs.

Table 2: Search strategy queries used in Ovid MEDLINE for each search bundle.

Table 3: Search strategy queries used in CENTRAL via the Cochrane Library.

Table 4: Search strategy queries used in Epistemonikos.

Table 5: Database search results (number identified) by search bundle.

The European guideline on management of major bleeding and coagulopathy following trauma: Sixth edition

Additional file 2: Search bundles, structured literature search strategies and results

Table 1: Search strategy bundles based on PICOs.

Search bundle	Topic(s)	PICO(s)
Bundle 1	Initial resuscitation, triage, intubation, damage control etc.	16, 12
Bundle 2	Rapid control of fractures and wounds: tourniquets, packing, binder, fixation etc.	4, 13
Bundle 3	Imaging, ultrasound, CT scan etc.	7, 8, 24*
Bundle 4	Point of care tests, blood testing etc.	9, 10
Bundle 5	Hypotension, tissue oxygenation, fluid, temperature etc.	11
Bundle 6	Coagulation, haemostatics etc.	14, 15, 19
Bundle 7	Anticoagulation, thromboprophylaxis, stockings etc.	20, 22
Bundle 8	Transfusion, platelets, plasma etc.	1, 16, 18, 21, 25
Bundle 9	Time to treatment, transportation to treatment, etc.	3
Bundle 10	Injury severity, classification systems etc.	5
Bundle 11*	Simulation, teamwork, education, decision making, etc.*	2*
Bundle 12	Guidelines, bundles, quality control	17, 23

*Omitted from the search strategy due to the volume of results.

The European guideline on management of major bleeding and coagulopathy following trauma: Sixth edition

Additional file 2: Search bundles, structured literature search strategies and results

Table 2: Search strategy queries used in Ovid MEDLINE for each search bundle.

Search number	Query	Results 03 Dec 2021	Comments
1	exp "wounds and injuries"/	957,562	
2	(injur* or trauma*).tw,kf.	1,157,909	
3	1 or 2	1,698,932	
4	exp hemorrhage/	345,866	
5	(bleed* or h?emorrhag* or blood loss or coagulation disorder* or coagulopath* or disseminated intravascular coagulation or DIC).tw,kf.	539,868	
6	blood coagulation disorders/ or disseminated intravascular coagulation/	26,732	
7	4 or 5 or 6	688,046	
8	3 and 7	113,376	Narrow population
9	limit 8 to yr="2018 -current"	21,078	Narrow population limited to 2018–2021
10	exp resuscitation/	103,023	
11	exp intubation, intratracheal/	41,102	
12	triage/	13,701	
13	(resuscitat* or CPR or ventilat*).tw,kf.	253,664	
14	((tracheal or intratracheal or endotracheal) and intubation*).tw,kf.	22,517	
15	(triage or overtriage or undertriage).tw,kf.	21,735	
16	(((damage or urgen* or emergen*) adj2 control) or initial care).tw,kf.	5,333	
17	10 or 11 or 12 or 13 or 14 or 15 or 16	348,310	Bundle 1
18	tourniquets/	4,099	
19	exp fracture fixation/	66,241	
20	(tourniquet* or pelvi? binder* or pelvi? closure or pelvi? ring* or packing or emboli* or ((device* or wound*) adj3 compression*) or ((fracture* or internal* or external*) adj3 fixat*)).tw,kf.	227,289	
21	exp pelvic bones/	31,753	
22	pubic symphysis/	1,795	
23	sacroiliac joint/	4,332	
24	exp hip fractures/	26,386	
25	embolization, therapeutic/	34,410	

26	(pelvi? or hip? or pubic* or sacroiliac* or symphys* or rib or chest wall or ab).ti. and (fracture* or injur* or trauma* or disrupt* or instability).tw,kf.	34,120	
27	18 or 19 or 20 or 21 or 22 or 23 or 24 or 25 or 26	345,796	Bundle 2
28	*diagnostic imaging/	24,427	
29	exp tomography, X-ray computed/	466,370	
30	exp ultrasonography/	465,526	
31	angiography/	61,323	
32	((comput* adj2 tomograph*) or CT or ultrasonograph* or sonograph* or eFAST or early FAST or ultrasound* or ultra sound* or imaging).tw,kf.	1,624,967	
33	(angiogra* or angioemboli*).tw,kf.	217,395	
34	28 or 29 or 30 or 31 or 32 or 33	2,131,007	Bundle 3
35	hematologic tests/	9,795	
36	exp blood coagulation tests/	42,866	
37	point-of-care testing/	3,193	
38	(((point of care or clotting or platelet* or laborator* or coagulat* or h?ematocrit or h?emoglobin or h?ematolog*) adj6 (test or tests or testing or parameter* or monitoring or measur* or tool or tools)) or blood test* or POCT or (serum lactate or admission lactate or base deficit or base excess)).tw,kf.	217,588	
39	(APTT or activated partial thromboplastin time or thromboelastograph* or TEG or thromboelastometr* or ROTEM or Sonoclot or FIBTEM or multiple electrode impedance aggregometry or MEA or platelet function analyser or PFA-100 or platelet reactivity assay or VerifyNow* or vasodilator-stimulated phosphoprotein or VASP or D-dimer or early coagulation or conventional coagulation or CCT or viscoelastic* or VHA or visco h?emostatic* or viscoh?emostatic*).tw,kf.	63,678	
40	35 or 36 or 37 or 38 or 39	308,177	Bundle 4
41	exp blood pressure/	300,643	
42	hematocrit/	33,599	
43	exp hypotension/	28,881	
44	exp vasoconstrictor agents/	263,196	
45	exp fluid therapy/	21,337	
46	exp hypertonic solutions/	11,976	
47	exp isotonic solutions/	9,651	

48	exp crystalloid solutions/	3,883	
49	exp colloids/	138,909	
50	erythrocyte transfusion/	9,582	
51	exp hypothermia, induced/	21,304	
52	(blood pressure* or h?ematocrit or hypotension or vasopressor* or vasoconstrict* or inotropic agent* or inotropes or fluid therap* or fluid resuscitation* or volume resuscitation* or fluid replacement* or volume replacement* or rehydration therap* or sodium chlorid* or saline solution* or hypertonic solution* or isotonic solution* or hypotonic solution* or cr?stalloid* or colloid* or plasma substitut* or ringer* solution* or ringer* lactat* or blood substitute* or erythrocyte* or hypotherm* or balanced solution or Hartmann or plasmalyte).tw,kf.	747,618	
53	41 or 42 or 43 or 44 or 45 or 46 or 47 or 48 or 49 or 50 or 51 or 52	1,270,213	Bundle 5
54	exp blood coagulation/	61,055	
55	exp hemostasis/	117,428	
56	exp hemostatics/	146,171	
57	exp fibrinogen/	42,135	
58	exp antifibrinolytic agents/	29,596	
59	tranexamic acid/	4,242	
60	prothrombin/	10,867	
61	(coagula* or h?emosta* or fibrinogen* or antifibrinolytic* or anti fibrinolytic* or tranexamic acid* or prothrombin* or (blood adj1 clot*)).tw,kf.	238,684	
62	54 or 55 or 56 or 57 or 58 or 59 or 60 or 61	419,617	Bundle 6
63	exp anticoagulants/	232,340	
64	exp platelet aggregation inhibitors/	129,726	
65	exp factor Xa inhibitors/	8,624	
66	(anticoagula* or anti coagula* or DOAC* or antiplatelet* or anti platelet* or antithromb* or anti thromb* or anti factor Xa or antifactor Xa or ((platelet* or thrombin* or factor Xa) adj6 inhibitor*) or rivaroxaban or apixaban or edoxaban or betrixaban or dabigatran or andexanet alfa or heparin or TXA or idarucizumab or PCC or PCCs or prothrombin complex concentrate* or reversal or reversing or residual).tw,kf.	542,214	
67	intermittent pneumatic compression devices/	767	
68	stockings, compression/	1,658	

69	(thromboprophylaxis or (thrombosis adj1 prevent*) or intermittent pneumatic compression* or stocking* or (vena cava adj2 filter*)).tw,kf.	16,109	
70	63 or 64 or 65 or 66 or 67 or 68 or 69	767,746	Bundle 7
71	exp plasma/	29,373	
72	platelet transfusion/	7,553	
73	exp blood transfusion/	89,520	
74	blood platelets/	79,804	
75	calcium chloride/	7,609	
76	deamino arginine vasopressin/	4,266	
77	(plasma or FFP or erythrocyte* or red blood cell* or platelet* or thrombocyte* or blood transfusion* or blood product*).tw,kf.	1,346,061	
78	(transfusion adj2 (trigger* or mass* or management or requirement*)).tw,kf.	9,697	
79	(cryoprecipitate* or cryo or thrombocytopenia or thrombocytopaenia or calcium chloride or hypocalcemia or hypocalcaemia or desmopressin or PCC or PCCs or prothrombin complex concentrate* or rFVIIa or recombinant factor VIIa or (recombinant activated adj2 factor VII) or FXIII or factor XIII).tw,kf.	111,014	
80	operative blood salvage/	390	
81	(((cell or blood) adj3 salvage) or cell saver or autotransfus* or auto transfus* or (autologous adj3 blood adj3 (transfus* or retransfus*))).tw,kf.	4,939	
82	71 or 72 or 73 or 74 or 75 or 76 or 77 or 78 or 79 or 80 or 81	1,488,199	Bundle 8
83	time-to-treatment/	9,141	
84	*emergency service, hospital/	45,530	
85	trauma centers/	11,940	
86	(((trauma adj2 (care or centre* or centre* or unit* or facilit*)) and (transport* or transfer* or time or timing or designation)) or time to treatment*).tw,kf.	19,564	
87	83 or 84 or 85 or 86	83,186	Bundle 9
88	exp trauma severity indices/	36,512	
89	(((injury or trauma or shock) adj1 severity) or ISS or mechanism of injury or magnitude of injury or shock index or (assess* adj3 (high velocity or speed or high energy or fall height)) or ((shock* or ATLS or injur* or trauma*) adj3 classification*)).tw,kf.	35,229	

90	88 or 89	61,448	Bundle 10
91	practice guideline/	29,316	
92	practice guidelines as topic/	126,195	
93	exp clinical protocols/	180,188	
94	guideline adherence/	34,341	
95	decision making/	101,000	
96	patient care bundles/	1,082	
97	(guideline* or decision making or protocol* or checklist* or assessment tool* or ((treatment or care or patient) adj2 bundle*) or quality control or quality program*).tw,kf.	1,146,475	
98	((treat* or therap*) adj3 goal directed).tw,kf.	1,768	
99	((treat* or therap* or care) and algorithm*).tw,kf.	74,546	
100	91 or 92 or 93 or 94 or 95 or 96 or 97 or 98 or 99	1,497,207	Bundle 12
101	17 or 27 or 34 or 40 or 53 or 62 or 70 or 82 or 87 or 90 or 100	6,999,775	All bundle interventions
102	9 and 101	14,535	All bundles and narrow population
103	epidemiologic studies/	8,916	
104	observational study/	115,701	
105	exp cohort studies/	2,258,503	
106	exp case control studies/	1,257,879	
107	cross-sectional studies/	401,632	
108	clinical trial.pt.	532,553	
109	cohort*.ti,ab.	710,565	
110	((follow up or observational or uncontrolled or non randomi?ed or nonrandomi?ed or epidemiologic*) adj3 (study or studies or data)).ti,ab.	418,327	
111	((longitudinal or retrospective* or prospective* or cross sectional) and (study or studies or review or analys* or data)).ti,ab.	1,972,613	
112	controlled before-after studies/	668	
113	(before adj2 after adj2 (study or studies or data)).ti,ab.	8,564	
114	case control*.ti,ab.	142,716	
115	(cross sectional and (study or studies or review or analys* or data)).ti,ab.	398,999	
116	103 or 104 or 105 or 106 or 107 or 108 or 109 or 110 or 111 or 112 or 113 or 114 or 115	4,235,152	
117	randomised controlled trial.pt.	552,234	
118	controlled clinical trial.pt.	94,570	

119	randomi?ed.ti,ab.	700,684	
120	placebo.ab.	223,598	
121	drug therapy.fs.	2,410,323	
122	randomly.ab.	371,093	
123	trial.ti,ab.	672,443	
124	groups.ab.	2,279,685	
125	117 or 118 or 119 or 120 or 121 or 122 or 123 or 124	5,245,861	
126	meta-analysis/	148,121	
127	meta-analysis as topic/	20,570	
128	(meta analy* or metanaly* or metaanaly* or meta regression).ti,ab.	220,705	
129	((systematic* or evidence*) adj3 (review* or overview*)).ti,ab.	290,199	
130	(reference list* or bibliograph* or hand search* or manual search* or relevant journals).ab.	48,864	
131	(search strategy or search criteria or systematic search or study selection or data extraction).ab.	67,322	
132	(search* adj4 literature).ab.	80,559	
133	(medline or pubmed or cochrane or embase or scopus or web of science).ab.	274,509	
134	cochrane.jw.	15,740	
135	((multiple treatment* or indirect or mixed) adj2 comparison*).ti,ab.	3,313	
136	126 or 127 or 128 or 129 or 130 or 131 or 132 or 133 or 134 or 135	556,374	
137	practice guideline/	29,316	
138	practice guidelines as topic/	126,195	
139	(guideline* or guidance or consensus or recommendation*).ti,ab.	895,573	
140	137 or 138 or 139	956,213	
141	116 or 125 or 136 or 140	8,747,724	
142	exp animals/not humans.sh.	4,923,990	Excluding animals by MeSH term
143	141 not 142	7,973,449	Filter for study-types
144	102 and 143	8,217	Full result
145	9 and 17 and 143	1,290	Bundle 1 result
146	9 and 27 and 143	1,973	Bundle 2 result
147	9 and 34 and 143	2,703	Bundle 3 result
148	9 and 40 and 143	840	Bundle 4 result
149	9 and 53 and 143	1,195	Bundle 5 result

150	9 and 62 and 143	1,740	Bundle 6 result
151	9 and 70 and 143	1,586	Bundle 7 result
152	9 and 82 and 143	2,209	Bundle 8 result
153	9 and 87 and 143	559	Bundle 9 result
154	9 and 90 and 143	1,265	Bundle 10 result
155	9 and 100 and 143	1,743	Bundle 12 result
156	145 or 146 or 147 or 148 or 149 or 150 or 151 or 152 or 153 or 154 or 155	8,217	Full result

Additional file 2: Search bundles, structured literature search strategies and results

Table 3: Search strategy queries used in CENTRAL via the Cochrane Library.

Search number	Query	Results 03 Dec 2021
#1	MeSH descriptor: [Wounds and Injuries] explode all trees	27708
#2	(injur* or trauma*):ti,ab,kw	81064
#3	#1 or #2	90944
#4	MeSH descriptor: [Hemorrhage] explode all trees	15149
#5	(bleed* or haemorrhag* or hemorrhag* or “blood loss”):ti,ab,kw	76662
#6	#4 or #5	77921
#7	#3 and #6	7702
#8	MeSH descriptor: [Multiple Trauma] explode all trees	244
#9	(((multiple or severe* or serious* or penetrat* or blunt or torso or abdominal or pelvic or pelvis or ISS or critical* or major or "life threatening" or thoracic or abdominal or urological or skeletal or genitourinary) NEAR/4 (injur* or trauma*)) or polytrauma* or (poly NEXT trauma*) or (trauma* NEAR/2 injur*) or ((resuscitat* or shock) NEAR/10 (injur* or trauma*))):ti,ab,kw	14197
#10	#8 or #9	14207
#11	#7 or #10 with Publication Year from 2018 to 2021, in Trials	6268
#12	MeSH descriptor: [Resuscitation] explode all trees	5417
#13	MeSH descriptor: [Intubation, Intratracheal] explode all trees	4628
#14	MeSH descriptor: [Triage] explode all trees	316
#15	(resuscitat* or CPR or ventilat*):ti,ab,kw	43533
#16	((tracheal or intratracheal or endotracheal) and intubation*):ti,ab,kw	11386
#17	(triage or overtriage or undertriage):ti,ab,kw	1770
#18	(((damage or urgen* or emergen*) NEAR/2 control) or "initial care"):ti,ab,kw	385
#19	#12 or #13 or #14 or #15 or #16 or #17 or #18	53862
#20	MeSH descriptor: [Tourniquets] explode all trees	560
#21	MeSH descriptor: [Fracture Fixation] explode all trees	1878
#22	(tourniquet* or (pelvi* NEXT binder*) or (pelvi* NEXT closure) or (pelvi* NEXT ring*) or packing or emboli* or ((device* or wound*) NEAR/3 compression*) or ((fracture* or internal* or external*) NEAR/3 fixat*)):ti,ab,kw	23026
#23	MeSH descriptor: [Pelvic Bones] explode all trees	564
#24	MeSH descriptor: [Pubic Symphysis] explode all trees	14
#25	MeSH descriptor: [Sacroiliac Joint] explode all trees	117

#26	MeSH descriptor: [Hip Fractures] explode all trees	1788
#27	MeSH descriptor: [Embolization, Therapeutic] explode all trees	825
#28	(pelvic or pelvis or hip or hips or pubic* or sacroiliac* or symphys* or rib or "chest wall" or ab):ti and (fracture* or injur* or trauma* or disrupt* or instability):ti,ab,kw	4169
#29	#20 or #21 or #22 or #23 or #24 or #25 or #26 or #27 or #28	27898
#30	MeSH descriptor: [Tomography, X-Ray Computed] explode all trees	5364
#31	MeSH descriptor: [Ultrasonography] explode all trees	14448
#32	MeSH descriptor: [Angiography] explode all trees	7497
#33	((comput* NEAR/2 tomograph*) or CT or ultrasonograph* or sonograph* or eFAST or “early FAST” or ultrasound* or (ultra NEXT sound*) or imaging):ti,ab,kw	181407
#34	(angiogra* or angioemboli*):ti,ab,kw	20230
#35	#30 or #31 or #32 or #33 or #34	194357
#36	MeSH descriptor: [Hematologic Tests] explode all trees	12070
#37	MeSH descriptor: [Blood Coagulation Tests] explode all trees	2089
#38	MeSH descriptor: [Point-of-Care Testing] explode all trees	95
#39	(((“point of care” or clotting or platelet* or laborator* or coagulat* or hematocrit or haematocrit or hemoglobin or haemoglobin or hematolog* or haematolog*) NEAR/6 (test or tests or testing or parameter* or monitoring or measur* or tool or tools)) or (blood NEXT test*) or POCT or (“serum lactate” or “admission lactate” or “base deficit” or "base excess")):ti,ab,kw	43333
#40	(APTT or “activated partial thromboplastin time” or thromboelastograph* or TEG or thromboelastometr* or ROTEM or Sonoclot or FIBTEM or “multiple electrode impedance aggregometry” or MEA or “platelet function analyser” or PFA-100 or “platelet reactivity assay” or VerifyNow* or “vasodilator stimulated phosphoprotein” or VASP or D-dimer or “early coagulation” or “conventional coagulation” or CCT or viscoelastic* or VHA or (visco NEXT haemostatic*) or (visco NEXT hemostatic*) or viscohaemostatic* or viscohemostatic*):ti,ab,kw	8030
#41	#36 or #37 or #38 or #39 or #40	58488
#42	MeSH descriptor: [Blood Pressure] explode all trees	28306
#43	MeSH descriptor: [Hematocrit] explode all trees	1565
#44	MeSH descriptor: [Hypotension] explode all trees	2325
#45	MeSH descriptor: [Vasoconstrictor Agents] explode all trees	1882
#46	MeSH descriptor: [Fluid Therapy] explode all trees	1770
#47	MeSH descriptor: [Hypertonic Solutions] explode all trees	732
#48	MeSH descriptor: [Isotonic Solutions] explode all trees	1047
#49	MeSH descriptor: [Crystalloid Solutions] explode all trees	650

#50	MeSH descriptor: [Colloids] explode all trees	6928
#51	MeSH descriptor: [Erythrocyte Transfusion] explode all trees	636
#52	MeSH descriptor: [Hypothermia, Induced] explode all trees	978
#53	((blood NEXT pressure*) or hematocrit or haematocrit or hypotension or vasopressor* or vasoconstrict* or (inotropic NEXT agent*) or inotropes or (fluid NEXT therap*) or (fluid NEXT resuscitation*) or (volume NEXT resuscitation*) or (fluid NEXT replacement*) or (volume NEXT replacement*) or (rehydration NEXT therap*) or (sodium NEXT chlorid*) or (saline NEXT solution*) or (hypertonic NEXT solution*) or (isotonic NEXT solution*) or (hypotonic NEXT solution*) or cristalloid* or crystalloid* or colloid* or (plasma NEXT substitut*) or (ringer* NEXT solution*) or (ringer* NEXT lactat*) or (blood NEXT substitute*) or erythrocyte* or hypotherm* or "balanced solution" or Hartmann or plasmalyte):ti,ab,kw	146190
#54	#42 or #43 or #44 or #45 or #46 or #47 or #48 or #49 or #50 or #51 or #52 or #53	153068
#55	MeSH descriptor: [Blood Coagulation] explode all trees	2415
#56	MeSH descriptor: [Hemostasis] explode all trees	4990
#57	MeSH descriptor: [Hemostatics] explode all trees	1806
#58	MeSH descriptor: [Fibrinogen] explode all trees	1745
#59	MeSH descriptor: [Antifibrinolytic Agents] explode all trees	858
#60	MeSH descriptor: [Tranexamic Acid] explode all trees	1232
#61	MeSH descriptor: [Prothrombin] explode all trees	389
#62	(coagula* or haemosta* or hemosta* or fibrinogen* or antifibrinolytic* or (anti NEXT fibrinolytic*) or (tranexamic NEXT acid*) or prothrombin* or (blood NEXT clot*)):ti,ab,kw	31482
#63	#55 or #56 or #57 or #58 or #59 or #60 or #61 or #62	33352
#64	MeSH descriptor: [Anticoagulants] explode all trees	4876
#54	#42 or #43 or #44 or #45 or #46 or #47 or #48 or #49 or #50 or #51 or #52 or #53	153068
#66	MeSH descriptor: [Factor Xa Inhibitors] explode all trees	608
#67	(anticoagula* or (anti NEXT coagula*) or DOAC* or antiplatelet* or (anti NEXT platelet*) or antithromb* or (anti NEXT thromb*) or “antifactor Xa” or “anti factor Xa” or ((platelet* or thrombin* or “factor Xa”) NEAR/6 inhibitor*) or rivaroxaban or apixaban or edoxaban or betrixaban or dabigatran or "andexanet alfa" or heparin or TXA or idarucizumab or PCC or PCCs or (prothrombin NEXT complex NEXT concentrate*) or reversal or reversing or residual):ti,ab,kw	60273
#68	MeSH descriptor: [Intermittent Pneumatic Compression Devices] explode all trees	153
#69	MeSH descriptor: [Stockings, Compression] explode all trees	268
#70	(thromboprophylaxis or (thrombosis NEAR/1 prevent*) or (intermittent NEXT pneumatic NEXT compression*) or stocking* or (“vena cava” NEAR/2 filter*)):ti,ab,kw	3994

#71	#64 or #65 or #66 or #67 or #68 or #69 or #70	62213
#72	MeSH descriptor: [Plasma] explode all trees	1186
#73	MeSH descriptor: [Platelet Transfusion] explode all trees	333
#74	MeSH descriptor: [Blood Transfusion] explode all trees	3702
#75	MeSH descriptor: [Blood Platelets] explode all trees	2022
#76	MeSH descriptor: [Calcium Chloride] explode all trees	92
#77	MeSH descriptor: [Deamino Arginine Vasopressin] explode all trees	398
#78	(plasma or FFP or erythrocyte* or (red NEXT blood NEXT cell*) or platelet* or thrombocyte* or (blood NEXT transfusion*) or (blood NEXT product*)):ti,ab,kw	145799
#79	(transfusion NEAR/2 (trigger* or mass* or management or requirement*)):ti,ab,kw	2581
#80	(cryoprecipitate* or cryo or thrombocytopenia or thrombocytopaenia or "calcium chloride" or hypocalcemia or hypocalcaemia or desmopressin or PCC or PCCs or (prothrombin NEXT complex NEXT concentrate*) or rFVIIa or “recombinant factor VIIa” or (“recombinant activated” NEAR/2 “factor VII”) or FXIII or “factor XIII”):ti,ab,kw	13936
#81	MeSH descriptor: [Operative Blood Salvage] explode all trees	47
#82	(((Cell or blood) NEAR/3 salvage) or "cell saver" or autotransfus* or (auto NEXT transfus*) or (autologous NEAR/3 blood NEAR/3 (transfus* or retransfus*))):ti,ab,kw	1389
#83	#72 or #73 or #74 or #75 or #76 or #77 or #78 or #79 or #80 or #81 or #82	156507
#84	MeSH descriptor: [Time-to-Treatment] explode all trees	427
#85	MeSH descriptor: [Emergency Service, Hospital] explode all trees	2665
#86	MeSH descriptor: [Trauma Centers] explode all trees	192
#87	(((trauma NEAR/2 (care or centre* or centre* or unit* or facilit*)) and (transport* or transfer* or time or timing or designation)) or (time NEAR/2 treatment*)):ti,ab,kw	14633
#88	#84 or #85 or #86 or #87	17213
#89	MeSH descriptor: [Trauma Severity Indices] explode all trees	1259
#90	(((injury or trauma or shock) NEXT severity) or ISS or “mechanism of injury” or “magnitude of injury” or “shock index” or (assess* NEXT/3 (“high velocity” or speed or “high energy” or “fall height”)) or ((shock* or ATLS or injur* or trauma*) NEXT/3 classification*)):ti,ab,kw	3047
#91	#89 or #90	3533
#92	MeSH descriptor: [Practice Guidelines as Topic] explode all trees	1670
#93	MeSH descriptor: [Clinical Protocols] explode all trees	19416
#94	MeSH descriptor: [Decision Making] explode all trees	4241
#95	MeSH descriptor: [Guideline Adherence] explode all trees	1112
#96	MeSH descriptor: [Patient Care Bundles] explode all trees	37

#97	(guideline* or "decision making" or protocol* or checklist* or (assessment NEXT tool*) or ((treatment or care or patient) NEAR/2 bundle*) or "quality control" or (quality NEXT program*)):ti,ab,kw	195920
#98	((treat* or therap* or care) NEAR/3 ("goal directed")):ti,ab,kw	746
#99	((treat* or therap* or care) NEAR/10 algorithm*):ti,ab,kw	3614
#100	#92 or #93 or #94 or #95 or #96 or #97 or #98 or #99	200464
#101	#19 or #29 or #35 or #41 or #54 or #63 or #71 or #83 or #88 or #91 or #100	689576
#102	#11 and #101	4018 Full result
#103	#11 and #19	878 Bundle 1
#104	#11 and #29	559 Bundle 2
#105	#11 and #35	1153 Bundle 3
#106	#11 and #41	332 Bundle 4
#107	#11 and #54	837 Bundle 5
#108	#11 and #63	611 Bundle 6
#109	#11 and #71	548 Bundle 7
#110	#11 and #83	827 Bundle 8
#111	#11 and #88	183 Bundle 9
#112	#11 and #91	375 Bundle 10
#113	#11 and #100	1369 Bundle 12
#114	#103 or #104 or #105 or #106 or #107 or #108 or #109 or #110 or #111 or #112 or #113	4018 All bundles full result

Additional file 2: Search bundles, structured literature search strategies and results

Table 4: Search strategy queries used in Epistemonikos.

Systematic reviews, 2018-2021

Search number	Query	Results 03 Dec 2021
#1	((bleed* OR haemorrhag* OR hemorrhag* OR “blood loss”)<br>AND<br>(injur* OR trauma*)<br>AND<br>(care OR treatment OR control OR therapy OR test* OR anticoagula* OR coagula* OR imaging OR transfusion* OR ultrasound OR CT OR timing OR fluid* OR hypotension OR fracture*))<br><br>OR<br>((“multiple trauma” OR “multiple injury” OR “multiple injuries” OR “major trauma” OR “major injury” OR “major injuries” OR “severe trauma” OR “severe injury” OR “severe injury” OR polytrauma OR “poly trauma”)<br>AND<br>(care OR treatment OR control OR therapy OR test* OR anticoagula* OR coagula* OR imaging OR transfusion* OR ultrasound OR CT OR timing OR fluid* OR hypotension OR fracture*))	598 references

Table 5: Database search results (number identified) by search bundle.

**Search bundle**	**Raw results**				**Screened results**	
	Medline	***CENTRAL***	***Total***	***De-duplicated***	***Excluded***	***Pre-selected***
Bundle 1	1290	878	2168	2112	1773	237
Bundle 2	1973	559	2532	2430	2159	189
Bundle 3	2703	1153	3856	3780	3400	267
Bundle 4	840	332	1172	1141	977	121
Bundle 5	1195	837	2032	1972	1735	121
Bundle 6	1740	611	2351	2254	1987	141
Bundle 7	1586	548	2134	2049	1780	152
Bundle 8	2209	827	3036	2943	2272	528
Bundle 9	559	183	742	735	672	36
Bundle 10	1265	375	1640	1608	1414	133
Bundle 12	1743	1369	3112	3027	2772	102
Epistemonikos			598	598	431	163
**Total references**			**25373**	**24649**	**21372**	**2190**

The search in the Cochrane Central Register of Controlled Trials (CENTRAL) database was performed with a broad population (patients with trauma and haemorrhage, or patients with multiple, severe, major trauma/injury [without mentioning haemorrhage]).

The search in Medline (OvidSP) was performed with a narrower population (patients with trauma and haemorrhage) and with filters for relevant study types to increase precision.

Bundle 11 was omitted from the search strategy due to the volume of results.
