# Supplementary material for: The European guideline on management of major bleeding and coagulopathy following trauma: sixth edition
Source: Crit Care. 2023 Mar 1;27:80. doi: 10.1186/s13054-023-04327-7 (PMC9977110; doi:10.1186/s13054-023-04327-7)
Supplement: Supplementary file 3 — Additional file 3. Summary of recommendations. [file 13054_2023_4327_MOESM3_ESM.pdf]

The European guideline on management of major bleeding and coagulopathy following trauma: Sixth edition

Additional file 3: Summary of recommendations

#	Title	Recommendation	Author(s)	Reviewer(s)	Grade	Vote % agree [agree; abstain; disagree]*
**I. Initial resuscitation and prevention of further bleeding**						
R1	Minimal elapsed time	• We recommend that severely injured patients be transported directly to an appropriate trauma facility.	BB	LR	1B	94 [16; 1; 0]
		• We recommend that the time elapsed between injury and bleeding control be minimised.	BB	LR	1B	100 [17; 0; 0]
R2	Local bleeding management	• We recommend local compression of open wounds to limit life-threatening bleeding.	LR	BB	1B	100 [14; 0; 0]
		• We recommend adjunct tourniquet use to stop life-threatening bleeding from open extremity injuries in the pre-surgical setting.	LR	BB	1B	100 [14; 0; 0]
R3	Ventilation	• We recommend that endotracheal intubation or alternative airway management be performed without delay in the presence of airway obstruction, altered consciousness [Glasgow Coma Scale ≤8], hypoventilation or hypoxaemia.	JLV	DC	1B	100 [17; 0; 0]
		• We recommend the avoidance of hypoxaemia.	JLV	DC	1A	100 [17; 0; 0]
		• We suggest the avoidance of hyperoxaemia, except in the presence of imminent exsanguination.	JLV	DC	2B	94 [16; 1; 0]
		• We recommend normoventilation of trauma patients.	JLV	DC	1B	100 [17; 0; 0]
		• We suggest hyperventilation as a life-saving measure in the presence of signs of cerebral herniation.	JLV	DC	2C	94 [16; 1; 0]
R4	Pre-hospital blood product use	• No recommendation.	MM	JD, AH, VC		

II. Diagnosis and monitoring of bleeding						
R5	Initial assessment	• We recommend that the physician clinically assess the extent of traumatic haemorrhage using a combination of patient physiology, anatomical injury pattern, mechanism of injury and the patient response to initial resuscitation.	MM	JD, AH	1C	100 [13; 0; 0]
		• We recommend that the shock index and/or narrow pulse pressure be used to assess the degree of hypovolaemic shock and transfusion requirements.	MM	JD, AH	1C	100 [13; 0; 0]
R6	Immediate intervention	• We recommend that patients with an obvious bleeding source and those presenting with haemorrhagic shock in extremis and a suspected source of bleeding undergo an immediate bleeding control procedure.	LR	BB	1B	85 [11; 1; 1]
R7	Further investigation	• We recommend that patients with an unidentified source of bleeding, but without a need for immediate bleeding control, undergo immediate further investigation to determine the bleeding source.	DC	BB	1C	94 [16; 1; 0]
R8	Imaging	• We suggest the use of pre-hospital ultrasonography for the detection of haemo-/pneumothorax, haemopericardium and/or free abdominal fluid in patients with thoracoabdominal injuries, if feasible without delaying transport.	MM	BB	2B	100 [15; 0; 0]
		• We recommend the use of point-of-care ultrasonography, including focused assessment with sonography in trauma, in patients with thoracoabdominal injuries.	MM	BB	1C	100 [15; 0; 0]
		• We recommend early imaging using contrast-enhanced whole-body CT for the detection and identification of type of injury and potential source of bleeding.	MM	BB	1B	94 [14; 1; 0]
R9	Haemoglobin	• We recommend the use of repeated Hb and/or Hct measurements as a laboratory marker for bleeding, as an initial value in the normal range may mask early-phase bleeding.	VC	CMS	1B	100 [14; 0; 0]

R10	Blood lactate and base deficit	• We recommend blood lactate as a sensitive test to estimate and monitor the extent of bleeding and tissue hypoperfusion; in the absence of lactate measurements, base deficit may represent a suitable alternative.	JLV	VC	1B	100 [14; 0; 0]
R11	Coagulation monitoring	• We recommend the early and repeated monitoring of haemostasis, using either a traditional laboratory determination such as prothrombin time international normalised ratio, Clauss fibrinogen level and platelet count and/or point-of-care prothrombin time /international normalised ratio and/or a viscoelastic method.	NC	CMS	1C	100 [14; 0; 0]
R12	Platelet function monitoring	• We recommend that the routine use of point-of-care platelet function devices for platelet function monitoring in trauma patients on antiplatelet therapy or with suspected platelet dysfunction be avoided.	DF	CMS, NC	1C	100 [14; 0; 0]

*Not all authors were present for voting on every recommendation, therefore percentage reflects agreement based on those authors present.

III. Tissue oxygenation, volume, fluids and temperature						
R13	Volume replacement and target blood pressure	• In the initial phase following trauma we recommend the use of a restricted volume replacement strategy with a target systolic blood pressure of 80–90 mmHg (mean arterial pressure 50–60 mmHg) until major bleeding has been stopped without clinical evidence of brain injury.	RR	JD, AH	1B	100 [13; 0; 0]
		• In patients with severe traumatic brain injury (Glasgow Coma Scale ≤8), we recommend that a mean arterial pressure ≥80 mmHg be maintained.	RR	JD, AH	1C	92 [12; 1; 0]
R14	Vasopressors and inotropic agents	• If a restricted volume replacement strategy does not achieve the target blood pressure, we recommend the administration of noradrenaline in addition to fluids to maintain target arterial pressure.	RR	JD, AH	1C	100 [13; 0; 0]
		• We recommend infusion of dobutamine in the presence of myocardial dysfunction.	RR	JD, AH	1C	100 [13; 0; 0]
R15	Type of fluid	• We recommend that fluid therapy using a 0.9% sodium chloride or balanced crystalloid solution be initiated in the hypotensive bleeding trauma patient.	RR	JLV	1B	92 [11; 1; 0]
		• We recommend that hypotonic solutions such as Ringer's lactate be avoided in patients with severe head trauma.	RR	JLV	1B	100 [12; 0; 0]
		• We recommend that the use of colloids be restricted due to the adverse effects on haemostasis.	RR	JLV	1C	83 [10; 2; 0]
R16	Erythrocytes	• If erythrocyte transfusion is necessary, we recommend a target haemoglobin of 70 to 90 g/L.	JD, AH	DRS	1C	100 [14; 0; 0]
R17	Cell salvage	• We suggest that cell salvage be considered in the presence of severe bleeding from an abdominal, pelvic or thoracic cavity.	LM	RR	2B	92 [12; 1; 0]
R18	Temperature management	• We recommend early application of measures to reduce heat loss and warm the hypothermic patient to achieve and maintain normothermia.	RR	VC	1C	100 [12; 0; 0]

IV. Rapid control of bleeding						
R19	Damage-control surgery	• We recommend damage control surgery in the severely injured patient presenting with haemorrhagic shock, signs of ongoing bleeding, coagulopathy and/or combined abdominal vascular and pancreatic injuries.	LR	BB	1B	88 [14; 1; 1]
		• Other factors that should trigger a damage control approach are hypothermia, acidosis, inaccessible major anatomic injury, a need for time-consuming procedures.	LR	BB	1C	94 [15; 1; 0]
		• We recommend primary definitive surgical management in the absence of any of the factors above.	LR	BB	1C	88 [14; 2; 0]
R20	Pelvic ring closure and stabilisation	• We recommend the adjunct use of a pelvic binder in the pre-hospital setting to limit life-threatening bleeding in the presence of a suspected pelvic fracture.	RK	LR	1C	100 [17; 0; 0]
		• We recommend that patients with pelvic ring disruption in haemorrhagic shock undergo pelvic ring closure and stabilization as early as possible.	RK	LR	1B	94 [16; 1; 0]
R21	Packing, embolisation and surgery	• We recommend temporary extra-peritoneal packing when bleeding is ongoing and/or when angioembolisation cannot be achieved in a timely manner. Extra-peritoneal packing can be combined with open abdominal surgery when necessary.	RK	LR	1C	100 [16; 0; 0]
		• We suggest that resuscitative endovascular balloon occlusion of the aorta be considered in patients with noncompressible life-threatening traumatic haemorrhage to bridge the gap between haemodynamic collapse and haemorrhage control.	RK	LR	2C	100 [16; 0; 0]
R22	Local haemostatic measures	• We recommend the use of topical haemostatic agents in combination with other surgical measures or with packing for venous or moderate arterial bleeding associated with parenchymal injuries.	LR	RK	1B	100 [16; 0; 0]

V. Initial management of bleeding & coagulopathy						
R23	Antifibrinolytic agents	• We recommend that tranexamic acid be administered to the trauma patient who is bleeding or at risk of significant bleeding as soon as possible, if feasible en route to the hospital, and within 3 h after injury at a loading dose of 1 g infused over 10 min, followed by an i.v. infusion of 1 g over 8 h.	NC	DRS	1A	100 [14; 0; 0]
		• We recommend that the administration of tranexamic acid not await results from a viscoelastic assessment.	NC	DRS	1B	100 [14; 0; 0]
R24	Coagulation support	• We recommend that monitoring and measures to support coagulation be initiated immediately upon hospital admission.	DRS	CMS	1B	100 [16; 0; 0]
R25	Initial coagulation resuscitation	• In the initial management of patients with expected massive haemorrhage, we recommend one of the two following strategies:	OG	JD, AH, NC		
		o Fibrinogen concentrate or cryoprecipitate and RBC.	OG	JD, AH, NC	1C	100 [15; 0; 0]
		o FFP or pathogen-inactivated FFP in a FFP:RBC ratio of at least 1:2 as needed.	OG	JD, AH, NC	1C	100 [15; 0; 0]
		• In addition, we suggest a high platelet:RBC ratio.	OG	JD, AH, NC	2B	93 [14; 1; 0]

VI. Further goal-directed coagulation management						
R26	Goal-directed therapy	• We recommend that resuscitation measures be continued using a goal-directed strategy, guided by standard laboratory coagulation values and/or viscoelastic method.	MM	RR, JLV	1B	100 [14; 0; 0]
R27	Fresh frozen plasma-based management	• If a FFP-based coagulation resuscitation strategy is used, we recommend that further use of FFP be guided by standard laboratory coagulation screening parameters (prothrombin time and/or activated partial thromboplastin time >1.5 times normal and/or viscoelastic evidence of a coagulation factor deficiency).	OG	DF, NC	1C	100 [12; 0; 0]
		• We recommend that the use of FFP be avoided for the correction of hypofibrinogenaemia if fibrinogen concentrate and/or cryoprecipitate are available.	OG	DF, NC	1C	92 [11; 0; 1]
R28	Coagulation factor concentrate-based management	• If a coagulation factor concentrate -based strategy is used, we recommend treatment with factor concentrates based on standard laboratory coagulation parameters and/or viscoelastic evidence of a functional coagulation factor deficiency.	DRS	NC	1C	100 [15; 0; 0]
		• Provided that fibrinogen levels are normal, we suggest that prothrombin complex concentrate is administered to the bleeding patient based on evidence of delayed coagulation initiation using viscoelastic method.	DRS	NC	2C	93 [14; 1; 0]
		• We suggest that monitoring of FXIII be included in coagulation support algorithms and that FXIII be supplemented in bleeding patients with a functional FXIII deficiency.	DRS	NC	2C	87 [13; 2; 0]
R29	Fibrinogen supplementation	• We recommend treatment with fibrinogen concentrate or cryoprecipitate if major bleeding is accompanied by hypofibrinogenaemia (viscoelastic signs of a functional fibrinogen deficit or a plasma Clauss fibrinogen level ≤1.5 g/L).	CMS	DF, NC	1C	67 [10; 0; 5]

		• We suggest an initial fibrinogen supplementation of 3–4 g. This is equivalent to 15–20 single donor units of cryoprecipitate or 3–4 g fibrinogen concentrate. Repeat doses should be guided by viscoelastic method and laboratory assessment of fibrinogen levels.	CMS	DF, NC	2C	100 [15; 0; 0]
R30	Platelets	• We suggest that platelets be administered to maintain a platelet count above 50 × 10^9/L in trauma patients with ongoing bleeding and above 100 × 10^9/L in patients with traumatic brain injury.	DF	CMS	2C	100 [14; 0; 0]
		• If administered, we suggest an initial dose of four to eight single platelet units or one aphaeresis pack.	DF	CMS	2B	100 [14; 0; 0]
R31	Calcium	• We recommend that ionised calcium levels be monitored and maintained within the normal range following major trauma and especially during massive transfusion.	AK, DRS	JLV, NC	1C	100 [15; 0; 0]
		• We recommend the administration of calcium chloride to correct hypocalcaemia.	AK, DRS	JLV, NC	1C	100 [15; 0; 0]
R32	Recombinant activated coagulation factor VII	• We do not recommend the use of recombinant activated coagulation factor VII as first-line treatment.	VC	DRS	1B	100 [13; 0; 0]
		• We suggest that the off-label use of recombinant activated coagulation factor VII be considered only if major bleeding and traumatic coagulopathy persist despite all other attempts to control bleeding, systemic homeostasis and best-practice use of conventional haemostatic measures.	VC	DRS	2C	100 [13; 0; 0]

VII. Management of antithrombotic agents						
R33	Reversal of vitamin K-dependent oral anticoagulants	• In the bleeding trauma patient, we recommend the emergency reversal of vitamin K-dependent oral anticoagulants with the early use of both prothrombin complex concentrate and 5–10 mg i.v. phytomenadione (vitamin K_1).	BJH	DRS	1A	93 [13; 1; 0]
R34	Management of direct oral anticoagulants - factor Xa inhibitors	• We suggest the measurement of plasma levels of oral direct anti-factor Xa agents such as apixaban, edoxaban or rivaroxaban in patients treated or suspected of being treated with one of these agents.	AK, DRS	BJH	2C	100 [14; 0; 0]
		• We suggest that measurement of anti-Xa activity be calibrated for the specific agent. If not possible or available, we suggest low molecular weight heparin - calibrated anti-Xa assays as a reliable alternative.	AK, DRS	BJH	2C	100 [14; 0; 0]
		• If bleeding is life-threatening in the presence of an apixaban or rivaroxaban effect, especially in patients with traumatic brain injury, we suggest reversal with andexanet alfa.	AK, DRS	BJH	2C	100 [14; 0; 0]
		• If andexanet alfa is not available, or in patients receiving edoxaban, we suggest the administration of prothrombin complex concentrate (25–50 U/kg).	AK, DRS	BJH	2C	93 [13; 0; 1]
R35	Management of director oral anticoagulants - direct thrombin inhibitors	• We suggest the measurement of dabigatran plasma levels using diluted thrombin time in patients treated or suspected of being treated with dabigatran.	AK, DRS	BJH	2C	93 [13; 1; 0]
		• If measurement is not possible or available, we suggest measurement of the standard thrombin time to allow a qualitative estimation of the presence of dabigatran.	AK, DRS	BJH	2C	100 [14; 0; 0]
		• If bleeding is life-threatening in those receiving dabigatran, we recommend treatment with idarucizumab (i.v. 5 g).	AK, DRS	BJH	1C	100 [19; 0; 0]
R36	Antiplatelet agents	• We recommend that routine platelet transfusion be avoided in patients with ongoing bleeding who have been treated with antiplatelet agents.	DF	CMS	1C	100 [13; 0; 0]

**VIII. Thromboprophylaxis**						
R37	Thromboprophylaxis	• We recommend early initiation of mechanical thromboprophylaxis with intermittent pneumatic compression while the patient is immobile and has a bleeding risk.	BJH	JD, AH	1C	93 [13; 0; 1]
		• We recommend combined pharmacological and intermittent pneumatic compression thromboprophylaxis within 24 h after bleeding has been controlled and until the patient is mobile.	BJH	JD, AH	1B	86 [12; 1; 1]
		• We do not recommend the use of graduated compression stockings for thromboprophylaxis.	BJH	JD, AH	1C	100 [14; 0; 0]
		• We do not recommend the routine use of inferior vena cava filters as thromboprophylaxis.	BJH	JD, AH	1C	100 [14; 0; 0]

**IX. Guideline implementation and quality control**						
R38	Guideline implementation	• We recommend the local implementation of evidence-based guidelines for management of the bleeding trauma patient.	DRS, RR	LR, BB	1B	100 [14; 0; 0]
R39	Assessment of bleeding control and outcome	• We recommend that local clinical quality and safety management systems include parameters to assess key measures of bleeding control and outcome.	DRS, RR	LR, BB	1B	100 [15; 0; 0]
