# Supplementary material for: The European guideline on management of major bleeding and coagulopathy following trauma: sixth edition
Source: Crit Care. 2023 Mar 1;27:80. doi: 10.1186/s13054-023-04327-7 (PMC9977110; doi:10.1186/s13054-023-04327-7)
Supplement: Supplementary file 4 — Additional file 4. Summary of treatment modalities for the bleeding trauma patient included in this guideline (A0 poster). [file 13054_2023_4327_MOESM4_ESM.pdf]

The European guideline on management of major bleeding and coagulopathy following trauma: sixth edition (2023)

Rolf Rossaint, Arash Afshari, Bertil Bouillon, Vladimir Cerny, Diana Cimpoesu, Nicola Curry, Jacques Duranteau, Daniela Filipescu, Oliver Grottke, Lars Grønlykke, Anatole Harrois, Beverley J. Hunt, Alexander Kaserer, Radko Komadina, Mikkel Herold Madsen, Marc Maegele, Lidia Mora, Louis Riddez, Carolina S Romero, Charles-Marc Samama, Jean-Louis Vincent, Sebastian Wiberg, Donat R. Spahn

Crit Care 27 (2023)

I. Initial resuscitation & prevention of further bleeding

R1 Minimal elapsed time
***
Severely injured patients should be transported directly to an appropriate trauma facility. The time elapsed between injury and bleeding control should be minimised.

R2 Local bleeding management
***
Local compression should be applied to open wounds to limit life-threatening bleeding. A tourniquet should be applied to open extremity injuries as an adjunct in the pre-surgical setting.

R3 Ventilation
***
Endotracheal intubation or alternative airway management should be performed without delay in the presence of airway obstruction, altered consciousness [GCS ≤8], hypoventilation or hypoxaemia. Hypoxaemia should be avoided and normoventilation applied. Hyperoxaemia may be applied in the presence of imminent exsanguination. Hyperventilation may be applied as a life-saving measure in the presence of signs of cerebral herniation.

R4 Pre-hospital blood product use
***
No recommendation at this time.

II. Diagnosis & monitoring of bleeding

R5 Initial assessment
***
The extent of traumatic haemorrhage should be assessed using a combination of patient physiology, anatomical injury pattern, Mechanism of injury and response to initial resuscitation. The shock index and/or narrow pulse pressure should be used to assess hypovolaemic shock and transfusion requirements.

R6 Immediate intervention
***
Patients presenting with an obvious bleeding source or in haemorrhagic shock in extremis and a suspected bleeding source should undergo an immediate bleeding control procedure.

R7 Further investigation
***
Patients with an unidentified source of bleeding, but without a need for immediate bleeding control, should undergo immediate further investigation.

R8 Imaging
***
If feasible without delaying transport, pre-hospital ultrasonography may be used to detect haemo-/pneumothorax, haemopericardium and/or free abdominal fluid in patients with thoracoabdominal injuries. Point-of-care ultrasonography should be used in patients with thoracoabdominal injuries. Early imaging using contrast-enhanced whole-body CT should be used to detect and identify injury type and bleeding source.

R9 Haemoglobin
***
Repeated Hb and/or Hct measurements should be employed to detect ongoing bleeding.

R10 Blood lactate & base deficit
***
Blood lactate should be used to estimate and monitor the extent of bleeding and tissue hypoperfusion; if unavailable, base deficit may represent a suitable alternative.

R11 Coagulation monitoring
***
Early, repeated haemostasis monitoring, including laboratory measurements (PT/INR, fibrinogen, platelets, fibrinogen) and/or point-of-care PT/INR and/or viscoelastic methods, should be employed.

R12 Platelet function monitoring
***
The routine use of point-of-care platelet function monitoring devices should be avoided in trauma patients on antiplatelet therapy or with suspected platelet dysfunction.

III. Tissue oxygenation, volume, fluids & temperature

R13 Volume replacement & target blood pressure
***
A restricted volume replacement strategy with a target systolic blood pressure of 80–90 mmHg (MAP 50–60 mmHg) should be employed until major bleeding has been stopped in the initial phase following trauma without clinical evidence of brain injury. A mean arterial pressure ≥80 mmHg should be maintained in patients with severe TBI (GCS ≤8).

R16 Erythrocytes
***
If erythrocyte transfusion is necessary, treatment should aim to achieve a target Hb of 70-90 g/L.

R14 Vasopressors & inotropic agents
***
Noradrenaline should be administered in addition to fluids if a restricted volume replacement strategy fails to maintain target arterial pressure. Dobutamine should be infused in the presence of myocardial dysfunction.

R17 Cell salvage
***
Cell salvage may be considered in the presence of severe bleeding from an abdominal, pelvic or thoracic cavity.

R15 Type of fluid
***
Fluid therapy using a 0.9% NaCl or balanced crystalloid solution should be initiated in the hypotensive bleeding trauma patient. Hypotonic solutions such as Ringer's lactate should be avoided in patients with severe head trauma. The use of colloids should be restricted due to the adverse effect on haemostasis.

R18 Temperature management
***
Early measures to reduce heat loss and warm the hypothermic patient should be employed to achieve and maintain normothermia.

IV. Rapid control of bleeding

R19 Damage-control surgery
***
Damage-control surgery should be performed in the severely injured patient presenting with haemorrhagic shock, signs of ongoing bleeding, coagulopathy and/or combined abdominal vascular and pancreatic injuries. Hypothermia, acidosis, inaccessible major anatomic injury or a need for time-consuming procedures should also trigger a damage-control approach. Primary definitive surgical Management should be performed in the absence of any of these factors.

R20 Pelvic ring closure & stabilisation
***
In the pre-hospital setting, adjunct use of a pelvic binder should be used to limit life-threatening bleeding in the presence of a suspected pelvic fracture. Patients with pelvic ring disruption in haemorrhagic shock should undergo early pelvic ring closure and stabilisation.

R21 Embolisation, packing, surgery & REBOA
***
When bleeding is ongoing and/or angioembolisation cannot be achieved in a timely manner, temporary extra-peritoneal packing should be applied, combined with open abdominal surgery when necessary. Resuscitative endovascular balloon occlusion of the aorta may be considered in patients with noncompressible life-threatening traumatic haemorrhage.

R22 Local haemostatic measures
***
Topical haemostatic agents should be employed in combination with other surgical measures or with packing for venous or moderate arterial bleeding associated with parenchymal injuries.

V. Initial management of bleeding & coagulopathy

R23 Antifibrinolytic agents
***
Tranexamic acid should be administered to the trauma patient who is bleeding or at risk of significant bleeding as soon as possible, en route to the hospital if feasible, and within 3 h at a loading dose of 1 g infused over 10 min, followed by an intravenous infusion of 1 g over 8 h. Tranexamic acid administration should not await viscoelastic assessment results.

R24 Coagulation support
***
Monitoring and measures to support coagulation should be initiated immediately upon hospital admission.

R25 Initial coagulation resuscitation
***
The initial coagulation resuscitation strategy for patients with expected massive haemorrhage should comprise either:
fibrinogen concentrate or cryoprecipitate and pRBC
OR
FFP or pathogen-inactivated FFP in a FFP:pRBC ratio of at least 1:2 as needed.
A high platelet:pRBC ratio may be applied.

VI. Further goal-directed coagulation management

R26 Goal-directed therapy
***
Resuscitation measures should be continued using a goal-directed strategy guided by standard laboratory coagulation values and/or viscoelastic monitoring.

R27 Fresh frozen plasma-based management
***
Further use of FFP should be guided by standard laboratory coagulation parameters (PT and/or APTT >1.5 times normal and/or viscoelastic evidence of a coagulation factor deficiency). FFP use should be avoided for the correction of hypofibrinogenaemia if fibrinogen concentrate and/or cryoprecipitate are available.

R28 Coagulation factor concentrate-based management
***
Factor concentrates should be administered based on standard laboratory coagulation parameters and/or viscoelastic evidence of a functional coagulation factor deficiency. If fibrinogen levels are normal, PCC may be administered based on viscoelastic evidence of delayed coagulation initiation. FXIII monitoring may be included in coagulation support algorithms and FXIII supplemented in bleeding patients with a functional FXIII deficiency.

R29 Fibrinogen supplementation
***
Fibrinogen concentrate or cryoprecipitate should be administered if major bleeding is accompanied by hypofibrinogenaemia (viscoelastic signs or plasma fibrinogen level ≤1.5 g/L). An initial fibrinogen supplementation of 3–4 g, equivalent to 15–20 single donor units of cryoprecipitate or 3–4 g fibrinogen concentrate, may be administered. Repeat doses should be guided by viscoelastic monitoring and laboratory assessment of fibrinogen levels.

R30 Platelets
***
Platelets may be administered to patients with ongoing bleeding to maintain a platelet count above 50 × 10⁹/L and above 100 × 10⁹/L in patients with TBI. If administered, an initial dose of 4–8 single platelet units or one aphaeresis pack may be used.

R31 Calcium
***
Ionised calcium levels should be monitored and maintained within the normal range following major trauma and during massive transfusion. Calcium chloride should be administered to correct hypocalcaemia.

R32 Recombinant activated coagulation factor VII
***
rFVIIa should not be used as first-line treatment. Off-label use of rFVIIa may be considered only if major bleeding and traumatic Coagulopathy persist despite all other attempts to control bleeding, systemic homeostasis and best practice use of conventional haemostatic measures.

VII. Management of antithrombotic agents

R33 Reversal of vitamin K-dependent oral anticoagulants
***
Emergency reversal of vitamin K-dependent oral anticoagulants in the bleeding trauma patient should be accomplished with early use of both PCC and 5–10 mg i.v. phytomenadione (vitamin K_1).

R34 Management of direct oral anticoagulants – factor Xa inhibitors
***
Plasma levels of oral direct anti-factor Xa agents such as apixaban, edoxaban or rivaroxaban may be measured in patients treated or suspected of being treated with one of these agents. Measurement of anti-Xa activity may be calibrated for the specific agent. If not possible or available, LMWH-calibrated anti-Xa assays may be used. If life-threatening bleeding occurs in the presence of an apixaban or rivaroxaban effect, especially in patients with TBI, a reversal may be achieved with andexanet alfa. If andexanet alfa is not available, or in patients receiving edoxaban, PCC (25–50 U/kg) may be administered.

R35 Management of direct oral anticoagulants – direct thrombin inhibitors
***
Dabigatran plasma levels may be measured using diluted thrombin time in patients treated or suspected of being treated with dabigatran. If measurement of dabigatran is not possible or available, standard thrombin time may allow a qualitative estimation. Life-threatening bleeding in those receiving dabigatran should be treated with idarucizumab (i.v. 5 g)

R36 Antiplatelet agents
***
Routine platelet transfusion should be avoided in patients with ongoing bleeding who have been treated with antiplatelet agents.

VIII. Thromboprophylaxis

R37 Thromboprophylaxis
***
Early mechanical thromboprophylaxis with intermittent pneumatic compression should be applied while the patient is immobile and has a bleeding risk. Combined pharmacological and intermittent pneumatic compression should be employed within 24 h after bleeding has been controlled and until the patient is mobile. Graduated compression stockings should not be used and inferior vena cava filters should not be routinely employed as thromboprophylaxis.

IX. Guideline implementation & quality control

R38 Guideline implementation
***
Evidence-based guidelines for management of the bleeding trauma patient should be implemented locally.

R39 Assessment of bleeding control & outcome
***
Local clinical quality and safety management systems should include parameters to assess key measures of bleeding control and outcome.

© Task Force for Advanced Bleeding Care in Trauma. All rights reserved. 2023.
